# Molecular architectures of glycosylated dendronized bottle brushes in action: Biocompatibility and anti-amyloidogenic activity of pseudo-glycodendrimers

**DOI:** 10.1016/j.mtbio.2025.101771

**Published:** 2025-05-10

**Authors:** Tom Kösterke, Radika Thakore, Silvia Moreno, Jan Skov Pedersen, Brigitte Voit, Oxana Klementieva, Dietmar Appelhans

**Affiliations:** a Leibniz-Institut für Polymerforschung Dresden e.V., Hohe Straße 6, 01069, Dresden, Germany; b Organic Chemistry of Polymers, TUD Dresden University of Technology, 01062, Dresden, Germany; c Medical Microspectroscopy, Department of Experimental Medical Science, Lund University, 22180, Lund, Sweden; d NanoLund, Lund University, 22180, Lund, Sweden; e Multipark, Lund University, 22180, Lund, Sweden; f Department of Organic and Inorganic Chemistry, University of Alcalá, 28805, Madrid, Spain; g Department of Chemistry and Interdisciplinary Nanoscience Center (iNANO), Aarhus University, Gustav Wieds Vej 14, Aarhus C, DK-8000, Denmark

**Keywords:** Pseudo-glycodendrimers, Anti-amyloidogenic agent, Dendronized bottle brushes, Biocompatibility, Post-modification

## Abstract

Glycopolymers are versatile materials for biomedical and healthcare applications, e.g., as carrier and polymeric therapeutics. Especially, the topology and surface composition of such materials play a key role in being promising materials in the anti-amyloidogenic interventions. Herein, 2nd and 3rd generation of pseudo-glycodendrimers (**PGDs**), based on hyperbranched polyester core with different sugar decorations, are synthesized, characterized, and used to investigate their anti-amyloidogenic properties toward Aβ (1–40) and (1–42), key players in Alzheimerś disease. The findings reveal that **PGDs** have a dendronized bottle brush architecture, as determined by SAXS analysis. **PGDs** are capable of interfering with the aggregation process of Amyloid-β peptides due to the high degree of sugar functionalization on the outer surface and the specific molecular shape. Additionally, cell viability studies indicate that **PGDs** exhibit concentration-dependent biocompatibility. Importantly, it is demonstrated that **PGDs** can be multi-functionalized by various sugar molecules, dyes, and/or peptides in a final one-pot approach. These findings suggest that **PGDs** may offer new avenues for therapeutic research in neurodegenerative diseases. Finally, it should be noted that this kind of highly branched glycopolymers possesses a molecular shape of dendronized bottle brushes and not a globular perfectly branched structure like glycodendrimers, as originally postulated.

## Highlights

• Improved synthesis of pseudo-glycodendrimers with higher degree of sugar functionalization.
• Sequential one-pot surface decoration for multifunctionalized pseudo-glycodendrimers
• Pseudo-glycodendrimers attributed to dendronized bottle brush as shape.
• Minor use of biocompatible pseudo-glycodendrimers as anti-amyloidogenic agent in ThT assay.
• Minor pseudo-glycodendrimer versus major polyamine-based glycodendrimer use in ThT assay.

## Graphical abstract

**Figure:**
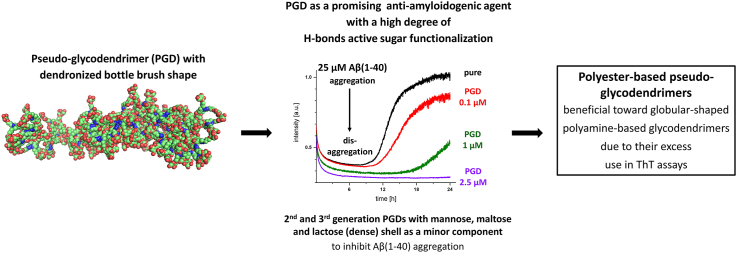

## 1 Introduction

Polysaccharides, glycopolymers and sugar-decorated nanostructures are promising materials in the field of biomedical, food and healthcare applications. The potential applications of these materials are extensive, encompassing areas such as from drug delivery systems to therapeutic agents for cancer therapy, neurodegenerative diseases, endocrine disorders, viral infections, prevention and remediation of cognitive impairment, and more [[1], [2], [3], [4], [5], [6]].

The diversity of sugar molecules and the abundance of useable basic structures enable the formation of an extensive array of sugar-decorated glycopolymer architectures (linear, star-shaped, dendritic, etc.). These sugar modifications are particularly intriguing due to their compatibility with numerous biological organisms, which can process mono-, oligo-, and polysaccharide units in varied ways: (i) utilization of sugars as an energy source; (ii) docking sugars onto surface receptors on cellular membranes; and (iii) incorporation into the extra- or intracellular matrix. In addition, modification of dendrimer surfaces with sugars facilitates the recognition of glycodendrimers as "self" by the organism, thereby reducing the immune response and preventing rapid clearance from the bloodstream [[7], [8], [9], [10]].

Therefore, the employment of sugar units for the embellishment of disparate polymer architectures (e.g., linear glycopolymers and glycodendrimers) is predicated on the effect of functional sugar crowding in the outer sphere. This underscores numerous enhancements in the domain of biomedical applications. These enhancements encompass increased biocompatibility, enhanced water solubility, reduced toxicity, targeted delivery, enhanced brain barrier permeation, anti-viral and anti-amyloidogenic properties, the prevention of cognitive impairment, and non-immunogenic properties, among others [1,[11], [12], [13], [14], [15], [16], [17], [18], [19]].

Neurodegenerative diseases like Parkinson, Lewy-Body, Creutzfeldt–Jakob or Alzheimers disease (AD) are in the focus of the medical investigations [[20], [21], [22], [23], [24], [25], [26]]. According to the World Health Organization (WHO), the global prevalence of dementia was estimated to be 55 million in 2019, with projections indicating a rise to 139 million by 2050 [27]. Of these, AD is the most prevalent form, accounting for approximately 62 % of all cases [20]. A range of polysaccharides, glycodendrimers (**GDs**) and pseudo-glycodendrimers (**PGDs)**, which possess hyperbranched core polymers with glycosylated dendrons of varying generations and a degree of branching next to 1 (≤100 %) are considered potential polymeric therapeutics specifically for the treatment of AD under *in-vitro* (e.g. anti-amyloidogenic agent) and *in-vivo* (animal study) conditions [13,[28], [29], [30], [31], [32], [33], [34], [35]]. For polysaccharides, there is a deep understanding of the biological action of inhibition and remediation of biological pathways in AD [[33], [34], [35]]. In addition, globular-shaped **GDs** exhibit first promising *in-vitro* and *in-vivo* therapeutic effects toward AD [13,28,31,32,36], but also toward prion and Creutzfeldt–Jakob disease [[37], [38], [39], [40], [41]].

In the aforementioned studies, generation-dependent dense-shell sugar decoration of poly(propyleneimine)-based dendrimers, with the potential for further surface functionalization with active units such as histidine, is a prerequisite for their efficacy as anti-amyloidogenic agent toward Aβ peptides (1–40, 1–42) [13,31,32] and prion peptides and proteins [[38], [39], [40], [41]]. The molar ratio between **GDs** and Aβ peptides plays a pivotal role in suppressing Aβ aggregation in the presence of excess **GDs**, while the use of minor **GDs** results in the acceleration of Aβ aggregation [31,32]. Furthermore, in (AD-based) animal studies [13,28,32,42] have demonstrated that **GDs** can traverse the blood-brain-barrier, preserve a protection of synapse integrity, and enhance memory function [13], while concomitantly reducing early-stage amyloid aggregates [28], without significantly impacting plaque formation [13,32]. There are two postulated working hypotheses from these studies as mid-term goal to improve the biodegradability of those promising **GDs** over time [43] and to reduce the excess ratio of **GDs** toward amyloidogenic peptides for the suppression of Aβ peptideś aggregation [31,32].

In order to enhance the efficacy of histidine-modified **GDs** in anti-amyloid interventions, it it is crucial to acknowledge the potential implications of the polyamino-based dendritic scaffold utilized in **GD**s. This scaffold may influence the toxicity profile over extended periods of observation and exhibit a diminished propensity for degradation within living systems [13,28,32]. In this regard, well-known polyester dendrimers [[44], [45], [46]] exhibit superior biocompatibility compared to poly(propyleneimine)-based **GDs** [43] and are also degradable into non-toxic substances [47]. The construction of these particular polyester dendrimers involves the incorporation of 2,2-bis(hydroxymethyl)propionic acid (bis-MPA), a versatile reagent employed in the multistep synthesis of dendrons, dendrimers, hyperbranched polymers, **PGDs**, and dendritic-linear hybrid structures [29,44,48].

In a recent study, the advanced interaction properties of bis-MPA-based polyester architectures toward Aβ peptides through fine-tuning of the macromolecular topology are shown [29], comparing mannose-decorated 4th to 6th generation polyester **GDs** and mannose-decorated 1st to 3rd generation polyester **PGDs** of similar diameters and similar numbers of mannose decoration. Mannose-decorated 2nd generation polyester **PGDs** exhibit the most promising anti-amyloidogenic activity within this study, while the 1st and 3rd generation of polyester **PGDs** outline a lower anti-amyloidogenic activity within the series of **PGDs**. Generally, **PGDs** with their hyperbranched polyester core structure outline better anti-amyloidogenic activity compared to the polyester **GDs**.

**Fig. 1:**
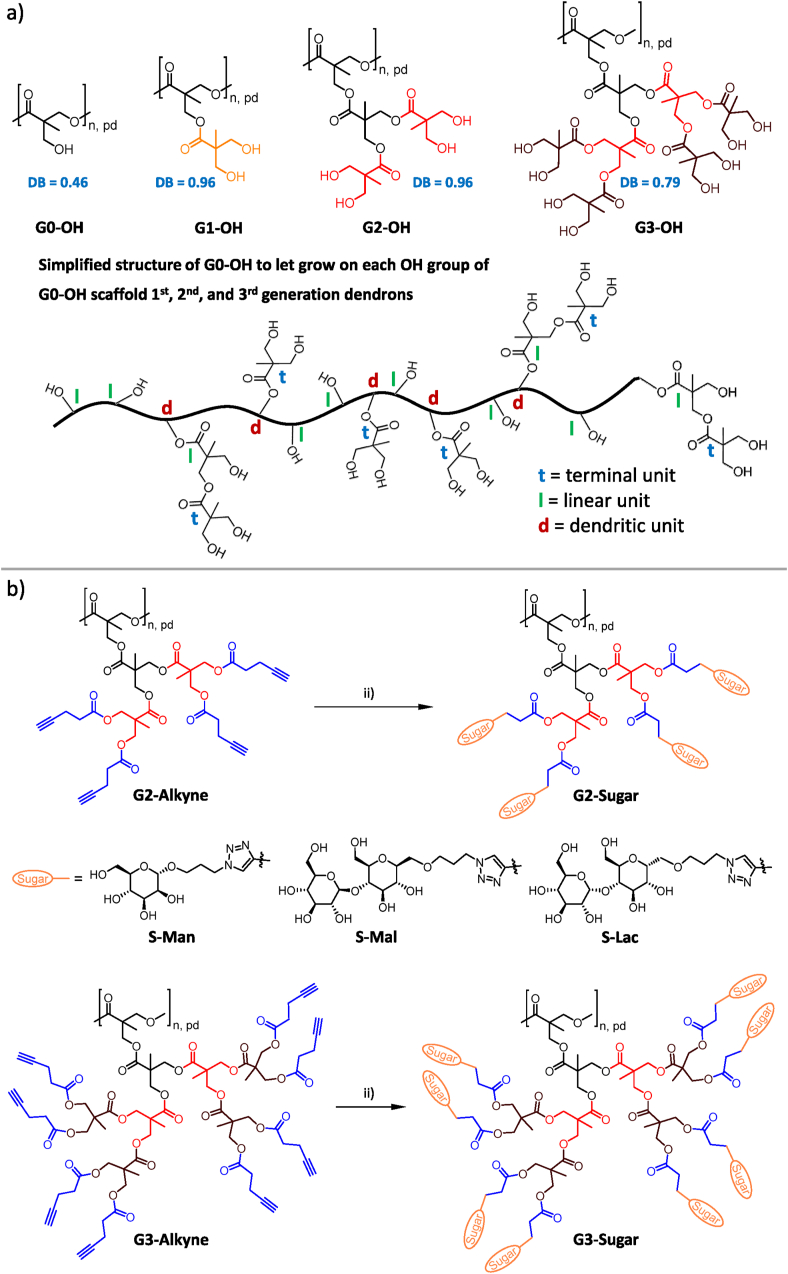
Synthesis of pseudo-dendrimers and pseudo-glycodendrimers based on the 1st, 2nd and 3rd generation dendrons and sugar functionalization of the hyperbranched core macromolecule **G0-OH**. A simplified structure of **G0-OH** is also shown that theoretically each OH group of **G0-OH** can be modified with the 1st, 2nd and 3rd generation dendrons. As a consequence, dendronization of **G0-OH** results in high degree of branching (DB) values for **G1-OH** and **G2-OH** besides a lower DB for **G3-OH** due to lower accessibility of OH groups in **G2-OH** for complete conversion of OH groups. DB values are placed next to the corresponding **PDs**, **G0-OH**-**G3-OH**. The term “pseudo” is directed to the use of a dispersed hyperbranched core macromolecule instead of using defined monodisperse core molecules, i.e. 2-, 3- or 4-functionalized core molecules, for the growth of dendrons to realize known dendrimer architectures. (a) Synthetic route from the basic structure **G0-OH** from an acid-catalyzed polycondensation from bis-MPA. i) First: 1.2 eq. DMAP, 1.2 eq. bis-MPA acetonide anhydride, pyridine, DCM, generation growth with protected bis-MPA-anhydride up to the third generation **G3-OH**. (b) The **GX-Alkyne** (X = 2 or 3) was obtained by conversion of **G2-OH** and **G3-OH** with 4-pentynic anhydride with DMAP in pyridine/DCM overnight at rt. Newly established click reaction for **PGDs** with copper(I)-species. Azido-functionalized mannose (S-Man), maltose (S-Mal), and lactose (S-Lac) were used in this step to synthesize 6 different **PGDs**. ii) 1.1 eq. azido-functionalized sugar, 0.1 eq. CuI, 0.1 eq. DIPEA, DMSO, 3 d, rt. (S = spacered sugar units).

The research conducted by Firdaus et al. [29] served as a catalyst for our decision to further expand the scope of our study on the biological interactions of **PGDs** with the key players, Aβ peptides (1–40, 1–42) (Fig. 5, Fig. 6, Fig. 8), in the context of AD. A central objective of this study is to validate the generation-dependent activity of **PGDs** in binding to and modulating the aggregation of Aβ peptides (1–40, 1–42). The sugar decoration (mannose, maltose, or lactose) of **PGDs** (Fig. 1), in conjugation with its dendritic scaffold, is hypothesized to impede the suppression of Aβ peptide aggregation in a heparin-free medium through the utilization of the Thioflavin T (ThT) assay (Figs. 5, **6** and **8**). The objective of this validation is to identify a preferential sugar activity in the presence of freshly prepared solutions of Aβ peptides (1–40) or Aβ peptides (1–42). It is hypothesized that **PGDs** may possess a sphere-like shape, which, similar to histidine-modified **GDs**, may offer beneficial molecular characteristics for biological studies [13,32,[38], [39], [40]]. To this end, the molecular shape was studied by SAXS (Fig. 3). Importantly, we wanted to study whether **PGDs** display also biological interactions toward Aβ peptide when used as minor component in ThT assay. This is in contrast to previously published anti-amyloidogenic characteristics of **GDs** and **PGDs** [13,29,32] where excess glycopolymers were used to inhibit amyloid aggregation in ThT assays. The cell viability study was used to validate the biocompatibility of **PGDs**, further supporting their potential in biomedical research.

## 2 Materials and methods

### 2.1 Materials

Sigma: dimethylol propionic acid (DMPA, ≥99 %), pyridine (anhydrous, 99.8 %), (+)-sodium-L-ascorbate (≥98 %), dichlormethylene (DCM, extra dry over molecular sieve, ≥99.8 %), 2,2-dimethoxypropane (DMP, 98 %), 3-bromo-1-propanol (97 %), acetone (analytic grade, 99.8 %), N,N′-dicyclohexylcarbodiimide (DCC, ≥99 %), lactoseoctaacetate (95 %), β-D-maltoseoctaacetate (95 %), α-D-mannoseoctaacetate (95 %), boron trifluoride etherate 48 % for synthesis, sodium methylate (25 % in methanol), copper iodide (98 %), sodiumazide (≥99.5 %), phosphate buffer saline (PBS, 1 mM). Alfa Aesar: 2,2 bis(hydroxymethyl) propionic acid (bis MPA, 98 %), 4-pentynoic acid (95 %). Merck: p-toluene sulfonic acid (pTsOH, 98 %). Iris Biotech: tri-Boc-azido-spermine (Spermine-(N3BBB), >98 %). MedChemExpress: Thioflavin T (98.45 %). Carl Roth: DOWEX 50W-X2 (100–200 mesh, H + Form), diisopropylethylamine (DIPEA, distillation and cover under argon with molecular sieve), Spectra Pore 7 dialysis membrane pre-treated RC tubing (2000 Da). Acros: methanol (99.9 % for analysis), AmberliteTM IR120 (H-Form), dimethyl sulfoxide (DMSO, extra dry over molecular sieve, 99.7 %), tetrahydrofuran (THF, 99,6 %, stabilized with butylhydroxytoluene). Gibco: phosphate buffer saline (PBS, 10 mM). Grüssing: copper(II)-sulfate pentahydrate (99 %). Fluoroprobe: AZ Dye 405 azide. JPT: TAT-azide, with the amino acid sequence: (5-azido-Pentanoyl)--Arg(Pbf)--Lys(boc)-Lys(boc)--Arg(Pbf)--Arg(Pbf)--Gln(Trt)--Arg(Pbf)--Arg(Pbf)--Arg(Pbf). NovoPep Limited: Aβ(1−40), with the amino acid sequence: [DAEFRHDSGYEVHHQKLVFFAEDVGSNKGAIIG LMVGGVV]. Water used for preparation of all solutions was obtained from a Milli-Q system (Millipore, Merck KGaA, Darmstadt, Germany).

Recombinant Aβ1-42 (M-DAEFRHDSGYEVHHQKLVFFAEDVGSNKGAIIGLMVGGVVIA), 20 μM in 20 mM phosphate buffer (pH 7.4), was generously provided by Prof. Sara Linse (Lund University). The recombinant Aβ1-42 was prepared and purified as previously described by Frankel et al. [49] and kept on ice before use.

For the study we used N2 cells made by Thinkararan et al. [50]. Cells were grown in conditioned media containing 47 % high glucose Dulbecco's modified Eagle's medium (DMEM) (# SH30243.01, GE Healthcare Life Sciences, Uppsala, Sweden), 47 % Optimem (#31985-047, ThermoFisher Scientific, Gothenburg, Sweden), 5 % fetal bovine serum (FBS) (# 10500-064, ThermoFisher Scientific, Gothenburg, Sweden), and 1 % penicillin/streptomycin at 37 °C in a humid 5 % CO2 incubator [51].

### 2.2 Methods

Methods are described in the Supporting Information (10.13039/100013443SI).

### 2.3 Synthetic processes

In this section it is only presented optimized experimental conditions for **G2-S-Man** and **G3-S-Man**, while all other syntheses of compounds are presented in the SI.

#### 2.3.1 Synthesis of pseudo-glycodendrimer G2-S-mannose (G2-S-Man)

The reaction was completely carried out in baked-out Schlenk tube under inert gas. G2-Alkyne (151.4 mg, 1.0 eq.) and α-D-mannose-propylazide (225.9 mg, 1.2 eq. calculated on the alkyne-groups of G2-Alkyne) were dissolved in dry DMSO (1.5 mL) under stirring. To the reaction mixture DIPEA (9.88 mg, 13.3 μL, 0.1 eq.) and copper(I)-iodide (16.3 mg, 0.1 eq.) were added. The reaction was stirred for 3 days at rt. After that, the mixture was quenched with water and purified by dialysis (2000 Da RC membrane) over 2 days, changing the water 3 times per day. The aqueous solution was freeze-dried to obtain the product, G2-S-Man, as a white powder (233.8 mg, yield: 63 %).

GPC: M_n_ = 74300 g/mol, Ð = 1.64, dn/dc = 0.085. ^1^H NMR (500 MHz, DMSO-*d*_6_): *δ* (ppm) = 0.93–1.33 (m, 3 H), 2.02 (br. s., 2 H), 2.63 (br. s., 2 H), 2.83 (br. s., 2 H), 3.39 (br. s, 1 H), 3.46 (br. s., 2 H), 3.62 (br. s., 3 H), 4.10 (br. s., 2 H), 4.35 (d, *J* = 17.65 Hz, 3 H), 4.50 (br. s., 1 H), 4.59 (br. s., 1 H), 4.65 (br. s., 2 H), 7.81 (br. s., 1 H).

#### 2.3.2 Synthesis of pseudo-glycodendrimer G3-S-mannose (G3-S-Man)

The reaction was completely carried out in baked-out Schlenk tube under inert gas. G3-alkyne (110.5 mg, 1.0 eq.) and α-D-mannose-propylazide (170.8 mg, 1.2 eq. calculated on the alkyne-groups of G3-alkyne) were dissolved in dry DMSO (1.5 mL dry) under stirring. To the reaction mixture DIPEA (6.92 mg, 9.3 μL, 0.1 eq.) and copper(i)-iodide (11.1 mg, 0.1 eq.) were added. The reaction was stirred for 3 days at rt. After that, the mixture was quenched with water and purified by dialysis (2000 Da RC membrane) over 2 days, changing the water 3 times per day. The aqueous solution was freeze-dried to obtain the product, G3-S-Man, as a white powder (139.3 mg, yield: 61 %).

GPC: M_n_ = 114900 g/mol, Ð = 1.37, dn/dc = 0.086. ^1^H NMR (500 MHz, DMSO-*d*_6_): *δ* (ppm) = 1.00–1.29 (m, 6 H), 2.02 (br. s., 5 H), 2.63 (br. s., 4 H), 2.82 (br. s., 4 H), 3.39 (br. s., 3 H), 3.46 (br. s., 5 H), 3.62 (br. s., 8 H), 4.10 (br. s., 5 H), 4.35 (d, *J* = 20.00 Hz, 8 H), 4.50 (br. s., 3 H), 4.59 (br. s., 3 H), 4.66 (d, *J* = 10.09 Hz, 5 H), 7.82 (br. s., 2 H).

## 3 Results and discussion

### 3.1 Synthesis of pseudodendrimers and pseudo-glycodendrimers

The synthesis of 1st, 2nd and/or 3rd generation of pseudo-dendrimers (**PDs**), **G1-OH**, **G2-OH**, **G3-OH**, **P-G2-Alkyne** and **P-G3-Alkyne** (Fig. 1), and 2nd and 3rd generation of **PGDs**, **G2-Sugar** and **G3-Sugar** (Fig. 1), based on still well-established synthetic procedures [29,48]. The most synthetic approaches were improved, including following issues: (i) An optimized working up process for hydroxy-functionalized **PDs**, **G1-OH**, **G2-OH**, and **G3-OH** (Fig. 1a); and (ii) an optimized click reaction for the conjugation of azidopropyl-functionalized mannose (S-Man), maltose (S-Mal), and lactose (S-Lac) moieties (Fig. S3) on **PD** surface of **G2-Alkyne** and **G3-Alkyne** to realize novel **PGDs**, **P-G2-S-Mal**, **P-G2-S-Lac**, **P-G3-S-Mal**, and **P-G2-S-Lac** (Fig. 1b), except previously published **G2-S-Man** and **G3-S-Man** (Fig. 1b) [29,48]. Further details on the improved synthetic approaches and a deeper discussion of the obtained results are presented in the SI.

Finally, these optimization steps provide a higher degree of functionalization (DoF) of sugar moieties on **PD** surface (Table 1; 94–97 %) which are superior compared with previously used reaction conditions [29] for the synthesis of **G2-S-Man** and **G3-S-Man** (Table S9) here in this study. A higher degree of sugar functionalization on **PGDs** surface is a prerequisite for conducting biological interaction studies, where the number of sugar units in the outer shell of various polymer architectures and the topology of glyco-architectures play key roles in biological interaction studies [13,29,31,32,[38], [39], [40], [41]].

**Table 1:** Degree of functionalization (DoF), molecular weight (M_w_, M_n_) and dispersity (Ð) for second and third generation pseudo-glycodendrimers.

Analysis	G2-S-Man	G2-S-Mal	G2-S-Lac	G3-S-Man	G3-S-Mal	G3-S-Lac
DoF [%]^a^	97	95	94	97	95	95
sugar units^b^	198	194	192	289	289	285
M_w_ [kg/mol]^a^	87.5	117.8	117.0	133.9	178.3	178.3

M_n_ [kg/mol]^c^	74.3	94.8	100.3	114.9	131.5	139.2
M_w_ [kg/mol]^c^	122.0	161.2	170.7	157.2	181.0	186.0
Ð (M_w_/M_n_)^c^	1.64	1.70	1.70	1.37	1.37	1.34

M_n_ [kg/mol]^c^ after 1 year	67.8	75.8	79.9	100.3	113.4	109.8
M_w_ [kg/mol]^c^ after 1 year	123.4	153.8	156.5	144.4	166.2	164.0
Ð (M_w_/M_n_)^c^ after 1 year	1.82	2.03	1.96	1.44	1.46	1.49

a Determination by ^1^H NMR spectroscopy.

b Determination by ^1^H NMR and SEC.

c Determination by SEC.

### 3.2 Multi-functionalized pseudo-glycodendrimers through sequential one-pot approach

**Fig. 2:**
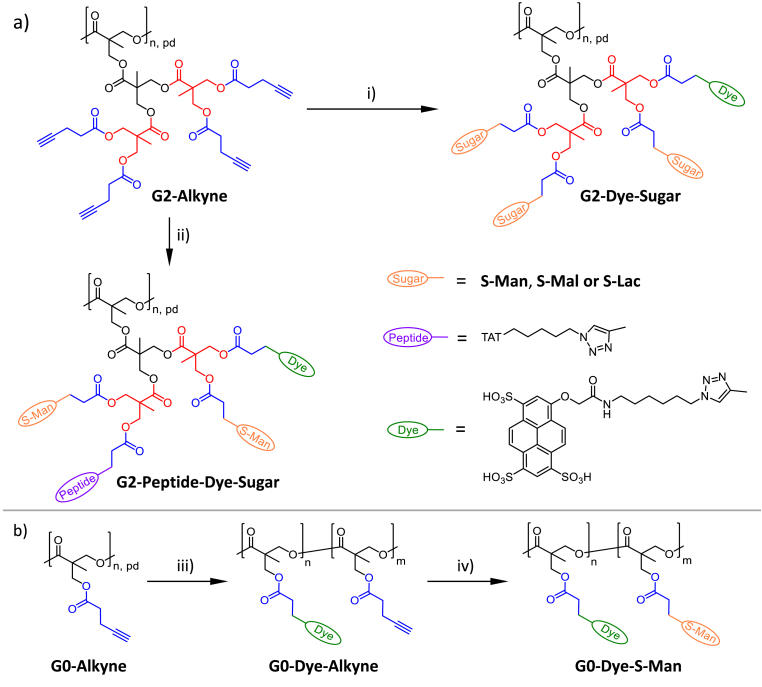
Synthesis of multifunctionalized pseudo-glycodendrimers, **G2-Dye-Sugar** (Figs. S42–S47) and **G2-Peptide-Dye-Sugar** (Fig. S50). a) Click reaction with up to three different functionalities in a one-pot reaction on the surface of the pseudo-dendrimer, **G2-Alkyne**: i) Addition of 2.0 eq. AZ-Dye 405 for 1 d, followed by addition of 245 eq. of spacered sugar (S-Man, S-Mal or S-Lac; 1.1 eq for each alkyne group) for 3 d, using 0.1 eq. CuI, 0.1 eq. DIPEA, DMSO, and rt, in total 4 d click reaction. ii) Addition of 2.0 eq. of AZ-Dye 405 for 1 d, then addition of 2.0 eq. of peptide (TAT-azide) for 1 d, and, finally, addition of 245 eq. of spacered sugar (S-Man; 1.1 eq for each alkyne group) for 3 d, using 0.1 eq. CuI, 0.1 eq. DIPEA, DMSO, rt, in total 5 d click reaction. b) Synthesis of dye-labeled core macromolecule **G0-Dye-Alkyne** and after 2 months of storage saturation conversion of **G0-Dye-Alkyne** with sugar units (**G0-Dye-S-Man**). iii) 2.0 eq. AZ-Dye 405, 0.1 eq. CuI, 0.1 eq. DIPEA, DMSO, 1 d, rt. iv) 48 eq. of spacered sugar (S-Man), 0.1 eq. CuI, 0.1 eq. DIPEA, DMSO, 3 d, rt.

The utilization of pure **PGDs** as an anti-amyloidogenic agent is a first trial in this study to validate a potential benchmark of such dendritic glycoarchitectures for future deeper biological studies. In addition to the sugar functionalization of **PDs**, **G2-Alkyne** and **G3-Alkyne**, the additional functionalization of **PDs** with dye molecules and peptides is of high interest to broaden the biological application of **PGDs** for future cellular tracking and location, but also (receptor-mediated) uptake and delivery processes. Thus, a sequential one-pot approach was established at which first the minor components (azido-functionalized dye and/or peptide molecules) are added to **PD** reaction solution for a 1 day click reaction, followed by the subsequent addition of excess sugar molecules (S-Man, S-Mal, or S-Lac) for 3 days of click reaction, thereby realizing multi-functionalized **PGDs**. Fig. 2 provides a summary of some examples of converted **G2-Alkyne** into **G2-Dye-Sugar** and **G2-Peptide-Sugar**, as well as the storage of **G0-Dye-Alkyne** for 2 months at 4 °C followed by their conversion of **S-Man** into **G0-Dye-Man**.

**Fig. 3:**
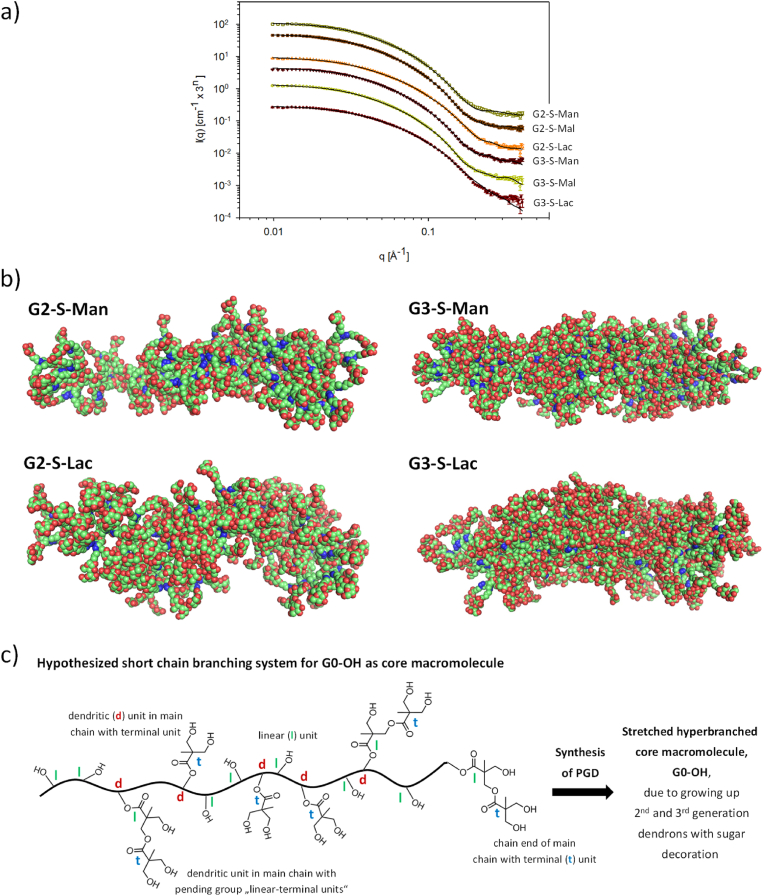
a) SAXS data for **G2-S-Man** (top), **G2-S-Mal**, **G3-S-Lac**, **G3-S-Man**, **G3-S-Mal**, and **G3-S-Lac** (bottom), (from bottom, shifted by 3^n−1^ to avoid overlap) and corresponding fits from the molecular-based modeling. b) Selected molecular models of **G2-S-Man**, **G2-S-Lac**, **G3-S-Man** and **G3-S-Lac** showing their dendronized bottle brushes. C) Hypothetical structure of the short chain branching system for the core macromolecule, **G0-OH**. Starting point for growing up 1st, 2nd and 3rd generation dendrons, including sugar decoration, along the short chain branching system for **G0-OH** to hypothetically induce the conformational transfer process for **G0-OH** from the coil-like state to the linearly stretched polymer chain of **G0-OH**.

Briefly, the applied sequential one-pot approach under the improved click reaction conditions at room temperature enables the synthesis of AZ-Dye 405-labeled **G2-Dye-sugar** and **G3-Dye-sugar**. A detailed characterization of AZ-Dye 405-labeled **PGDs** is presented later. Fig. S54 highlights also the great potential of the improved click reaction in comparison to the previous approach, which involved sequential one-pot modification with fluorescent dyes. The fluorescent intensity of **G2-Dye-S-Man** is notably higher, at 9 times the level observed for **G2-Dye-S-Man** prepared under the former reaction conditions [29]. The pure **G2-S-Lac**, devoid of any auto-fluorescent properties, serves as a reference. Due to the addition of only 2 dye molecules per **PDs** for the synthesis of dye-labeled **PGDs**, the ^1^H NMR signals for the dye substructure are not really visible in the ^1^H NMR spectra (Figs. S42–S47). After enlargement of the aromatic part of AZ-Dye 405 the corresponding aromatic signals can be assigned (Figs. S42–S47), while the protons of the aliphatic spacer between triazole and amide group are not assignable (Figs. S42–S47).

To show that the dye molecules are still conjugated to the surface of **PDs**, **G2-Alkyne** and **G3-Alkyne**, the dye-labeled core macromolecule, **G0-Dye**, is synthesized (Fig. 2b: step iii). Without the sugar molecules on the core macromolecule **G0-OH**, the aromatic and aliphatic ^1^H NMR signals of the dye for **G0-Dye-Alkyne** are assignable in ^1^H NMR spectrum (Fig. S31). The **G0-Dye-Alkyne** was stored in a freezer for two months and subsequently utilized in a second click reaction with S-Man to generate **G0-Dye-Man** (Fig. 2b: step iv). A DoF of 95 % is calculated by ^1^H NMR spectroscopy (Fig. S32). This result indicates that pre-functionalized **PDs** can be stored in an intermediate state and then undergo specific functionalization with other moieties and sugar molecules.

The concept of sequential one-pot approach is also applicable for triple click reactions wherein minor *N*-Boc protected spermine and AZ-Dye 405 are firstly conjugated and then excess S-Mal is deposited on the surface of **G2-Alkyne** (Fig. 2). The ^1^H NMR spectra of protected and unprotected **G2-Spermine-Dye-S-Mal** allow for the assignment of (part) structures (e.g. N-Boc groups at 1.35 ppm) of conjugated moieties (Figs. S48 and S49), while dye signals are only visible by enlargement with very low intensity. We can conclude that the click reaction is not only effective in binding sugar and dye to the surface of **PDs**, but also various oligoamine and peptide moieties (e.g. TAT functionalization of **PGDs**, **G2-TAT-Dye-S-Man**, Fig. S50). Consequently, the variety of possible outer shell functionalization of the pseudo-dendrimer system is therefore very broad.

### 3.3 Characterization of all pseudo-glycodendrimers

Hyperbranched polymers (**HP**), pseudodendrimers (**PDs**) and dendrimers exhibit distinctions in their structural and molecular parameters [44,[52], [53], [54], [55]]. Dendrimers are characterized as perfectly-branched polymers with a spherical, globular shape at higher generations and a theoretical dispersity Ð value of 1.0 [55]. In contrast, **PDs** possess hyperbranched polymers as core macromolecules from which perfectly-branched dendrons are grown, enabling the attainment of a greater number of functional groups and a higher molar mass in fewer steps compared to the dendrimer synthesis [29,52,53]. However, it should be noted that both **PDs**, in particular, **PGDs** synthesized in this study, exhibit larger Ð values, ranging from 1.34 to 1.72, as determined by SEC (Table 1 and S9). The most open concern of **PGDs** is the unknown molecular shape, which was hypothetically postulated to be sphere-/ellipsoid-like shape [29] prior to the commencement of the study.

#### 3.3.1 Determination of degree of branching, number of OH and sugar groups, and molecular weights of pseudo-dendrimers and pseudo-glycodendrimers

The ^1^H NMR spectroscopy and size exclusion chromatography (SEC, for samples with sufficient solubility) are the two primary analytical techniques used to characterize **PDs** and **PGDs**, respectively. ^1^H NMR spectroscopy allows for the signal assignment of the important structural part in each **PDs** and **PGDs** (Figures S9-S13, S15-S21, S23-S33, S35-S36, S38-S39, and S41); presented with signal assignments). These findings are partially supported by ^13^C 10.13039/501100004182NMR spectroscopy (Fig. S14, **S22**, **S34**, **S37**, and **S40**). The degree of branching (DB) was determined by the formula of Fréchet [56] via the determination of dendritic (d), linear (L) and terminal (t) units in the ^1^H NMR spectra of **G0-OH**, **G1-OH**, **G2-OH**, and **G3-OH** (Fig. S7, **S11**, **S15**, and **S18**). The DB (Table S5) increases from **G0-OH** (0.46) to **G1-OH** (0.96), while the DB decreases from **G2-OH** (0.96) to **G3-OH** (0.79) due to the increasing presence of imperfect at higher generations. However, ^1^H and ^13^C NMR spectra of **PGDs** (Fig. S30-**S50**) are not well-suited for the determination of DB due to broadening and/or overlapping signals for D, L, and T units.

The molecular weights of **G2-Sugar** and **G3-Sugar**, as determined by SEC and calculated from ^1^H NMR spectra, are presented in Table 1. The M_n_ values from SEC study range from 74 kg/mol to 139 kg/mol, while the calculated M_w_ values from ^1^H NMR lie in the range of 87 kg/mol to 178 kg/mol. The calculation of M_w_ by ^1^H NMR is detailed in the SI. The degree of functionalization of sugar moieties on **PGDs** surface can be calculated from ^1^H NMR spectra. The following parameters are necessary for the calculation of DoF: (i) the amount of converted yne groups (CH_2_-group at 2.36 ppm) and fabricated triazole rings (CH-group 7.8 at ppm) of **G2-Sugar** and **G3-Sugar** from ^1^H NMR spectra (Fig. S33, **S35**, **S36**, **S38**, **S39**, **S41**); and (ii) the molecular weights and the DB (Table S5) of **G2-OH** and **G3-OH**, allowing for the calculation of the number of OH groups in **G2-OH** (204 OH groups) and **G3-OH** (301 OH groups) (Table S7). This substantiates a high DoF for **G2-Sugar** and **G3-Sugar**, ranging from 94 % to 97 % (Table 1). For instance, **G2-S-Man** possesses 198 chemically conjugated S-Man moieties on the **PGDs** surface, while **G3-S-Man** possesses approximately 289 S-Man moieties. Further details concerning the DoF calculation are presented in the SI. The anchored chromophore groups (2–4) can be estimated on the **PGDs** surface by combining the molecular weights information determined and the fluorescence intensities using a calibration curve of free Az-Dye 405 and **G2-Dye-Sugar** and **G3-Dye-Sugar** (Fig. S52, **S55**). The results of this estimation are summarized in Table S10. This finding indicates that the dye molecules have been successfully attached to **PGDs** in sequential one-pot approach. Moreover, the dye-conjugated **PGDs**, **G2-Dye-Sugar** and **G3-Dye-Sugar** (Figs. S42–S47), exhibit similarly high values for DoF (≥ 95 %) and molecular weights (Table S10), as observed in the case of pure **PGDs** (Table 1).

The long-term stability of **PGDs** was investigated by SEC following a year of storage at −18 °C (Table 1). The results indicate a decrease in the molecular weight ranging from 10 % to 21 % for all **PGDs**. Concurrently, the corresponding Ð values also increase for all **PGDs**. For a polyester, it is well-established that the loss of molecular weight over this period is to be expected, given its known biodegradable nature. In summary, it can be concluded that the synthesized **PGDs** are attributed by a high degree of sugar functionalization, available for biological interactions as presented later in this study.

#### 3.3.2 Determination of molecular size and rho factor of pseudo-glycodendrimers

Dynamic light scattering (DLS) and small angle X-ray scattering (SAXS) are used to determine the hydrodynamic radius (R_h_), radius of gyration (R_g_), and rho factor (ρ = R_g_/R_h_) for the molecular shape/structure. Table 2 provides a summary of the values of R_h_, R_g_, and rho factor. The R_h_ values (3.25–3.75 nm) determined by DLS demonstrate minimal variation across the different samples in aqueous solution. The underlying model of DLS is a highly simplified representation of molecular shape, assuming spheres or sphere-like shapes. This “simplified molecular shape by DLS” is further verified by the determination of R_g_ values from SAXS measurements which are used to calculate the rho factor for **G2-Sugar** and **G3-Sugar**.

**Table 2:** Determination of rho factor for pseudo-glycodendrimer (**PGDs**) through SAXS study by model independent calculations and DLS study.

PGDs	SAXS *R*_*g*_*(nm)*	DLS *R*_*h*_*(nm)*	Rho *(R*_*g*_*/R*_*h*_*)*
**G2-S-Man**	4.03	3.25	1.23
**G2-S-Mal**	4.53	3.25	1.38
**G2-S-Lac**	4.36	3.25	1.35
**G3-S-Man**	4.29	3.75	1.14
**G3-S-Mal**	4.65	3.75	1.23
**G3-S-Lac**	4.65	3.25	1.41

The R_g_ values (4.0–4.6 nm) of **PGDs** are, on average, larger compared to the R_h_ values. The calculation of the rho factor primarily proves the presence of branched polymers (1.14–1.41; theoretical value of branched polymers: 1.23 [54]). The synthesized **PGDs** can be described as branched polymers in which the pending groups are more or less perfectly branched dendrons.

#### 3.3.3 Simplified linear molecular modelling of pseudo-glycodendrimers for fitting SAXS curves of pseudo-glycodendrimers

To obtain further structural information and visual impressions of the structure of our **PGDs**, a simplified molecular-based modeling is performed. In this modeling process, atomic resolution structures of the dendritic repeating units of the bis-MPA-based dendrons are linked linearly together by soft restraints. This approach should work reasonably well for lower generation of dendrons with smaller volume. The model is then optimized by random rotations and translations of the repeating units to fit the SAXS data. As illustrated in Fig. 3a, where the SAXS data is presented alongside model curves for **G2-S-Man**, **G2-S-Mal**, **G3-S-Lac, G3-S-Man**, **G3-S-Mal**, and **G3-S-Lac**. The model curves reproduce the data very well. The good agreement between the SAXS data and model curves suggests that dendronized bottle brushes serve as good models for the molecular shape of **PGDs**, under the assumption that the hyperbranched core macromolecule adopts a more linear configuration with the dendrons being attached, rather than a globular shape. This postulated molecular shape of **PGDs** is consistent with the calculated rho factor for branched polymers, as illustrated in Table 2. The visual representations of the model structures are shown in Fig. 3b and S62. Thus, the G2 and G3 structures exhibit a greater degree of compactness in models that contain bulky carbohydrate units in the outer part of the dendrons. Furthermore, a comparative analysis reveals that the G3 structure is more bulky than the G2 structure and is almost entirely covered by the carbohydrate units.

The structure of dendronized bottle brushes as the molecular shape for all **PGDs** is further supported by the consideration of the DB of 0.459 for the core macromolecule, **G0-OH** (Table S5 and S6). Theoretically, it is assumed that the core macromolecule, **G0-OH**, may preferentially possess a short chain branching system. This theoretical framework suggests a scenario in which dendritic units (20 %), saturated with terminal units (20 %) as a pending group, are preferentially integrated into the linear main chain. This theoretical simplification of the core macromolecule, **G0-OH**, with a value of DB below 0.5 is illustrated in Fig. 3c. It demonstrates a transition from a coil-like state of **G0-OH** to a linearly stretched **G0-OH** for the synthesized 2nd and 3rd generations of **PGDs** in solution. This ultimately results in the formation of worm-like structures for **PGDs**, resembling dendronized bottle brushes. Similar worm-like structures for glycosylated and PEGylated dendronized polymers with a higher polymerization degree for the main chain are still known from literature [[57], [58], [59]]. In contrast, the molecular architecture for **PGDs** exhibits very short stretched “main chains” (Table 2) for the dendronized core macromolecule, **G0-OH**.

The SAXS results, corroborated by the model of dendronized bottle brushes and the assumption of a hyperbranched core macromolecule, **G0-OH**, with longer linear sequences and short chain branching, have elucidated the molecular shape/architecture of **PGDs**. This molecular shape/architecture differs from the globular-shaped glycodendrimers as originally postulated. Consequently, the various **PGDs** macromolecules may manifest advanced interaction characteristics against biological (macro)molecules and systems.

### 3.4 Cell viability test by WST-1 assay

**Fig. 4:**
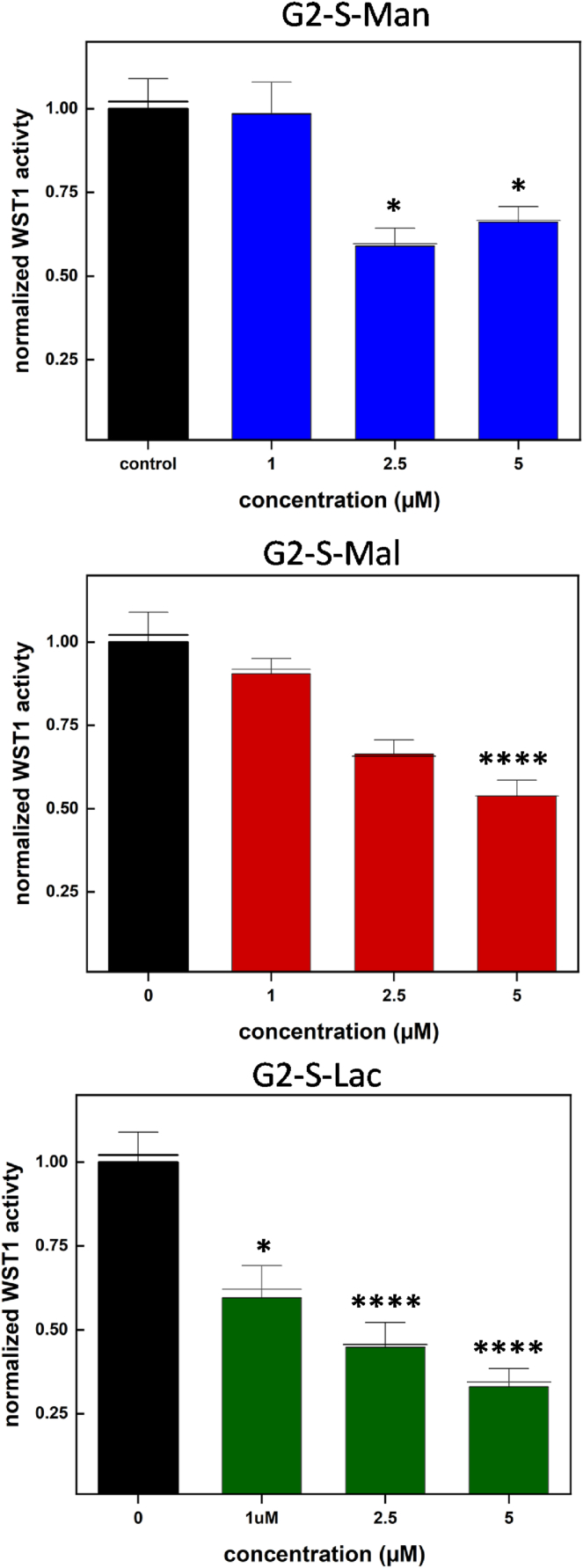
Effect of increasing concentrations of **PGDs** on mouse neuroblastoma (N2a) cell viability (∗∗∗∗*p* < 0.001) as measured by WST-1 assay after 48 h of incubation. ∗p < 0.0005, ∗∗∗∗p < 0.0001 when compared to the control (black) by one-way Anova.

Next, the metabolic activity of neuronal cells (N2a cells) was tested in the presence and absence of **PGDs**, **G2-S-Man**, **G2-S-Mal**, and **G2-S-Lac**. Fig. 4 summarizes the results of the concentration-dependent cytotoxicity of **PGDs** in complete cell culture media, using the water-soluble and cell-permeable tetrazolium salt reagent to test the metabolic activity of N2a cells.

**Fig. 5:**
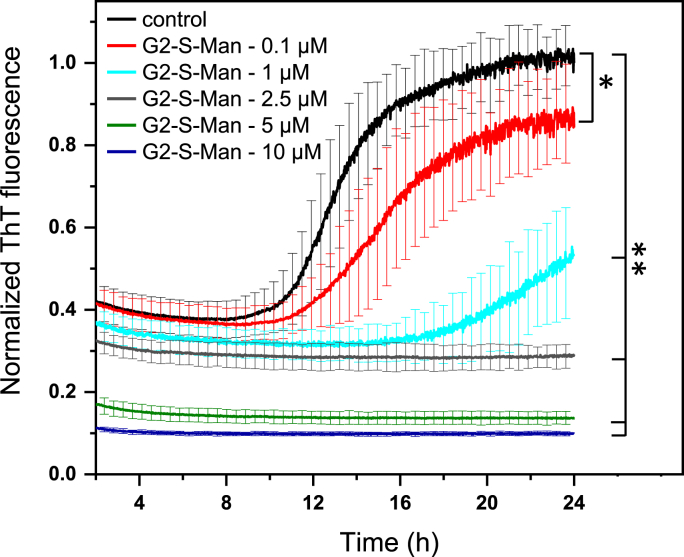
Fluorescence measurements of the ThT assay with 25 μM Aβ (1–40) in 1 mM PBS and **G2-S-Man** at different concentrations. At least seven (7–9) independent experiments were recorded for each concentration, using one batch of Aβ (1–40), and then an average value was calculated. Standard deviations are also presented. The fluorescence intensity of Aβ (1–40)/ThT after 24 h was normalized to 1 and used as a reference for all other results. Experiments based on the direct mixing of “freshly prepared Aβ (1–40) solution” with solutions of ThT at different **PGD** concentrations. Time period to show sigmoidal curve for Aβ (1–40) aggregation is presented between 2 and 24 h. ANOVA statistics: n = 9, ∗p = 0.0054, ∗∗p < 0.0001.

All 2nd generation **PGDs**, tested in this study, reduce cell viability at concentrations higher than 1 μM after 48 h. This is observed by a decrease in the absorbance of WST-1 assay, indicating reduced metabolic activity in treated cells. The reduction in cell viability becomes progressively more significant with increasing **PGD** concentrations, demonstrating a dose-dependent cytotoxic effect of **PGDs** on neuronal cells after 48 h. Therefore, for further series of **PGD** interaction experiments, we also considered 1 μM concentration for **PGDs** in ThT assay (Section 3.5) to investigate the biological interaction (=interference) properties of **PGDs** toward amyloids.

### 3.5 Interaction of pseudo-glycodendrimers with Aβ(1–40) and Aβ(1–42)

To investigate the biological properties of **PGDs**, particularly their interaction with amyloid peptides, the fibrillation of Aβ40 and Aβ42 is typically monitored in the presence of thioflavin T (ThT) fluorescence. ThT binds specifically to the β-sheet structures of amyloid fibrils through hydrophobic and π-π interactions, intercalating between the β-sheet layers [[60], [61], [62]]. Upon binding, the fluorescence of ThT increases significantly due to the restricted rotation of its aromatic rings, making it a reliable marker for the detection and quantification of amyloid fibril formation [63]. The ThT assay is a well-known method for assessing the ability of various (macro)molecules with different structures [13,32,64,65] and has been successfully used to investigate how various compounds interfere with amyloid aggregation [13,32,64,65].

This approach was applied to check the expected non-interfering properties of **PGDs** toward ThT (Figs. S61 and 8d), followed by the monitoring of the amyloid formation kinetics of Aβ(1–40) and Aβ(1–42) in the presence of **PGDs** to evaluate their interaction potential with Aβ peptides.

**Fig. 6:**
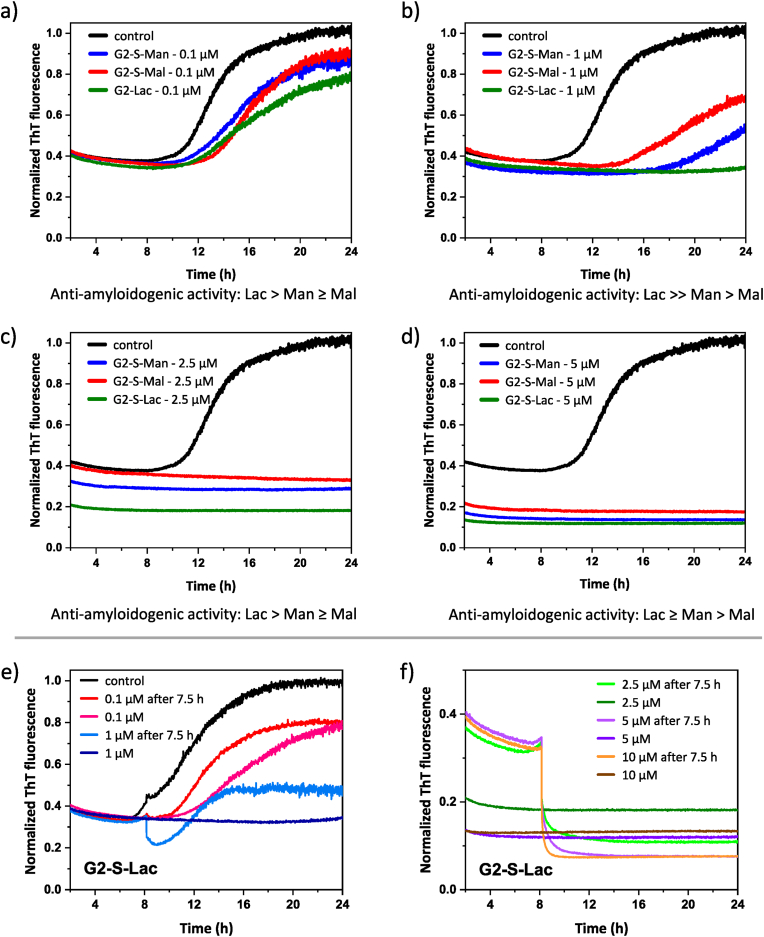
Study of Aβ(1–40) aggregation in the presence of different **PGDs**, using ThT assay: a) Comparison of different **G2-Sugar** at the same concentration of 0.1 μM. b) Comparison of different **G2-Sugar** at the same concentration of 1 μM. c) Comparison of different **G2-Sugar** at the same concentration of 2.5 μM. d) Comparison of different **G2-Sugar** at the same concentration of 5 μM. (a–d) At least seven independent experiments were recorded for each concentration, using one batch of Aβ (1–40), and then an average value was calculated. e) ThT assay with addition of **G2-S-Lac** after 7.5 h at 0.1 and 1 μM compared with the “freshly prepared Aβ(1–40) solution added to ThT + **PGD** solution” approach. f) ThT assay with addition of **G2-S-Lac** after 7.5 h at 2.5, 5 and 10 μM compared with the “freshly prepared Aβ(1–40) solution added to ThT + **PGD** solution” approach. (e–f) At least seven independent experiments were recorded for each concentration, using one batch of Aβ(1–40), and then the average value was calculated. The fluorescence intensity of pure Aβ (1–40) after 24 h was normalized to 1 and used as a reference for all other results. Experiments in a)-d) represent the “freshly prepared Aβ(1–40) solution” added to ThT + **PGD** solution” approach. Time period to show sigmoidal curve for Aβ (1–40) aggregation is presented between 2 and 24 h.

In the series of experiments with Aβ(1–40), it was particularly noteworthy to observe a decrease in ThT fluorescence when freshly prepared Aβ(1–40) is mixed with ThT and various concentrations of PGDs, especially during the lag phase. This phase (typically between 2 and 8 h, as shown in Fig. 5, Fig. 6), is characterized by a dynamic equilibrium between various states of at least low ThT-active Aβ(1–40) in solution.

In contrast, the control sample of Aβ(1–40) with ThT alone exhibits a relatively stable and elevated ThT fluorescence throughout the lag phase—higher than the corresponding fluorescence in samples where PGDs are present. This suggests that the PGDs can interfere with or suppress the ThT-active aggregation species during the early phase of aggregation (= lag phase). Such a dynamic equilibrium, where multiple aggregation states coexist, is still known from Aβ(1–42) during its lag phase, both in seeded and unseeded conditions [49,66]. In those systems, polymorphic Aβ(1–42) species are present, though their exact distribution remains undefined. By analogy, the results for (pure) Aβ(1–40) samples (Fig. 5, Fig. 6) suggest that during the lag phase, low ThT-active aggregates appear to persist for several hours before entering the elongation phase, leading to the sigmoidal aggregation profile characteristic of Aβ(1–40) fibrillization.

#### 3.5.1 Low complexation capacity of PGDs toward ThT

The study of **PGDs** as an anti-amyloidogenic agent provides that **PGDs** have a low complexation capacity toward the dye ThT. Fig. S61 highlights this observation that the ThT fluorescence is only 3 % in the presence of **PGD**, **G2-S-Man**, in contrast to the presence of Aβ(1–40) with 100 % for fluorescent ThT in 1 mM PBS. A similar scenario is also given for the ThT fluorescene (100 %) in the presence of Aβ(1–42) compared to the much lower ThT fluorescence in the presence of the pure **PGDs**, **G2-S-Man**, **G2-S-Mal**, and **G2-S-Lac** (Fig. 8d). This implies that **PGDs** have a very low potential to complex ThT in solution to provide the desired hydrophobic environment to enhance the fluorescent properties of ThT. Thus, due to the low complexation capacity of **PGDs** toward ThT, the fluorescent intercalation of ThT in the amyloid fibrils for Aβ(1–40) and Aβ(1–42) will not be suppressed or disturbed.

#### 3.5.2 Aβ(1–40) aggregation in presence of pseudo-glycodendrimers

To study of Aβ(1–40) aggregation, 2nd generation **PGDs**, **G2-S-Man**, **G2-S-Mal**, and **G2-S-Lac**, were used with molar ratios of 1:250 (25 μM:0.1 μM), 1:25 (25 μM:1 μM), 1:10 (25 μM:2.5 μM), 1:5 (25 μM:5 μM), and 1:2.5 (25 μM:10 μM), with **PGDs** as a minor component as anti-amyloidogenic agent. Fig. 5 shows the concentration and time dependent inhibition of **G2-S-Man** against Aβ(1–40) aggregation. This series of experiments shows the influence of **PGDs** on immediately mixed Aβ(1–40) solution (= freshly prepared Aβ(1–40) solution) with a mixture of **PGDs** and ThT. Pure Aβ(1–40) aggregation in the presence of ThT is used as a control.

The control experiment of Aβ(1–40) in the presence of ThT shows the typical sigmoid curve for nucleation polymerization (Fig. 5) as known from amyloidogenic peptides [37,66]. This shows the expected lag phase (2–8h) for the nucleation of Aβ(1–40) in solution with a stable ThT fluorescence, followed by the elongation phase (e.g. faster fibrillar β-sheet formation; 8–22 h) and, finally, the plateau phase (= formation of amyloid fibrils; 22–24 h briefly indicated). The metastable lag phase of pure Aβ(1–40) indicates the presence of low ThT-active forms in the solution.

**G2-S-Man** at a concentration of 1 μM can significantly interfere with Aβ(1–40) aggregation by reducing ThT intensity (= longer lag phase and shorter elongation phase) compared to that of the control of pure Aβ(1–40) (25 μM). The higher **G2-S-Man** concentrations cause a longer lag phase, which finally results in no plateau in the ThT kinetics after 24 h. This indicates that no intense Aβ fibril formation occurs under the selected conditions (1 mM PBS; absence of heparin; pH 7) in the ThT assay. Furthermore, the increasing **PGD** concentrations induce a continuous decrease of ThT fluorescence in the lag phase (2–8h) of low ThT-active forms of Aβ(1–40) in the solution. This indicates that **G2-S-Man** is capable of immediately forming non-covalent interactions with any type of soluble aggregates of Aβ(1–40). This leads to a concentration-dependent reduction of aggregated solution states of Aβ(1–40). Repeat experiments (at least n = 7) with the applied molar ratios confirm the high potential of **G2-S-Man** as a minor agent that can interfere with amyloid formation (Fig. 5).

Based on our previous results demonstrating the role of H-bond-active sugar shell interactions in amyloid formation [13,29,31,40], the comparison of interference properties of all 2nd generation **PGDs**, **G2-S-Man**, **G2-S-Mal**, and **G2-S-Lac**, against soluble and stable Aβ(1–40) aggregates from freshly prepared Aβ(1–40) is shown in Fig. 6a–d, using molar ratios of 1:250 (25 μM:0.1 μM), 1:25 (25 μM:1 μM), 1:10 (25 μM:2.5 μM), 1:5 (25 μM:5 μM) to monitor ThT kinetics during the fibril formation of Aβ(1–40). The results show that only **G2-S-Lac** at 1 μM completely inhibits Aβ(1–40) aggregation, while **G2-S-Mal** has the lowest potential to suppress Aβ(1–40) aggregation (Fig. 6b) with a time-dependent delay of 25 μM Aβ(1–40) aggregation after 14.4 h. The different anti-amyloidogenic properties of **G2-S-Mal** and **G2-S-Lac** can be attributed to the different structural compositions of the disaccharides on the **PGD** surface.

No relevant amounts of Aβ aggregates are formed in the ThT assays with 2.5 μM (molar ratio 1:10) and 5 μM (molar ratio 1:5) of all **PGDs**. The results indicate that at a molar ratio of 1:10 the minor **PGDs** are effective enough to suppress the formation of Aβ fibrils. The fluorescence intensity of ThT (Fig. 6c and d) decreases from the starting point and remains constant over time, resulting in a steady lag phase of soluble Aβ(1–40) states. This clearly implies that only low ThT-active Aβ species are formed during the dissolution process (mixing freshly prepared Aβ solution with solutions of **PGDs** and ThT), are also destroyed by **G2-S-Mal** and **G2-S-Lac** compared to the results of **G2-S-Man**. Moreover, it can be concluded that **G2-S-Lac** has the highest potential as an anti-amyloidogenic agent compared to that of **G2-S-Man** and **G2-S-Mal**, whereas **G2-S-Mal** has the lowest potential of the 2nd generation **PGDs** (Fig. 6a–d). The 3rd generation **PGDs**, **G3-S-Man**, **G3-S-Mal**, and **G3-S-Lac**, also outline superior anti-amyloidogenic properties as a minor component toward Aβ(1–40) solutions (Fig. S60). A general trend can be observed: the higher the concentration of PGDs as a minor component relative to Aβ(1–40), the lower the ThT fluorescence intensity. This reduction corresponds to a decreased presence of ThT-active Aβ(1–40) species as shown in Figs. 5, **6**, and **S60**. This suggests that the surface-attached sugar units on **PGDs** act as key surface-active moieties, facilitating various non-covalent interactions with Aβ(1–40) species and thereby influencing their aggregation state (Table 3). Further mechanistic insights and experimental details are provided in the following sections.

**Table 3:** Determination of surface-active sugar units per **PGD** in ThT assay to minimize and to inhibit fibril formation, using 0.1, 1, 2.5, 5, and 10 μM of **PGD** toward 25 μM of **Aβ(1**–**40)**, including the reduction of low ThT-active forms of **Aβ(1**–**40)** in the lag phase (Figs. 5, 6, and S60).

	G2-S-Man	G2-S-Mal	G2-S-Lac	G3-S-Man	G3-S-Mal	G3-S-Lac
Sugar units per PGD	198	194	192	289	289	285
[PGD] in ThT assay	Active sugar units per PGD in ThT assay					
0.1 μM	19.8 μM	19.4 μM	19.2 μM	28.9 μM	28.9 μM	28.5 μM
1 μM	198 μM	194 μM	192 μM	289 μM	289 μM	285 μM
2.5 μM	495 μM	485 μM	480 μM	722.5 μM	722.5 μM	712.5 μM
5.0 μM^a^	980 μM	970 μM	960 μM	1445 μM	1445 μM	1425 μM
10.0 μM^b^	1960 μM	1940 μM	1,920 μM	2890 μM	2890 μM	2850 μM

^a,b^ Active sugar units per **PGD** toward 5 μM of Aβ(1–42) in ThT assay.

Knowing that **G2-S-Lac** demonstrates the strongest interference with Aβ(1–40) formation (Fig. 6a-**6d**), it was selected for further investigation of its interaction with soluble aggregation states of Aβ in the lag phase (2–8h). In this series of experiment, **PGDs** are added approximately 7.5–8 h after mixing the ThT dye with pure Aβ(1–40), before the time point (= elongation phase after 8 h) starts when Aβ(1–40) aggregation begins. In general, the results show that **G2-S-Lac** stabilizes and interacts with both, (i) directly mixed with Aβ(1–40) solution from the beginning of the lag phase) and (ii) soluble aggregation states of Aβ(1–40) at the end of the lag phase (Fig. 6e and f). Due to the presence of slightly elevated NaCl levels in the 1 mM PBS solution (see 10.13039/100013443SI), the pure Aβ(1–40) sample incubated with ThT contains a mixture that includes low ThT-active Aβ(1–40) species, as supported by analogous observations for Aβ(1–42) in its lag phase [66]. The comparative analysis of lag-phase behavior provides additional insight into the molecular interaction between **G2-S-Lac** and Aβ(1–40). Specifically, the surface-active lactose units on **G2-S-Lac** (Table 3) appear to reduce the abundance of metastable, ThT-detectable Aβ(1–40) species, likely through non-covalent interactions that interfere with early-stage aggregation (Fig. 6f).

In detail Fig. 6e compares the ThT kinetics of Aβ(1–40) at different time points in the lag phase using 0.1 and 1 μM of **G2-S-Lac**. For the addition of the lowest concentration of **G2-S-Lac** there is a faster increase in Aβ(1–40) aggregation for the aggregated Aβ(1–40) solution states, but with the same end point for ThT fluorescence compared to that of Aβ(1–40) aggregation with the immediate mixing of Aβ(1–40) solution with “**G2-S-Lac** + ThT” solution. The concentration of 1 μM shows a significant difference in the addition of **G2-S-Lac** after 7.5 h. Finally, there is still a delayed aggregation of the soluble aggregated Aβ(1–40) solution states in the lag phase, but with lower fluorescence intensity compared to the control. In contrast a stabilized Aβ(1–40) aggregation with constant ThT fluorescence is observed, when Aβ(1–40) is added immediately to the “**G2-S-Lac** + ThT” solution at 1 μM for **G2-S-Lac**. At higher concentrations of 2.5, 5, and 10 μM for **G2-S-Lac**, a different trend is observed (Fig. 6f). The addition of **G2-S-Lac** after 7.5 h leads to a decrease in the fluorescence of ThT for the already formed “soluble aggregated Aβ(1–40) solution states in 1 mM PBS at pH 7.4 in the lag phase”, and finally a very low ThT fluorescence is observed, as found in the case of complete inhibition of soluble Aβ(1–40) aggregates after the direct mixing of Aβ(1–40) solution with “**G2-S-Lac** + ThT” solution. This also indicates that molar ratios of up to 1:10, using **G2-S-Lac** as a minor component compared to Aβ(1–40), are able to inhibit the further aggregation of soluble aggregated Aβ(1–40) solution states and even result in the disaggregation of soluble aggregates of Aβ(1–40). We can hypothesize that at least **G2-S-Lac** is a suited anti-amyloidogenic agent for the inhibition of Aβ(1–40) and smaller Aβ peptides in solution, partially independent of the solution state of Aβ entities. Overall, we can state that **PGDs**, especially **G2-Sugar**, outline promising biological interfering properties in pure Aβ solutions to act as efficient anti-amyloidogenic agents with molar ratios of 1:10 and lower being the most suitable.

**Fig. 7:**
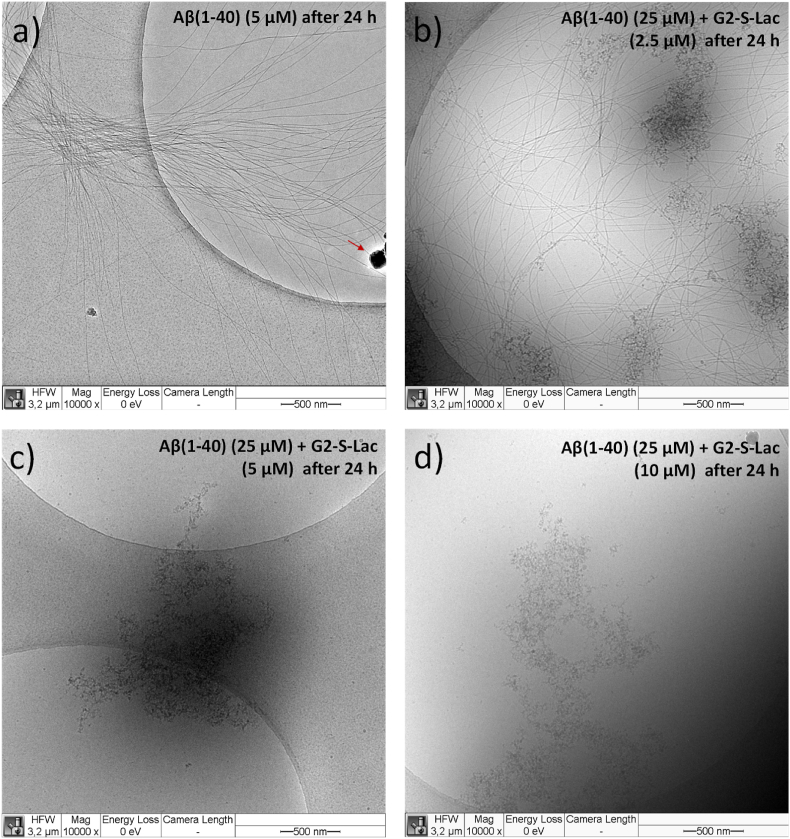
Cryo-TEM study of Aβ(1–40) in the presence and absence of **G2-S-Lac** to show fibril formation of Aβ(1–40) (a), partially **G2-S-Lac** decorated Aβ(1–40) (b), or aggregated biohybrid structures, composed of Aβ(1–40) and **G2-S-Lac** with undefined shapes (b–d). The ThT assay conditions are used, where samples are cryo-frozen after 24 h. In the case of pure Aβ(1–40) a less concentrated solution is used due to excessive fibril formation of Aβ(1–40). The red arrow in (A) indicates the presence of ice particles. The diameters of individual fibrils are in the range of 10 ± 1 nm (Fig. S69). Additional cryo-TEM images for frozen states of Aβ(1–40) in the presence and absence of G2-S-Lac are shown in Figs. S63–S68. The cryo-TEM images shown are representative of n similar images from different spots of TEM grids. Consistent Aβ(1–40) fibrils or aggregated biohybrid structures can be found on the different spots of TEM grid.

To verify the ThT assay results for the disaggregation process of Aβ(1–40) in the presence of different concentrated **G2-S-Lac** solutions, a cryo-TEM study was carried out under the conditions to simulate the final solution state of Aβ(1–40) in the ThT assay after 24 h. Fig. 7 and **S63-S68** highlight the frozen states of Aβ(1–40) in the presence of **G2-S-Lac**. Briefly, fibril formation of Aβ(1–40) is visible only at 2.5 μM of **G2-S-Lac** (Fig. 7b) next to the pure Aβ(1–40) solution (Fig. 7a). This undoubtedly indicates that the lower ThT fluorescence of Aβ(1–40) (25 μM) with **G2-S-Lac** (2.5 μM) (Fig. 6c) can be attributed to (partially) **G2-S-Mal** stabilized Aβ(1–40) fibrils besides the formation of undefined aggregates composed of Aβ(1–40) and **G2-S-Lac** (Fig. 7b). Only undefined aggregates composed of Aβ(1–40) and **G2-S-Lac**, but no fibrils are attributable and visible at 5 and 10 μM after 24 h (Fig. 7c and d, for further insights see Figs. S67–S68), leading to the lowest ThT fluorescence in ThT assays within the experimental series (Fig. 6d and f). Thus H-bonding interactions of **PGDs** can be postulated as the main-driving force of the interactions between Aβ(1–40) and **PGDs** due to the high number of H-bond-active sugar units in the outer shell of **PGDs**. This emphasizes the simultaneous non-covalent interactions of Aβ(1–40) molecules with the huge sugar surface of **PGDs**, probably, leading to the cooperative effect of the sugar shell. We assume that single or multiple sugar unit(s) are involved in the surface interactions with individual Aβ(1–40) molecules. Such interaction characteristics are described in a more simplified way in a molecular modeling study of Aβ(1–40) molecules with densely packed sugar balls [36]. Furthermore it is conceivable that Aβ(1–40) molecules are located or trapped between the intramolecular space of at least two sugar molecules within the sugar shell. This could lead to a potential crowding effect in the vicinity of sugar units due to other non-covalent interactions other than H-bonds. These distinct surface-driven interactions between Aβ(1–40) molecules and the **PGD** surface are responsible for the suppression of fibril formation in ThT assays, as exemplified in the cryo-TEM study between Aβ(1–40) and **G2-S-Lac**. This further underlines the huge influence of sugar units as surface-active units in **PGD** macromolecules as anti-amyloidogenic agent, where we can smoothly consider the molar influence of surface-active sugar in PGD in ThT assays as well. This consideration is highlighted in Table 3.

**Fig. 8:**
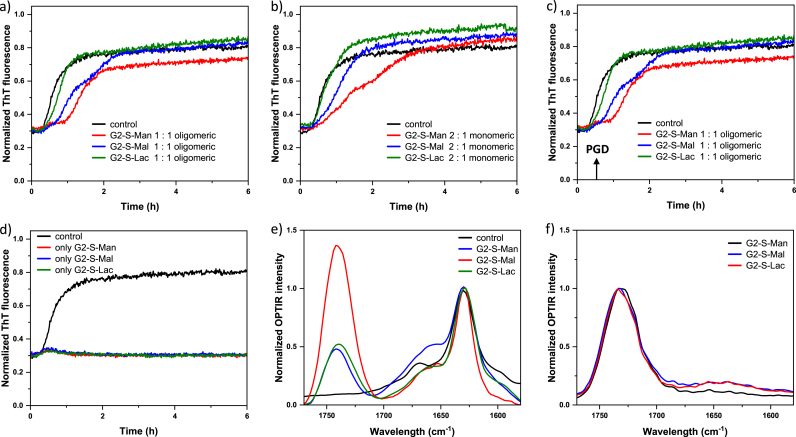
Effect of 2nd generation **PGDs** on the aggregation kinetics of Aβ(1–42). Fibril formation was measured by monitoring ThT fluorescence variation up to 6 h (Normalization process refers to fibril formation up to 14 h for control (pure Aβ(1–42) set as 1 to normalize all other data, obtained from samples after 14 h), and structural changes were measured by OPTIR. a) Effect of **G2-S-Man**, **G2-S-Mal**, and **G2-S-Lac**, on aggregation of Aβ(1–42), 1 to 1 ratio (5 μM of Aβ (1–42) and 5 μM of **PGDs** in 1 mM PBS at pH 7.5). b) Effect of **G2-S-Man**, **G2-S-Mal**, and **G2-S-Lac** on aggregation of Aβ(1–42), 1 to 2 ratio (5 μM of Aβ (1–42) and 10 μM of **PGDs** in 1 mM PBS at pH 7.5). c) Effect of **G2-S-Man**, **G2-S-Mal**, and **G2-S-Lac**, on the aggregation of Aβ(1–42) (5 μM of Aβ (1–42) and 10 μM of **PGDs** in 1 mM PBS at pH 7.5), when **PGDs** are added after 30 min of aggregation. d) **PGDs** alone do not bind ThT. e) Binding of **G2-S-Man**, **G2-S-Mal**, and **G2-S-Lac** to Aβ(1–42) as indicated by the changes in intensity of the peak centered at 1725 cm^−1^ as shown by the normalized OPTIR intensity to the peak 1625 cm^−1^ corresponding to β-sheets. f) OPTIR intensity of the ester band at 1725 cm^−1^ for **G2-S-Man**, **G2-S-Mal**, and **G2-S-Lac.** The averaged data (n = 3) without standard deviations are shown for (a–c).

#### 3.5.3 Aβ(1–42) aggregation in presence of pseudo-glycodendrimers

To investigate the interaction of **PGDs** with recombinant 5 μM Aβ(1–42), which generally has a higher hydrophobicity than Aβ(1–40) (chapter 3.7.1), its fibrillation in the presence of **PGDs** was studied using ThT fluorescence and the results are presented in Fig. 8. Very slight changes in ThT fluorescence upon the addition of **PGDs** are observed in the ThT kinetics, including the lag phase, for 2nd generation **PGDs**, **G2-S-Man**, **G2-S-Mal**, and **G2-S-Lac** (Fig. 8a). This indicates that at the 1:1 ratio, **PGDs** do not noticeably interfere with Aβ(1–42) aggregation. On the other hand analysis of infrared spectra, obtained from the end product of Aβ(1–42) kinetics, shows that all 2nd generation **PDGs** bind to fibrils to a different extent and thus influence the molecular packing of Aβ(1–42) (Fig. 8e and f).

This result is unexpected compared to the strong inhibitory effects of **PGDs** on Aβ(1–40) aggregation (Fig. 5, Fig. 6). The lack of effect on Aβ(1–42) suggests that Aβ(1–42) aggregates much more rapidly than Aβ(1–40). In addition, Aβ(1–42) can form a more stable β-sheet structure, making it less susceptible to **PGD** interferences.

However, with increasing **PGD** concentrations, particularly when **PGDs** are added in a 1:2 ratio as an excess component, some effects on ThT kinetics can be observed, especially in the case of **G2-S-Man**. The biological interaction of 2nd generation **PGDs**, **G2-S-Man**, **G2-S-Mal**, and **G2-S-Lac,** with intermediate products of Aβ(1–42) aggregation, is presented in Fig. 8c. This interaction profile of **PGDs** can only be observed, when **PGDs** are added after 30 min of Aβ(1–42) aggregation started (Fig. 8c). Fig. 8c shows that **PGD**, **G2-S-Man**, more effectively interfere with the kinetic intermediates rather than with Aβ(1–42) (Fig. 8b). These results indicate that 2nd generation **PGDs** have a greater affinity to interact with intermediate forms of Aβ(1–42) during the aggregation.

## 4 Conclusion

In comparison with previous studies [29,48], we demonstrate an improved synthetic approach for **PDs** and **PGDs** (Fig. 1, Fig. 2) that enables the fabrication of dendritic poly(bis-MPA-ester) scaffolds with a high degree of surface functionalization (≥94 %) for various sugar molecules (Man [29], Mal, and Lac). The efficient click reaction for the final surface decoration of **PDs** into **PGDs** permits the sequential decoration of minor surface moieties of dye, oligoamine, and/or peptides besides the major moieties of sugar molecules in a one-pot reaction under very mild reaction conditions (shortened reaction time at room temperature) (Fig. 1, Fig. 2). The 2nd and 3rd generations of **PGDs** manifest the molecular shape of dendronized bottle brushes, as determined by SAXS analysis. The hyperbranched core macromolecules (DB = 0.46), **G0-OH**, adopt a structure with linear sequences and some short chain branching, bearing pending groups of dendrons with varying degrees of perfection and sugar molecules. This sugar shell of **PGDs** is responsible for the **PGD** solubility in aqueous, buffer and cell media solutions. Thus, the hypothesized sphere-like shape for **PGDs**, initially postulated in the study, is not confirmed by the analysis of the SAXS data. The rho factor determination for all **PGDs** further underscores the state of branched macromolecules and refutes the hypothesis of a sphere-like shape, thereby corroborating the postulated molecular shape of dendronized bottle brushes as determined by the SAXS study (Fig. 3). Furthermore, molecular modeling of **PGDs**, using a model of linearly polymerized sugar-decorated 2nd and 3rd generation bis-MPA-ester-based dendrons, corroborates the hypothesis that **PGDs** do not adopt a sphere-like shape. In summary, the suggested molecular shape of **PGDs** is attributable to the utilization of a stretched hyperbranched core macromolecule, **G0-OH**. It is assumed that this core macromolecule possesses integrated dendritic units, with one terminal unit as a pending group, within the linear chain of **G0-OH**, exhibiting a DB of 0.46 (Fig. 3c).

In addition, the findings of this study demonstrate the capacity of 2nd generation **PGDs** to impede the fibril formation of Aβ(1–40) and to interact with Aβ(1–42) fibril formation, as determined by ThT assay (Fig. 5, Fig. 6, Fig. 8). This indicates that the molecular shape of dendronized bottle brushes for 2nd generation **PGDs** plays a crucial role in its very specific interactions with Aβ(1–40), where **PGDs** as a minor component are implicated in the suppression of amyloid aggregation. The suppression mechanism of fibril formation of Aβ(1–40) by **PGDs** is attributed by a hypothetically complex relationship of surface interactions through a combination of H-bonds and cooperative and crowding effect of sugar units toward Aβ(1–40) molecules, leading to multiple non-covalent interactions between **PGDs** and Aβ(1–40) molecules. Furthermore, cryo-TEM results indicate that fibril structures of Aβ(1–40) are not visible and suggest the formation of typically aggregated biohybrid structures composed of minor 2nd **PGDs** (5 and 10 μM) and excess Aβ(1–40). The biological interactions of **PGDs** as a minor component are particularly promising when compared to that of previously used sphere-like polyamine-based glycodendrimers, which were always used in excess toward Aβ peptides in ThT assays [13,31,32]. Recently published **PGDs**, for example **G2-S-Man**, exhibiting a generally lower degree of surface Man functionalization [29], the optimized **G2-S-Man** here in this study outlines the desired beneficial interfering properties as a minor component in the ThT assay. Generally, it is noteworthy that in the ThT assays 2nd and 3rd generation **PGDs** are immediately able to interact with any soluble kinds of organized Aβ(1–40) molecules during the lag phase. This interaction profile of **PGDs** exhibits considerable promise in facilitating the early recognition of solution structures of Aβ(1–40), thereby paving the way for the future development of anti-amyloidogenic agents in the context of Alzheimer's disease (AD).

In contrast, 2nd generation **PGDs** exhibit random and partial interactions with different solution states of the highly hydrophobic Aβ(1–42) under ThT assay conditions. IR-analyzed samples of **PGD**/Aβ(1–42) aggregates demonstrate a shift in the molecular packaging of Aβ(1–42). In addition, 2nd generation **PGDs** are able to interfere with the more stable β-sheet structure of aggregated Aβ(1–42) for a limited time period. Further improvements in **PGD** structures may be achieved by exchanging triazole rings for sulfide linker units, which serve as linker between the dendritic scaffold and sugar units. This modification has the potential to eliminate the presence of cationic units in the outer shell of **PGDs**, thereby enhancing its biocompatibility with various (neuronal) cells in subsequent studies.

Overall, the specific shape and the substantial density of surface sugar functionalization in the dendronized bottle brushes (**G2-S-Man**, **G2-S-Mal**, **G2-S-Lac**, **G3-S-Man**, **G3-S-Mal**, and **G3-S-Lac**) may facilitate the investigation and comprehension of heretofore unexplored biological interactions. This advancement has the potential to unveil novel avenues for research in the domain of neurodegenerative diseases.

## CRediT authorship contribution statement

**Tom Kösterke:** Writing – original draft, Visualization, Validation, Methodology, Investigation, Formal analysis, Data curation. **Radika Thakore:** Writing – original draft, Visualization, Validation, Investigation, Formal analysis. **Silvia Moreno:** Writing – review & editing, Supervision, Conceptualization. **Jan Skov Pedersen:** Writing – original draft, Visualization, Validation, Investigation, Formal analysis, Data curation. **Brigitte Voit:** Writing – review & editing, Supervision, Project administration, Funding acquisition. **Oxana Klementieva:** Writing – review & editing, Writing – original draft, Visualization, Supervision, Investigation, Funding acquisition, Formal analysis, Data curation, Conceptualization. **Dietmar Appelhans:** Writing – review & editing, Visualization, Validation, Supervision, Project administration, Funding acquisition, Formal analysis, Data curation, Conceptualization.

## Declaration of competing interest

The authors declare that they have no known competing financial interests or personal relationships that could have appeared to influence the work reported in this paper.

## Data Availability

Data will be made available on request.
