# Supplementary material for: Molecular architectures of glycosylated dendronized bottle brushes in action: Biocompatibility and anti-amyloidogenic activity of pseudo-glycodendrimers
Source: Mater Today Bio. 2025 May 10;32:101771. doi: 10.1016/j.mtbio.2025.101771 (PMC12148669; doi:10.1016/j.mtbio.2025.101771)
Supplement: Multimedia component 1 [file mmc1.docx]

**Supporting Information**

**Molecular architectures of glycosylated dendronized bottle brushes in action: Biocompatibility and anti-amyloidogenic activity of pseudo-glycodendrimers**

**Tom Kösterke,^1,2^ Radika Thakore,^3-5^ Silvia Moreno,^6^ Jan Skov Pedersen,^7^ Brigitte Voit,^1,2^ Oxana Klementieva,^3-5*^ Dietmar Appelhans^1*^**

^1^Leibniz-Institut für Polymerforschung Dresden e.V., Hohe Straße 6, 01069 Dresden, Germany

^2^Organic Chemistry of Polymers, TUD Dresden University of Technology, 01062 Dresden, Germany

^3^Medical Microspectroscopy, Department of Experimental Medical Science, Lund University, 22180 Lund, Sweden

^4^NanoLund, Lund University, 22180 Lund, Sweden

^5^Multipark, Lund University, 22180 Lund, Sweden

^6^Department of Organic and Inorganic Chemistry, University of Alcalá, 28805 Madrid, Spain

^7^Department of Chemistry and Interdisciplinary Nanoscience Center (iNANO), Aarhus University, Gustav Wieds Vej 14, DK-8000 Aarhus C, Denmark

***Corresponding authors:**

Oxana Klementieva: oxana.klementieva@med.lu.se

Dietmar Appelhans: applhans@ipfdd.de

**Content**

|  |  | page |
| --- | --- | --- |
| **1.** | **Methods** | 4 |
| 1.1 | Nuclear Magnetic resonance spectroscopy | 4 |
| 1.2 | Size exclusion chromatography | 4 |
| 1.3 | Dynamic light scattering | 4 |
| 1.4 | Fourier trans fomr infrared spectroscopy | 4 |
| 1.5 | UV-vis spectrometer | 4 |
| 1.6 | Optical photothermal infrared measurements for biohybrid aggregates composed of Aβ and PGDs | 4 |
| 1.7 | Microplate reader | 5 |
| 1.8 | Fluorescence spectrometer | 5 |
| 1.9 | Small angle X-ray scattering | 5 |
| 1.10 | Metabolic assessment of N2a cells | 6 |
| 1.11 | ThT assay for Aβ(1-40) interactions with PGDs | 6 |
| 1.12 | ThT assay for Aβ(1-42) interactions with PGDs | 6 |
| 1.13 | Cryogenic transmission electronic microscopy | 7 |
| **2.** | **Experimental part** | 7 |
| 2.1 | Synthesis of hydroxy-functionalized pseudo-dendrimers (G1-OH - G3-OH) | 7 |
|  | Synthesis of bis-MPA-acetonide | 7 |
|  | Synthesis of bis-MPA-acetonide-anhydride | 7 |
|  | Synthesis of hyperbranched core macromolecule G0-OH | 7 |
|  | Synthesis of the first generation pseudo-dendrimer with acetonide surface groups (G1-Acetonide) | 8 |
|  | Synthesis of the first generation pseudo-dendrimer G1-OH | 8 |
|  | Synthesis of second generation pseudo-dendrimer with acetonide surface groups (G2-Acetonide) | 9 |
|  | Synthesis of second generation pseudo-dendrimer G2-OH | 9 |
|  | Synthesis of third generation pseudo-dendrimer with acetonide surface groups (G3-Acetonide) | 9 |
|  | Synthesis of third generation pseudo-dendrimer G3-OH | 9 |
| 2.2 | Synthesis of yne-functionalized pseudo-dendrimers (G2-Alkyne and G3-Alkyne) | 10 |
|  | Synthesis of 4-pentynoic acid anhydride | 10 |
|  | Synthesis of second generation pseudo-dendrimer with alkyne surface groups | 10 |
|  | Synthesis of third generation pseudo-dendrimer with alkyne surface groups | 10 |
| 2.3 | Synthesis of sugar-functionalized pseudodendrimers (G2-Sugar and G3-Sugar) | 11 |
|  | Synthesis of sugar-acetate-azide | 11 |
|  | Synthesis of sugar-propylazide | 12 |
|  | Synthesis of deprotected sugar-azide | 12 |
|  | Synthesis of pseudo-glycodendrimer G2-Sugar and G3-Sugar | 13 |
|  | Synthesis of pseudo-glycodendrimer G2-Dye-Sugar and G3-Dye-Sugar | 14 |
|  | Synthesis of pseudo-glycodendrimer G2-Spermine(BBB)-AZDye405-S-Maltose | 16 |
|  | Synthesis of deprotected pseudo-glycodendrimer G2-Spermine-AZDye405-S-Maltose | 16 |
|  | Synthesis of pseudo-glycodendrimer G2-TAT-AZDye405-S-Mannose | 16 |

|  |  | page |
| --- | --- | --- |
| **3.** | **Calculation of different parameters to characterize pseudo-dendrimers and pseudo-glycodendrimers** | 17 |
| 3.1 | Degree of branching | 17 |
| 3.2 | Calculation of OH groups for hydroxy-functionalized core macromolecule (G0-OH) and pseudo-dendrimers (G1-OH - G3-OH) | 17 |
| 3.3 | Calculation of sugar groups for pseudo-glycodendrimers | 19 |
| **4.** | **Optimization of click reaction for synthesizing pseudo-glycodendrimers** | 21 |
| **5.** | **Synthesis and characterization of pseudo-dendrimers and pseudo-glycodendrimers: Optimized synthetic steps** | 21 |
| 5.1 | Optimized synthetic pathway of pseudo-glycodendrimers | 21 |
| 5.2 | Azido functionalization of different sugars for final click reaction to realize pseudo-glycodendrimers | 22 |
| 5.3 | New click-reaction conditions for higher degree of functionalization of sugar in pseudo-glycodendrimers | 22 |
| **6.** | **Additional figures and tables** | 24 |
| 6.1 | ^1^H NMR spectra of hydroxy-functionalized pseudo-dendrimers (G1-OH - G3-OH), including core macromolecule (G0-OH) and building blocks | 24 |
| 6.2 | ^1^H NMR spectra of yne-functionalized pseudo-dendrimers (G2-Alkyne, and G3-Alkyne), including G0-Alkyne and building block | 31 |
| 6.3 | ^1^H NMR spectra of sugar molecules | 33 |
| 6.4 | ^1^H NMR spectra of zero generation sugar-functionalized pseudo-dendrimers (G0-S-Man, G0-Dye-Alkyne, and G0-Dye-Man) | 37 |
| 6.5 | ^1^H NMR spectra of 2^nd^ and 3^rd^ generation pseudo-glycodendrimers (G2-Sugar and G3-Sugar) | 39 |
| 6.6 | ^1^H NMR spectra of 2^nd^ and 3^rd^ generation dye-functionalized pseudo-glycodendrimers (G2-Dye-Sugar and G3-Dye-Sugar) | 47 |
| 6.7 | ^1^H NMR spectra of 2^nd^ generation oligoamine/peptide- and dye-functionalized pseudo-glycodendrimers | 53 |
| 6.8 | SEC chromatograms and molecular parameter of (dye-labeled) pseudo-glycodendrimers | 56 |
| 6.9 | Characterization of pseudo-dendrimers and pseudo-glycodendrimers by UV-Vis and fluorescence spectroscopy | 58 |
| 6.10 | Characterization of pseudo-dendrimers and pseudo-glycodendrimers by DLS and FT-IR | 61 |
| 6.11 | Study of ThT assay on the interaction of pseudo-glycodendrimers toward amyloids | 63 |
| 6.12 | Molecular modelling of simplified structures for pseudo-glycodendrimers (G2-Sugar and G3-Sugar) | 65 |
| 6.13 | Visualization of fibrils and aggregates of Aβ(1-40) in presence and absence of G2-S-Lac by cryo-TEM | 66 |
| **7.** | **References** | 72 |

1. **Methods**
   1. **Nuclear magnetic resonance spectroscopy (NMR)**

The ^1^H‑NMR spectra were measured on a Bruker Avance III 500 spectrometer (Bruker Biospin, Germany, 500.13 MHz). The solvents used were chloroform-d3 and DMSO-d6 (Eurisotope) at 300 K. In the evaluation, the ppm values for the chemical shift δ to two decimal places and the values of the coupling constant J (in Hz) were rounded to two decimal place. The internal standard was the signal of the solvent. The abbreviations s = singlet, d = doublet, t = triplet, m = multiplet, br. = broad, dd = doublet of doublets and td = triplet of doublets are used. Quantitative ^13^C NMR spectra were recorded at a temperature of 30 °C on a Bruker Avance 500 NMR spectrometer (Bruker-Biospin, Germany) at 125.75 MHz using inverse gated decoupling and 30 degree flip angle with a pulse delay of 8 s."The solvent signals of DMSO-d6 (39.52 ppm) and CDCl_3_ (77.06 ppm) were used as solvent, lock, and internal standard. Spectra measured in D_2_O were referenced to sodium 3-(trimethylsilyl)-3,3,2,2-tetradeuteropropionate (0.00 ppm), which was added in trace amounts as an internal standard.

**1.2 Size exclusion chromatography (SEC)**

SEC measurements were performed on a SECurity SEC system (Perfect Separation Solutions, Germany). The samples were first passed through a precolumn with a porosity of 30 Å and then through the analytical column with 1000 Å. The injection volume was 100 µL and the selected flow rate was 1 mL/min. As eluent mixture for the column and dissolving the sample was N,N-dimethylacetamide with 5 g/L lithium bromide and 1 % water was used. Measurements took place at 50 °C and were determined by RI-, viscometry, and UV detectors.

**1.3** **Dynamic light scattering (DLS)**

The hydrodynamic diameter of the pseudo-glycodendrimers was recorded on a Zetasizer Nano ZS Instrument (Malvern Instruments, UK), equipped with a He–Ne laser (4 mW, l = 633 nm) at a fixed angle of 1731 (non-invasive backscatter (NIBS) mode). The results were analysed using Zetasizer Software. The pseudo-glycodendrimers was dissolved 24 h before in the solvent. Directly before the measurement the pseudo-glycodendrimer solutions was filtered with a 0.25 µm diameter nylon syringe filter.

**1.4** **Fourier transform infrared spectroscopy (FTIR)**

The FTIR spectroscopic investigations were carried out on a Bruker IFS 28 spectrometer (Bruker Optics GmbH, Ettlingen, Germany) equipped with globar source and MTC detector as well as attenuated total reflection (ATR)-attachment (Optispec, Neeerach, Switzerland) located in the original sample compartment of the spectrometer. FTIR spectra were recorded by collecting 100 scans at a spectral resolution of 2 cm^-1^. The polymer films were prepared by casting from polymer solutions (0.1 – 1 mg/mL) onto germanium (Ge) internal reflexion elements (KOMLAS GmbH, Berlin, Germany).

**1.5 UV-vis spectrometer**

The UV/Vis measurements were made with a SPECORD 210 PLUS from Jena Analytik (Jena, Germany). For the measurements UV cuvettes PLASTIBRAND macro of 10 mm pathlength were used (Carl Roth; Karlsruhe).

**1.6** **Optical photothermal infrared measurements for biohybrid aggregates composed of AB and PGD**

Optical photothermal infrared (OPTIR) spectroscopy was conducted at Lund University’s Integrated Vibration Spectroscopy–Microcosm Laboratory for Molecular-Scale Biogeochemical Research. The infrared (IR) source used was a pulsed, tunable four-stage QCL device, scanning from 1780 to 1500 cm^–1^ at a repetition rate of 100 kHz. The photothermal effect was detected by monitoring the modulation of the green laser (CW 532 nm) intensity induced by the pulsed IR laser. Further details on the instrument set up can be found by Gvazava et al. (2023). The IR power was set to 100 %, with an IR pulse rate of 100 kHz and a Mirage DC of 1.005 V. The green light probe power was set to 25 % for the standard silicon photodiode detector (gain of 10×). Spectral data were collected in reflection mode, with a spectral resolution of 2 cm^–1^. Spectra were averaged over 5 scans, and background spectra were acquired using a built-in reference sample.

**1.7 Microplate reader**

TECAN infinite 200Pro microplate reader equipped with i-control software was used for UV/VIS, Fluorescence and the THT-Assay. To determine the amount of dye molecules per pseudo-dendrimer, UV/VIS measurements were carried out at a wavelength λ between 270 and 450 nm (15 flashes, step size 1 nm, 25 °C). For Fluorescence measurements between 330 and 570 nm (Ex: 291 nm, 15 flashes, integration time 30 μs, lag time 2 μs, 25 °C) with a Corning plate, 96-well Half Area Black/Clear Flat Bottom Polystyrene NBS Microplate. For the THT-Assay the fluorescence emission was measured at 480 nm over 24 h with an interval of 1 min (Ex: 440 nm, 10 flashes, integration time 20 μs, lag time 0 μs, 37 °C). Between measurements, the microplate was shaken orbitally for 5 s with an amplitude of 1 mm.

**1.8 Fluorescence spectrometer**

The fluorescence spectra were measured using an FS5 spectrofluorometer (Edinburgh Instruments) with a temperature-controlled SC-20 holder circulator and a sample thickness of 10 mm. The spectral range was measured from 315 nm to 800 nm with a dwell time of 0.1 s and an excitation wavelength of 291 nm.

**1.9 Small angle X-ray scattering (SAXS)**

SAXS data were collected using the flux-optimized HyperSAXS instrument (Bruker AXS, Germany) at Aarhus University (Lyngsø & Pedersen, 2021), which has a powerful liquid metal-jet Ga X-ray source [28] (Excillum AB, Sweden) The beam is shaped and monochromatized by Montel optics multilayer mirrors (Incoatec, Germany), and the beam is further defined by a two-pinhole collimation system, where the second pinhole, positioned before the sample, is a home-built ‘scatterless’ slit system. The instrumental range of the scattering vector moduli, q, covered 0.01–0.40 Å⁻¹, where q = 4πsin(θ)/λ is the modulus of the scattering vector and 2θ is the scattering angle and λ is the X-ray wavelength. The scattered intensity is detected by a 2D position-sensitive gas detector, VÅNTEC-2000 (Bruker AXS, Germany). The sample was inserted into a flow-through quartz capillary, placed in a vacuum, using an automated sample handler, and the temperature was maintained at 20°C. A water sample was measured as background and subtracted, and the data were processed and normalized to an absolute scale using Milli-Q water and the in-house SUPERSAXS software package (Oliveira, C.L.P.; Pedersen, J.S., unpublished). The samples were measured at 5 and 10 mg/mL and there was very small difference between the data set at the two different concentrations.

An indirect Fourier transformation (IFT) analysis was conducted to derive the pair distance distribution function, p(r), which represents a histogram of distances inside the particles, weighted by the excess scattering length density at the two points. Integrals over the p(r) function provides estimates the radius of gyration (R_g_) of the particles(Glatter et al. 1977, Pederson et al. 1994).

Molecular modelling was performed using rigid-body refinement of structures composed of the dendritic repeating units. These were constructed in PDB format of both G2 and G3 with the respective carbohydrates of Man, Mal and Lac. The repeating units were linked together by soft restraints and excluded volume repulsion was applied to avoid structures with steric overlaps as previously described (Bærentsen et al. 2023). The structure was initially randomized and then optimized by random rotations and translations of the repeating units to fit the SAXS data. The step length of movements is gradually decreased during the optimization.

**1.10 Metabolic Assessment of N2a cells**

Metabolic activity (i.e., cell viability) was assessed using the established protocol described by Gvazava et al. (2023). The water-soluble tetrazolium salt reagent (WST-1) is reduced extracellularly at the plasma membrane of viable cells by mitochondrial dehydrogenases. This enzymatic activity results in the formation of a soluble formazan dye, which is released directly into the culture medium. The amount of formazan produced, which correlates with cellular metabolic activity, was quantified by measuring absorbance in a standard UV–vis plate reader. Lower levels of formazan indicate reduced metabolic activity. N2a cells were incubated with 90 μL of complete medium and 10 μL of WST-1 (Roche, Sigma-Aldrich, USA) for 1–2 hours at 37 °C in a humidified incubator with 5% CO₂. Supernatant optical density was measured at 440 and 650 nm using an Epoch plate reader (BopTeck, USA). Absorbance at 440 nm was used to detect WST-1 conversion by metabolically active cells, while the 650 nm wavelength served as a reference for nonspecific absorbance. Complete medium without cells served as a negative control.

**1.11** **ThT assay for Aβ(1-40) interaction**

The sample preparation of Aβ(1-40) was done under known sonication conditions (extracted from a paper: Biomacromolecules 2011, 12, 3903-390912, 3903-3909) and adapting a protocol to carry out ThT assay in the absence of heparin (Nanomedicine: Nanotechnology, Biology, and Medicine 2019, 17, 198-209) which was further modified for our own experiments.

Stock solutions: The solutions for the ThT assay was prepared in 1 mM PBS buffer. The pH value of the buffer was set to 7.4 and afterwards it was filtered with a 0.8 µm CME syringe filter. Thioflavin T was dissolved in the PBS buffer at least for 1 day before the measurement and stored at 4-6 °C. The concentration of the stock solution for ThT was 60 µM. PGD stock solutions were prepared by the same way with concentrations of 0.6, 6, 15, 30 and 60 µM. Aβ (1-40) (37.5 µM) was dissolved in cold PBS buffer and treated in an ultrasonic bath for 2 minutes. Using only freshly prepared Aβ (1-40). The sonication treatment of Aβ (1-40) results in non-aggregated state as proven by IR spectroscopy in a former study (Klementieva et al., 2011).

For fluorescence measurement a TECAN infinite 200Pro microplate reader was used with a Corning® 96-well Half Area Black/Clear Flat Bottom Polystyrene NBS nonsterile microplate. To each vial of the plate 0.25 µL ThT solution (filtered with 0.25 µm PTFE filter) was added. For the control samples 0.25 µL PBS buffer was added. In the vails for the different PGD concentration 0.25 µL of the stock solutions was added. 100 µL of the Aβ (1-40) stock solution (37.5 µM) was directly added into each vial. The plated was covered with Polyolefin Sealing Film for qPCR and placed into the microplate reader. The final concentration in the vail for ThT was 10 µM, for Aβ (1-40) 25 µM and 0.1, 1, 2.5, 5 and 10 µM for PGD. The fluorescence emission was measured at 480 nm over 24 h with an interval of 1 min (Ex: 440 nm, 10 flashes, integration time 20 μs, lag time 0 μs, 37 °C). Between measurements, the microplate was shaken orbitally for 5 s with an amplitude of 1 mm.

**1.12 ThT assay for Aβ(1-42) interaction**

Fibril growth assays were performed as described by Klementieva et al. (2017). In order to avoid pre-existing seeds, recombinant Aβ(1-42) monomers were isolated using HPLC. The fraction containing monomers was kept on ice, and the kinetics were run immediately after determining the concentration of monomers. Specifically, for fibril formation, ThT kinetic assays were conducted by incubating 5 μM Aβ1-42 with 6 μM ThT at 37 °C without agitation in a black polystyrene 96-well plate with a clear bottom and PEG coating (Corning 3881). ThT fluorescence, which is proportional to the amount of amyloid fibrils (Sulatskaya et al., 2017) was measured from the bottom of the plate every 60 seconds using a plate reader (Fluostar Omega BMG Labtech) with a 440 nm excitation filter and a 480 nm emission filter.

**1.13 Cryogenic transmission electron microscopy (cryo-TEM)**

Cryo-TEM images were recorded in Libra 120 microscope (Carl Zeiss Microscopy Deutschland GmbH, Oberkochen, Germany). 2 µL of specimen solution was placed onto each side of a holey carbon TEM grid (Quantifoil R3.5/1, 300 mesh), blotted with filter paper and vitrified in liquid ethane at -178 °C using a Grid Plunger (Leica Microsystems GmbH, Wetzlar, Germany). Frozen grids were transferred into Gatan 626 (Gatan GmbH, München, Germany) cryo-TEM holder. Images were recorded at an accelerating voltage of 120 kV while keeping the specimen at -170 °C. To validate requested Aβ(1-40) solution states as fibrils and/or undefined aggregates, composed of Aβ(1-40) and **G2-S-Lac**, a general view on several spots was done to further select individual spots for the visualization of Aβ(1-40) fibrils and aggregates through further enlargements of selected areas on the spot(s). ThT samples with **G2-S-Lac** were prepared and let kept for 24 h at 37°C, before cryo-TEM study was carried out. Further details are indicated in the figure captions of **Figures 7** and **S63-S68**.

1. **Experimental part**

**2.1 Synthesis of hydroxy-functionalized pseudo-dendrimers (G1-OH - G3-OH)**

Synthesis of bis-MPA-acetonide

In a round-bottom flask Bis-MPA (35.0 g, 1.0 eq.) and DMP (32.6 g, 26.3 mL, 1.2eq) were completely dissolved in acetone (270 mL). To this solution pTsOH (50.08 mg, 0.001 eq.) as catalyst was added and stirred overnight (19 h) at rt. Then reaction solution was quenched with EtOH/NH_3_(30 %)-solution (1:1; 0.4 mL). After this the acetone was distilled off by rotary evaporator under reduced pressure. The obtained residue was taken up in DCM (250 mL) and extracted twice with water through the use of a separatory funnel. The organic phase was separated, dried over Na_2_SO_4_, and, finally, filtered with a glass frit. The organic solvent was distilled off by rotary evaporator under reduced pressure. The non-solid product, obtained, was dried in a vacuum oven overnight at 40 °C. The product was yielded as a white solid (27.48 g, 61 %).

^1^H NMR (500 MHz, CDCl_3_): δ (ppm) = 1.23 (s, 3 H), 1.43 (s, 3 H), 1.46 (s, 3 H), 3.69 (d, *J*=11.66 Hz, 2 H), 4.19 (d, *J*=11.66 Hz, 2 H).

**Synthesis of bis-MPA-acetonide-anhydride**

In a heated out two-neck flask with argon bis-MPA-acetonide (19.00 g, 2.0 eq.) was dissolved in 70 mL of dry DCM. After 1 h of stirring the starting material was completely dissolved. A second heated out two-neck flask under argon DCC (11.25 g, 1.0 eq.) was dissolved in 20 mL of dry DCM. The suspension of DCC was added dropwise to the bis-MPA-acetonide solution. After 22 h stirring at rt the reaction solution was cooled down in a dry ice/acetone bath (-78 °C). The mixture was filtered with a glass frit and washed with cold DCM. The collected organic solvent was removed under reduced pressure, and the obtained solid was dried in a vacuum oven overnight at 40 °C. The product was obtained as a slightly yellow viscous liquid (18.12 g, 100 %).

^1^H NMR (500 MHz, CDCl_3_): δ (ppm) = 1.25 (s, 6 H), 1.41 (s, 6 H), 1.44 (s, 6 H), 3.69 (d, *J*=11.98 Hz, 4 H), 4.22 (d, *J*=11.66 Hz, 4 H).

**Synthesis of hyperbranched core macromolecule G0-OH**

In a two-neck flask bis-MPA (20.0 g, 2.0 eq.) and pTsOH (31.1 mg, 0.01 eq.) were added. The oil bath was set to a target temperature of 185 °C. For the first 2 h the flask was floated with argon and heated up until the starting material was completely liquefied. A second cannula was inserted into the stopper to flush out condensation water. The compounds melted at 176 °C. This temperature was maintained during the entire reaction time. When all compounds were liquefied, a vacuum was applied to the reaction mixture and stirred for 8 h. After that the flask was cooled down to rt and 70 mL of THF were added to dissolve the solidified reaction product under stirring at 45 °C after 2 h. A second flask with 280 mL Et_2_O was cooled with a dry ice/acetone bath to -78 °C. The THF-containing polymer solution was added dropwise to the cold Et_2_O. The product was precipitated as white spheres. The white solid was filtered and washed carefully with cooled Et_2_O. The solid was dried in a vacuum oven at 40 °C overnight to remove residual THF. To obtain powder-like G0-OH, the polycondensate G0-OH was dissolved in water again to freeze-dry the aqueous polymer solution. The product was obtained as a white powder-like solid (14.22 g).

GPC: M_n_ = 4700 g/mol, Ð = 1.88, dn/dc = 0.079. ^1^H NMR (500 MHz, DMSO-d_6_): δ (ppm) = 1.03 (s, CH_3_, terminal), 1.08 (br. s., CH_3_, linear), 1.18 (br. s., CH_3_, dendritic), 3.40 - 3.55 (m, CH_2_OH), 4.04 - 4.21 (m, CH_2_OCO), 4.60 (br. s., OH, t), 4.91 (br. s., OH, l), 12.87 (br. s., COOH).

**Synthesis of the first generation pseudo-dendrimer with acetonide surface groups (G1-Acetonide)**

Poly-bis-MPA (3.92 g, 1.0 eq.) and DMAP (4.98 g, 1.2 eq.) were weighed in a 250 mL three neck flask under inert gas. 20 mL of dry pyridine was added evenly with a syringe under stirring. A heat gun was used for the complete solvation of the starting materials. Bis-MPA-acetonide-anhydride (13.44 g, 1.2 eq. calculated on the OH-groups of G0-0H) was dissolved in 40 mL of DCM in a one neck flask. The dissolved bis-MPA-acetonide-anhydride was added slowly to the reaction mixture and stirred at rt for 17.5 h. To stop the reaction, 2 mL of water was added and transferred into a separating funnel with 30 ml DCM. In the first step, the reaction mixture was washed with 2% HCl solution. Then two times with 10% Na_2_HSO4 solution, saturated NaHCO_3_ solution and saturated NaCl solution. The organic phase was dried over Na_2_SO_4_ and filtered with a glass frit. Under reduced pressure at 40 °C the solvent was removed, and the non-solid product was dried in the vacuum oven at 40 °C overnight. The product obtained as a light orange solid (6.5 g).

^1^H NMR (500 MHz, CDCl_3_): δ (ppm) = 1.15 (br. s., CH_3,_ terminal) 1.28 (br. s., CH_3,_ linear) 1.36 (br. s., CH_3,_ dendritic) 1.41 (br. s., OCCH_3_) 3.62 (d, *J*=11.30 Hz, CH_2_OC(CH_3_)_2_) 4.09 - 4.19 (m, CH_2_OC(CH_3_)_2_) 4.22 - 4.38 (m, CH_2_OCO).

**Synthesis of the first generation pseudo-dendrimer G1-OH**

G1-Acetonide (2.90 g, 1.0 eq.) was weighed into a one-necked flask and heated to 40 °C with 60 mL of MeOH in a water bath while stirring. After G1-Acetonide was completely dissolved, DOWEX 50W-X2 (2.95 g) was added, and the reaction solution was stirred for 6 h. The DOWEX was filtered off using a glass frit and the solvent was removed under reduced pressure. The aqueous product solution was freeze-dried overnight and received as a light orange solid (2.34 g).

GPC: M_n_ = 18300 g/mol, Ð = 1.36, dn/dc = 0.079. ^1^H NMR (500 MHz, DMSO-d_6_): δ (ppm) = 1.01 (s, CH_3_, terminal) 1.09 (br. s., CH_3_, linear) 1.17 (br. s., CH_3_, dendritic) 3.36 - 3.52 (m, CH_2_OH) 4.05 - 4.27 (m, CH_2_OCO) 4.54 (br. s., OH, t).

**Figure S1.** Synthesis of G1-OH through the conversion G0-OH with AB_2_-monomer G1-acetonides.

**Synthesis of the second generation pseudo-dendrimer with acetonide surface groups (G2-Acetonide)**

G1-OH (3.95 g, 1.0 eq.) and DMAP (4.44 g, 1.05 eq.) were weighed in a two-neck flask under argon and dissolved in 25 mL pyridine. In a second flask under argon bis-MPA-acetonide-anhydride (12.0 g, 1.05 eq.) was added and dissolved in 20 mL anhydrous DCM under stirring. Both solutions were combined and stirred overnight at rt (20.75 h). To stop the reaction, 2 mL of water were added and transferred into a separating funnel with 30 ml DCM. For the first step the reaction mixture was washed with 2% HCl solution, then two times with 10% Na_2_HSO4 solution, saturated NaHCO_3_ solution, and saturated NaCl solution. The organic phase was dried over Na_2_SO_4_ and filtered with a glass frit. The solvent was removed under reduced pressure at 40 °C and dried in the vacuum oven at 40 °C overnight. The product was obtained as a light orange solid (7.94 g).

^1^H NMR (500 MHz, CDCl_3_): δ (ppm) = 1.15 (br. s., CH_3,_ terminal), 1.21 (s, CH_3,_ linear), 1.28 (br. s., CH_3,_ dendritic), 1.34 (br. s, OCCH_3_), 1.42 (br. s, OCCH_3_), 3.62 (d, *J*=11.66 Hz, CH_2_OC(CH_3_)_2_), 4.14 (d, *J*=10.72 Hz, CH_2_OC(CH_3_)_2_), 4.22 - 4.37 (m, CH_2_OCO).

**Synthesis of the second generation pseudo-dendrimer G2-OH**

In a one-neck flask weight G2-Acetonide (7.94 g, 1.0 eq.) was dissolved in MeOH (150 mL) under stirring at 40 °C, using a water bath. After G2-Acetonide was totally dissolved, Amberlite IR 120H (11 g) was added to the solution to let stir it for 5 h at rt. The Amberlite was filtered off with a glass frit, and the organic solvent was removed by a rotary evaporator under reduced pressure. Then the residue was freeze-dried overnight to obtain the product as a light orange solid (6.08 g).

GPC: M_n_ = 19000 g/mol, Ð = 1.49 dn/dc = 0.080. ^1^H NMR (500 MHz, DMSO-d6): δ (ppm) = 1.01 (s, CH_3_, terminal), 1.04 (s, CH_3_, linear), 1.12 - 1.25 (m, CH_3_, dendritic), 3.38 - 3.50 (m, CH_2_OH), 3.99 - 4.35 (m, CH_2_OCO).

**Synthesis of the third generation pseudo-dendrimer with acetonide surface groups (G3-Acetonide)**

The reaction was completely carried out in reaction flasks under inert gas condition. In a two-neck flask G2-OH (1.44 g, 1.0 eq.) and DMAP (0.69 g, 1.05 eq.) were dissolved in pyridine (10 mL). In a second flask Bis-MPA-acetonide-anhydride (2.00 g, 1.05 eq.) was dissolved in anhydrous DCM (34 mL) under stirring. The unified solutions were stirred overnight at rt. The reaction solution was quenched with water (2 mL) and, then, transferred into a separating funnel, filled with additional DCM (30 mL). The reaction mixture was washed with 10wt% aqueous Na_2_HSO4 solution, saturated NaHCO_3_ solution and saturated NaCl solution. The organic phase was dried over Na_2_SO_4_ and filtered by a glass frit. The organic solvent was removed by a rotary evaporator under reduced pressure at 40 °C. The residue was dried in the vacuum oven at 50 °C overnight. The product was dissolved in toluene to remove any residual pyridine. The solvent was removed at reduced pressure at 40°C. The product was then dried in a vacuum oven at 50°C again. The product is a light orange solid (2.15 g).

^1^H NMR (500 MHz, CDCl_3_): δ (ppm) = 1.14 (br. s., CH_3,_ terminal), 1.20 (br. S., CH_3,_ linear), 1.29 (br. s.,CH_3,_ dendritic), 1.36 (br. s, OCCH_3_), 1.42 (br. s, OCCH_3_), 3.57 - 3.78 (m, CH_2_OC(CH_3_)_2_), 4.16 (d, *J*=10.80 Hz, CH_2_OC(CH_3_)_2_), 4.20 - 4.44 (m, CH_2_OCO).

**Synthesis of the third generation pseudo-dendrimer G3-OH**

In a one-neck flask G3-Acetonide (2.15 g, 1.0 eq.) was dissolved in MeOH (50 mL) under stirring at 40°C, using a water bath. After G3-Acetonide was totally dissolved, DOWEX 50W-X2 (3.99 g) was added to the solution to let stirred for 5 h at rt. The DOWEX was separated from the organic solution, using a glass frit. The organic solvent was distilled off by rotary evaporator under reduced pressure. The crude product was freeze-dried overnight to receive it as a light orange solid (1.30 g).

GPC: M_n_ = 32100 g/mol, Ð = 1.52, dn/dc = 0.078. ^1^H NMR (500 MHz, DMSO-d6): δ (ppm) = 1.02 (br. s., CH_3_, terminal) 1.07 (br. s., CH_3_, linear) 1.17 (br. s., CH_3_, dendritic) 3.36 - 3.55 (m, CH_2_OH) 4.02 - 4.25 (m, CH_2_OCO).

**2.2** **Synthesis of yne-functionalized pseudo-dendrimers (G2-Alkyne, and G3-Alkyne)**

**Synthesis of 4-pentynoic anhydride**

The reaction was carried out in a heated out Schlenk flask under argon atmosphere. Thus 4-pentynoic acid (4.74 g, 2.0 eq.) was dissolved in dry DCM (30 mL). In second 100 mL Schlenk flask, kept under inert gas, DDC (5.01 g, 1.0 eq.) was dissolved in dry DCM (20 mL) under stirring. Then the first solution of pentynoic acid was added to the DCC solution. The resulting reaction solution was stirred overnight at rt. After 17.5 h of stirring the reaction solution was cooled down in a dry ice/acetone bath (-78 °C) to let precipitate side products of this conversion step. The mixture was filtered with a glass frit, and the resulting separated solid was washed with cold DCM. The collected organic solution was distilled to separate DCM by rotary evaporator under reduced pressure. The product is received as a clear brown liquid (4.30 g, 100 %).

^1^H NMR (500 MHz, CDCl_3_): δ (ppm) = 2.02 (t, *J*=2.68 Hz, 2 H), 2.55 (td, *J*=7.33, 2.68 Hz, 4 H), 2.73 (t, *J*=7.30 Hz, 4 H).

**Synthesis of second generation pseudo-dendrimer with alkyne surface groups**

In a two-neck flask G2-OH (1.50 g, 1.0 eq.) and DMAP (1.90  g, 1.2 eq. calculated on the OH-groups of G2-OH) were added under argon atmosphere and dissolved in anhydrous pyridine (8 mL). In a second flask, kept under argon atmosphere, 4-Pentynoic acid anhydride (2.78 g, 1.2 eq. calculated on the OH-groups of G0-OH) was dissolved in dry DCM (22 mL). This solution was added with a syringe to the first prepared solution. The resulting reaction solution was stirred overnight at rt (23.5 h). The reaction solution was transferred into a separating funnel. The organic solution was subsequently washed 1 time with 2 % HCl solution and two times each with 10 % NaHSO_4_, saturated NaHCO_2_ and saturated NaCl. The resulting organic phase was separated and dried over Na_2_SO_4_. Thereafter the organic phase was filtered, and the organic solvent was distilled off by rotary evaporator under reduced pressure. Overnight the product, obtained, was totally dried at 40 °C in a vacuum oven. The product was received as a brown, highly viscous liquid (1.80 g).

^1^H NMR (500 MHz, CDCl_3_): δ (ppm) = 1.17 - 1.39 (m, CH_3_), 2.02 (br. s., CCH), 2.48 (br. s., CH_2_), 2.57 (t, *J*=7.20 Hz, CH_2_), 4.12 - 4.41 (m, CH_2_OCO).

**Synthesis of third generation pseudo-dendrimer with alkyne surface groups**

In a two-neck flask G3-OH (1.00 g, 1.0 eq.) and DMAP (1.38 g, 1.2 eq. calculated on the OH-groups of G2-OH) were added under argon atmosphere and dissolved in anhydrous pyridine (5 mL). In a second flask, kept under argon atmosphere, 4-Pentynoic acid anhydride (2.01 g, 1.2 eq. calculated on the OH-groups of G0-OH) was dissolved in dry DCM (15 mL). This solution was added with a syringe to the first prepared solution. The resulting reaction solution was stirred overnight at rt (20 h). The reaction solution was transferred into a separating funnel. The organic solution was subsequently washed 1 time with 2 % HCl solution and two times each with 10 % NaHSO_4_, saturated NaHCO_2_ and saturated NaCl. The resulting organic phase was separated and dried over Na_2_SO_4_. Thereafter the organic phase was filtered, and the organic solvent was distilled off by rotary evaporator under reduced pressure. Overnight the product, obtained, was totally dried at 40 °C in a vacuum oven. The product was received as a brown, highly viscous liquid (1.21 g).

^1^H NMR (500 MHz, CDCl_3_): δ (ppm) = 1.18 - 1.37 (m, CH_3_) 2.02 (br. s., CCH) 2.49 (d, J=6.00 Hz, CH_2_) 2.56 (d, J=6.31 Hz, CH_2_) 4.17 - 4.37 (m, CH_2_OCO).

**2.3** **Synthesis of sugar-functionalized pseudo-dendrimers (G2-Sugar, and G3-Sugar)**

**Synthesis of sugar‑acetate-azide**

The reaction was completely carried out with baked-out round-bottom flask under inert gas condition. sugar-acetate (1.0 eq.) was dissolved in dry DCM (60 mL). To this cooled down solution by an ice-bath azidopropanol (1.5 eq.) was added dropwise by a syringe as well as borontrifluoride-diethyletherate (3.0 eq.) by the same procedure. After removing the ice bath, the reaction mixture was stirred for 48 h. The solution was transferred into a separator funnel, equipped with additional DCM. The organic phase was carefully washed with saturated NaHCO_3_ solution which was accompanied by an intense gas evolution. The aqueous phase was extracted one time with DCM in excess. The organic phases were collected, dried over Na_2_SO_4_, and filtrated by a glass frit. The organic solvent was distilled off by a rotary evaporator under reduced pressure. The crude product was obtained as a highly viscous red liquid and purified by a column chromatography with *n*-hexane/ethylacetate (70:30). The product was obtained as a white solid.

**Table S1.** Used reactants for the azide functionalization of sugars.

| Sugar-acetate-azide | Starting material | Azidopropanol | BF_3_^.^EtO_2_ | DCM | Yield |
| --- | --- | --- | --- | --- | --- |
| α-D-Mannose-tetraacetate-propylazide | 15.0 g^a^ | 5.83 g | 16.36 g | 60 mL | 27 % (4.39 g) |
| β-D-Maltose-heptaacetate-propylazide | 8.0 g^b^ | 1.82 g | 5.02 g | 50 mL | 32 % (2.71 g) |
| β-D-Lactose-heptaacetate-propylazide | 5.1 g^c^ | 1.52 g | 3.20 g | 20 mL | 22 % (1.19 g) |
| ^a^α-D-Mannose-pentaacetate. ^b^β-D-Maltose-octaacetate. ^c^β-D-Lactose-octaacetate. | | | | | |

**α-D-Mannose-pentaacetate-propylazide**

^1^H NMR (500 MHz, CDCl_3_): δ (ppm) = 1.83 - 1.95 (m), 2.00 (s), 2.05 (s), 2.10 (s), 2.16 (s), 3.43 (t, *J*=6.00 Hz), 3.49 - 3.58 (m), 3.78 - 3.86 (m), 3.97 (br. s.), 4.12 (d, *J*=12.30 Hz), 4.25 - 4.31 (m), 4.81 (s), 5.21 - 5.35 (m).

**β-D-Maltose-heptaacetate-propylazide**

^1^H NMR (500 MHz, CDCl_3_): δ (ppm) = 1.57 (s) 1.77 - 1.91 (m) 2.01 (s) 2.03 (s) 2.05 (s) 2.11 (s) 2.15 (s) 2.18 (s) 3.30 - 3.40 (m) 3.58 - 3.65 (m) 3.66 - 3.72 (m) 3.92 (quin, J=5.00 Hz) 3.95 - 4.03 (m) 4.06 (dd, J=12.20, 2.00 Hz) 4.21 - 4.29 (m) 4.50 (dd, J=12.20, 2.60 Hz) 4.54 (d, J=8.00 Hz) 4.80 - 4.89 (m) 5.06 (t, J=9.80 Hz) 5.26 (t, J=9.20 Hz) 5.37 (t, J=10.00 Hz) 5.42 (d, J=4.30 Hz).

**β-D-Lactose-heptaacetate-propylazide**

^1^H NMR (500 MHz, CDCl_3_): δ (ppm) = 1.55 (s) 1.79 - 1.89 (m) 1.97 (s) 2.05 (s) 2.07 (s) 2.13 (s) 2.16 (s) 3.31 - 3.41 (m) 3.56 - 3.64 (m) 3.80 (t, J=9.50 Hz) 3.85 - 3.95 (m) 4.06 - 4.17 (m) 4.46 - 4.54 (m) 4.90 (dd, J=9.50, 8.00 Hz) 4.97 (dd, J=10.50, 3.50 Hz) 5.12 (dd, J=10.50, 7.80 Hz) 5.20 (t, J=9.30 Hz) 5.36 (d, J=3.40 Hz).

**Synthesis of sugar-propylazide**

The reaction was completely carried out with baked-out round-bottom flask under inert gas condition. sugar-acetate-azide (1.0 eq.) was dissolved in MeOH. Then sodium methylate (25wt%) in MeOH (2.0 eq.) was added and the reaction solution was stirred for 2 h at rt. The cationic exchanger resin, Amberlite IR 120 H, was added and the reaction solution was stirred for another 2 h. With this the pH of the reaction solution obtained the value of 7, the cationic exchanger resin was filtered off. The organic solvent was distilled off by a rotary evaporator under reduced pressure. After freeze drying of the residue the product was received as a slightly red solid.

**Table S2.** Used reactants for the deprotection of sugar-acetate-propylazide.

| Sugar-propylazide | Starting material | Sodium methylate | Amberlite IR 120 H | MeOH | Yield |
| --- | --- | --- | --- | --- | --- |
| α-D-Mannose-propylazide | 2.93 g^a^ | 0.6 mL | 3.0 g | 60 mL | 100 %  (1.83 g) |
| β-D-Maltose-propylazide | 1.26 g^b^ | 0.8 mL | 1.28 g | 40 mL | 96 % (717 mg) |
| β-D-Lactose-propylazide | 1.20 g^c^ | 0.8 mL | 2.1 g | 40 mL | 100 % (704 mg) |
| ^a^ α-D-Mannose-tetraacetate-propylazide. ^b^ β-D-Maltose-heptaacetate-propylazide. ^c^ β-D-Lactose-heptaacetate-propylazide. | | | | | |

**α-D-Mannose-propylazide**

^1^H NMR (500 MHz, DMSO-d_6_): δ (ppm) = 1.78 (s), 3.25 - 3.32 (m), 3.33 - 3.48 (m), 3.57 - 3.62 (m), 3.65 (s), 4.60 (s).

**β-D-Maltose-propylazide**

^1^H NMR (500 MHz, DMSO-d_6_): δ (ppm) = 1.78 (quin, J=6.50 Hz), 2.95 - 3.13 (m), 3.23 (m), 3.36 - 3.50 (m), 3.51 - 3.62 (m), 3.66 - 3.73 (m), 3.76 - 3.84 (m), 4.16 (d, J=7.88 Hz), 4.37 - 4.50 (m), 4.84 (dd, J=12.77, 4.89 Hz), 5.01 (d, J=3.47 Hz), 5.07 (d, J=4.73 Hz), 5.38 (d, J=5.99 Hz), 5.46 (d, J=2.52 Hz).

**β-D-Lactose-propylazide**

^1^H NMR (500 MHz, DMSO-d_6_): δ (ppm) = 1.78 (quin, J=6.60 Hz), 3.01 (t, J=7.70 Hz), 3.40 - 3.48 (m), 3.48 - 3.56 (m), 3.57 - 3.64 (m), 3.74 (br. d, J=11.70 Hz), 3.77 - 3.84 (m), 4.14 - 4.24 (m), 4.49 (d, J=15.45 Hz), 4.56 - 4.68 (m), 4.73 (br. s.), 5.05 (br. s.), 5.09 (br. s.).

**Synthesis of deprotected sugar-azide**

**Figure S2.** Synthesis from alpha-D-mannose-pentaacetate via alpha-D-mannose-tetraacetate-propylazide to alpha-D-mannose-propylazide.

**Figure S3.** Synthesized sugar-azides for shell functionalization of the pseudo-dendrimers.

**Synthesis of Pseudo-glycodendrimer G2- and G3-S-sugar**

The reaction was completely carried out in baked-out Schlenk tube under inert gas. G2-Alkyne or G3‑Alkyne (1.0 eq.) and sugar-propylazide (1.2 eq. calculated on the alkyne-groups of G2‑Alkyne or G3‑Alkyne) were dissolved in dry DMSO under stirring. To the reaction mixture DIPEA (0.1 eq.) and copper(I)-iodid (0.1 eq.) were added. The reaction was stirred for 3 days at rt. After that, the mixture was quenched with water. The mixture was transferred to a dialysis membrane tube. A Spectra/Por® 7 membrane tube for Carl Roth with a MWCO of 2000 Da out of regenerative cellulose was used. Thus, the sugars with a molar mass from 263.25 g/mol to 425.39 g/mol and other additives of the reaction were exchanged. The dialysis membrane was put in a 1 L tumbler with water for over 2 days. The water was changed 3 times per day. The aqueous product solution was freeze-dried to obtain a white powder.

**Table S3.** Used reactants for the synthesis of G2- and G3-S-sugar.

| GX-S-Sugar | GX-Alkyne | Sugar-propylazide | CuI | DIPEA | DMSO | Yield | |
| --- | --- | --- | --- | --- | --- | --- | --- |
| G2-S-Mannose | 151.4 mg^a^ | 225.9 mg^c^ | 16.3 mg | 13 µL | 1.5 mL | 64 % (233.8 mg) | |
| G2-S-Maltose | 149.6 mg^a^ | 391.2mg^d^ | 20.8mg | 13 µL | 1.5 mL | 73 % (334.7 mg) | |
| G2-S-Lactose | 150.4 mg^a^ | 390.4 mg^e^ | 15.3 mg | 13 µL | 1.5 mL | 69 % (337.2 mg) | |
| G3-S-Mannose | 110.5 mg^b^ | 170.8 mg^c^ | 11.1 mg | 9 µL | 1.5 mL | 61 % (139.3 mg) | |
| G3-S-Maltose | 101.1 mg^b^ | 252.1 mg^d^ | 9.8 mg | 9 µL | 1.5 mL | 91 % (214.9 mg) | |
| G3-S-Lactose | 70.3 mg^b^ | 190.8 mg^e^ | 7.7 mg | 7 µL | 1.0 mL | 71 % (123.1 mg) | |
| ^a^ G2-Alkyne ^b^ G3-Alkyne. ^c^ α-D-Mannose-propylazide. ^d^ β-D-Maltose-propylazide. ^e^ β-D-Lactose-propylazide | | | | | | |  |

**G2-S-Mannose**

^1^H NMR (500 MHz, DMSO-d_6_): δ (ppm) = 1.09 (s), 1.17 (s), 1.23 (s), 2.02 (s), 2.63 (br. s.), 2.83 (br. s.), 3.29 (s), 3.39 (br. s.), 3.46 (br. s.), 3.62 (br. s.), 4.10 (br. s.), 4.21 - 4.42 (m), 4.50 (br. s.), 4.59 (br. s.), 4.65 (br. s.), 7.81 (br. s.).

**G2-S-Maltose**

^1^H NMR (500 MHz, DMSO-d_6_): δ (ppm) = 1.10 (s), 1.18 (s), 1.24 (s), 2.02 (br. s), 2.63 (br. s), 2.83 (br. s), 3.07 (br. s), 3.25 (br. s.), 3.36 - 3.52 (m), 3.53 - 3.66 (m), 3.66 - 3.74 (m), 3.76 (br. s.), 4.03-4.15 (m), 4.18 (d, J=6.00 Hz), 4.37 (br. s.), 4.46 (br. s), 4.83 (s), 4.86 (s), 5.02 (br. s), 5.12 (br. s), 5.38 (br. s), 5.48 (br. s), 7.82 (br. s.).

**G2-S-Lactose**

^1^H NMR (500 MHz, DMSO-d_6_): δ (ppm) = 1.10 (s), 1.17 (s), 1.23 (s), 2.02 (br. s), 2.63 (br. s), 2.83 (br. s.), 3.06 (br. s.), 3.32 (br. s.), 3.47 (br. s.), 3.53 (br. s.), 3.63 (br. s.), 3.75 (br. s.), 3.99 - 4.16 (m), 4.21 (br. s.), 4.37 (br. s.), 4.47 (br. s.), 4.51 (br. s.), 4.62 (br. s.), 4.68 (br. s.), 4.72 (br. s.), 5.05 (br. s.), 5.14 (br. s.), 7.82 (br. s.).

**G3-S-Mannose**

^1^H NMR (500 MHz, DMSO-d_6_): δ (ppm) = 1.09 (s), 1.13 (s), 1.24 (s), 2.02 (br. s.), 2.63 (br. s.), 2.82 (br. s.), 3.39 (br. s.), 3.46 (br. s.), 3.62 (br. s.), 4.10 (br. s.), 4.27 - 4.42 (m), 4.50 (br. s.), 4.59 (br. s.), 4.62 - 4.72 (m), 7.82 (br. s.).

**G3-S-Maltose**

^1^H NMR (500 MHz, DMSO-d_6_): δ (ppm) = 1.10 (br. s.), 1.16 (br. s), 1.24 (br. s.), 2.02 (br. s.), 2.63 (br. s.), 2.82 (br. s.), 3.07 (br. s.), 3.25 (br. s.), 3.35 - 3.54 (m), 3.54 - 3.67 (m), 3.67 - 3.74 (m), 3.77 (br. s.), 3.97 - 4.16 (m), 4.19 (d, J=5.20 Hz), 4.37 (br. s.), 4.47 (br. s.), 4.85 (d, J=14.50 Hz), 5.02 (br. s.), 5.13 (br. s.), 5.39 (br. s.), 5.49 (br. s.), 7.82 (br. s.).

**G3-S-Lactose**

^1^H NMR (500 MHz, DMSO-d_6_): δ (ppm) = 1.08 (br. s.), 1.12 (br. s.), 1.23 (br. s.), 2.01 (br. s.), 2.63 (br. s.), 2.81 (br. s.), 3.05 (br. s.), 3.62 (br. s.), 3.74 (br. s.), 3.96 - 4.16 (m), 4.21 (br. s.), 4.36 (br. s.), 4.50 (br. s.), 4.57 (br. s.), 4.69 (br. s.), 4.76 (br. s.), 5.08 (br. s.), 5.18 (br. s.), 7.79 (br. s.).

**Synthesis of Pseudo-glycodendrimer G2- and G3-Dye-S-Sugar**

The reaction was completely carried out in baked-out Schlenk tube under inert gas. G2-Alkyne or G3-Alkyne (1.0 eq.) was dissolved in dry DMSO. Then CuI (0.1 eq.) and DIPEA (0.1 eq. calculated on the alkyne-groups of G2-Alkyne or G3-Alkyne) were added. In a second baked-out Schlenk tube under argon atmosphere AZ-Dye-405 was dissolved in dry DMSO to get a concentration of 1 mg per 100 µL for the dye molecule. Then 2.0 eq. of the AZ-Dye-405 solution was added to the pseudo-dendrimer solution under protection atmosphere. The resulting reaction solution was stirred for 24 h at rt. After 1 day of stirring sugar-propylazide (1.2 eq. calculated on the alkyne-groups of pseudo-dendrimer, G2-Alkyne or G3-Alkyne) was added to the reaction mixture. The reaction was additionally stirred for 3 days at rt. After that, the mixture was quenched with water and purified by dialysis (2000 Da RC membrane) over 2 days, changing the water 3 times per day. The aqueous product solution was freeze-dried to obtain the desired product, G2-Dye-S-Sugar or G3-Dye-S-Sugar, as a slightly colored powder.

**Table S4.** Used reactants for the synthesis of G2- and G3-Dye-S-Sugar.

| GX-S-Sugar | GX-Alkyne | AZDye405 | Sugar-propylazide | CuI | DIPEA | DMSO | Yield | | |
| --- | --- | --- | --- | --- | --- | --- | --- | --- | --- |
| G2-Dye-S-Mannose | 80.4 mg^a^ | 0.33 mL | 120.6 mg^c^ | 7.8 mg | 7 µL | 2.0 mL | 63 % (138.0 mg) | | |
| G2-Dye-S-Maltose | 79.7 mg^a^ | 0.33 mL | 198.5 mg^d^ | 8.0 mg | 7 µL | 2.0 mL | 66 % (187.2 mg) | | |
| G2-Dye-S-Lactose | 71.4 mg^a^ | 0.29 mL | 167.7mg^e^ | 6.9 mg | 6.5 µL | 2.0 mL | 76 % (169.4 mg) | | |
| G3-Dye-S-Mannose | 80.0 mg^b^ | 0.19 mL | 128.7 mg^c^ | 8.7 mg | 8 µL | 2.0 mL | 61 % (114.4 mg) | | |
| G3-Dye-S-Maltose | 80.9 mg^b^ | 0.18 mL | 206.4 mg^d^ | 8.3 mg | 8 µL | 2.0 mL | 60 % (176.2 mg) | | |
| G3-Dye-S-Lactose | 71.3 mg^b^ | 0.16mL | 182.2 mg^e^ | 7.6 mg | 7 µL | 2.0 mL | 82 % (159.2 mg) | | |
| ^a^ G2-Alkyne ^b^ G3-Alkyne. ^c^ α-D-Mannose-propylazide. ^d^ β-D-Maltose-propylazide. ^e^ β-D-Lactose-propylazide | | | | | | | |  |  |

**G2-AZDye405-S-Mannose (G2-Dye-S-Man, TK-049)**

^1^H NMR (500 MHz, DMSO-d_6_): δ (ppm) = 1.09 (br. s.), 1.17 (br. s.), 1.24 (br. s.), 2.02 (br. s.), 2.63 (br. s.), 2.83 (br. s.), 3.39 (br. s.), 3.47 (br. s), 3.62 (br. s.), 3.99 - 4.22 (m), 4.33 (br. s.), 4.38 (br. s.), 4.51 (br. s.), 4.59 (s), 4.67 (br. s.), 7.81 (br. s.).

**G2-AZDye405-S-Maltose (G2-Dye-S-Mal, TK-050)**

^1^H NMR (500 MHz, DMSO-d_6_): δ (ppm) = 1.10 (br. s.), 1.18 (br. s.), 1.24 (br. s.), 2.02 (br. s.), 2.63 (br. s.), 2.83 (br. s.), 3.07 (br. s.), 3.25 (br. s.), 3.36 - 3.54 (m), 3.54 - 3.66 (m), 3.66 - 3.73 (m), 3.73 - 3.82 (m), 3.96 - 4.15 (m), 4.18 (d, J=6.31 Hz), 4.37 (br. s.), 4.43 - 4.57 (m), 4.85 (d, J=11.03 Hz), 5.02 (br. s.), 5.14 (br. s), 5.34 - 5.47 (m), 5.49 (br. s.), 7.82 (br. s.).

**G2-AZDye405-S-Lactose (G2-Dye-S-Lac, TK-051)**

^1^H NMR (500 MHz, DMSO-d_6_): δ (ppm) = 1.10 (br. s.), 1.17 (br. s.), 1.24 (br. s.), 2.02 (br. s.), 2.63 (br. s.), 2.83 (br. s.), 3.05 (br. s.), 3.46 (br. s.), 3.53 (br. s.), 3.62 (br. s.), 3.75 (br. s.), 3.95 - 4.16 (m), 4.21 (br. s.), 4.37 (br. s.), 4.48 (br. s.), 4.53 (br. s.), 4.63 (br. s.), 4.69 (br. s.), 4.74 (br. s.), 5.06 (br. s.), 5.15 (br. s.), 7.81 (br. s.)

**G3-AZDye405-S-Mannose (G3-Dye-S-Man, TK-052)**

^1^H NMR (500 MHz, DMSO-d_6_): δ (ppm) = 1.09 (br. s.), 1.13 (br. s.), 1.24 (br. s.), 2.02 (br. s.), 2.63 (br. s.), 2.82 (br. s.), 3.39 (br. s.), 3.47 (br. s), 3.62 (br. s.), 4.11 (br. s.), 4.33 (br. s.), 4.38 (br. s.), 4.51 (br. s.), 4.59 (s), 4.66 (br. s.), 7.82 (br. s.).

**G3-AZDye405-S-Maltose (G3-Dye-S-Mal, TK-053)**

^1^H NMR (500 MHz, DMSO-d_6_): δ (ppm) = 1.10 (br. s.), 1.13 (br. s.), 1.24 (br. s.), 2.02 (br. s.), 2.63 (br. s.), 2.82 (br. s.), 3.07 (br. s.), 3.25 (br. s.), 3.36 - 3.53 (m), 3.54 - 3.67 (m), 3.66 - 3.81 (m), 3.97 - 4.15 (m), 4.18 (d, J=5.00 Hz), 4.37 (br. s.), 4.48 (br. s.), 4.85 (d, J=11.03 Hz), 5.02 (br. s.), 5.14 (d, J=4.00 Hz), 5.39 (d, J=5.20 Hz), 5.49 (br. s.), 7.82 (br. s.).

**G3-AZDye405-S-Lactose (G3-Dye-S-Lac, TK-054)**

^1^H NMR (500 MHz, DMSO-d_6_): δ (ppm) = 1.10 (br. s.), 1.13 (br. s.), 1.24 (br. s.), 2.02 (br. s.), 2.63 (br. s.), 2.82 (br. s.), 3.06 (br. s.), 3.46 (br. s.), 3.53 (br. s.), 3.63 (br. s.), 3.75 (br. s), 3.97 - 4.17 (m), 4.21 (br. s.), 4.37 (br. s.), 4.48 (br. s.), 4.53 (br. s.), 4.63 (br. s.), 4.69 (br. s.), 4.74 (br. s.), 5.06 (br. s.), 5.15 (br. s.), 7.82 (br. s.).

**Synthesis of Pseudo-glycodendrimer G2-Spermine(BBB)-AZDye405-S-Maltose**

The reaction was completely carried out in baked-out Schlenk tube under inert gas. G2-Alkyne (82.4 mg, 1.0 eq.) was dissolved in dry DMSO (2 mL). Then CuI (7.83 mg, 0.1 eq.) and DIPEA (5.9 mg, 8 µL, 0.1 eq. calculated on the alkyne-groups of G2-alkyne) were added. In a second baked-out Schlenk tube under argon atmosphere AZ-Dye-405 (3.5 mg) was dissolved in dry DMSO (0.35 mL) to get a concentration of 1 mg per 100 µL for the dye molecule. Then 340 µL (2.0 eq.) of the AZ-Dye-405 solution was added to the pseudo-dendrimer solution under protection atmosphere. The resulting reaction solution was stirred for 24 h at rt. Then Spermine(N_3_BBB) (3.14 mg, 2.0 eq.) was added and the resulting reaction solution was stirred for 24 h at rt. After 1 day of stirring β-D-Maltose-propylazide (192.6 mg, 1.1 eq. calculated on the alkyne-groups) was added to the reaction mixture. The reaction was additionally stirred for 3 days at rt. After that, the mixture was quenched with water and purified by dialysis (2000 Da RC membrane) over 2 days, changing the water 3 times per day. The aqueous product solution was freeze-dried to obtain the product as a white powder (205 mg, yield: 86 %).

GPC: M_n_ = 92400 g/mol, Ð = 1.66, dn/dc = 0.093. ^1^H NMR (500 MHz, DMSO-*d*_6_) δ (ppm) = 1.10 (br. s.), 1.17 (br. s.), 1.24 (br. s.), 1.35 (br. s.), 2.02 (br. s.), 2.63 (br. s.), 2.83 (br. s.), 3.07 (br. s.), 3.24 (br. s.), 3.35 - 3.40 (m), 3.41 - 3.53 (m), 3.54 - 3.66 (m), 3.67 - 3.81 (m), 4.11 (br. s.), 4.18 (d, *J*=6.31 Hz), 4.37 (br. s.), 4.48 (d, *J*=2.84 Hz), 4.82 - 4.92 (m), 5.02 (br. s.), 5.14 (d, *J*=3.47 Hz), 5.40 (d, *J*=5.67 Hz), 5.50 (br. s.), 7.81 (br. s.).

**Synthesis of deprotected Pseudo-glycodendrimer G2-Spermine-AZDye405-S-Maltose Synthesis of deprotected Pseudo-glycodendrimer G2-Spermine-AZDye405-S-Maltose**

For deprotection of the Boc-groups on the Spermine 2.3 M HCl was used. In a 5 ml round-bottom flak G2-Spermine(BBB)-AZDye405-S-Maltose (30.2 mg) was dissolved in 2.3 M HCl (0.7 mL). The reaction solution was stirred for 4 h at rt. Then the reaction solution was quenched with water. The aqueous crude product was purified by dialysis (2000 Dalton RC membrane) over 1 day, changing the water 3 times per day. After the dialysis the G2-Spermine-AZDye405-S-Maltose solution was treated with saturated NaHCO_3_ solution to neutralize the solution. Then a second dialysis (2000 Dalton RC membrane) was carried out over 1 day, changing the water 3 times per day. After freeze-drying the product was obtained as a white powder (25.3 mg, yield: 84 %).

^1^H NMR (500 MHz, DMSO-*d*_6_) δ (ppm) = 1.01 (br. s), 1.11 (br. s), 1.16 (br. s.), 1.24 (br. s.), 1.93 - 2.10 (m), 2.63 (br. s.), 2.84 (br. s.), 3.01 - 3.14 (m), 3.37 - 3.52 (m), 3.54 - 3.65 (m), 3.67 - 3.86 (m), 3.98 - 4.26 (m), 4.30 - 4.44 (m), 4.49 (br. s.), 4.91 (br. s), 4.98 - 5.11 (m), 5.19 (br. s.), 5.36 - 5.69 (m), 7.82 (br. s.).

**Synthesis of Pseudo-glycodendrimer G2-TAT-AZDye405-S-Mannose**

The reaction was completely carried out in baked-out Schlenk tube under inert gas. G2-Alkyne (80.0 mg, 1.0 eq.) was dissolved in dry DMSO (2 mL). Then CuI (7.65 mg, 0.1 eq.) and DIPEA (5.2 mg, 7 uL, 0.1 eq. calculated on the alkyne-groups of G2-alkyne) were added. In a second baked-out Schlenk tube under argon atmosphere AZ-Dye-405 (4.1 mg) was dissolved in dry DMSO (0.41 mL) to get a concentration of 1 mg per 100 µL for the dye molecule. Then 330 µL (2.0 eq.) of the AZ-Dye-405 solution was added to the pseudo-dendrimer solution. The resulting reaction solution was stirred for 24 h at rt. Then TAT-azide (3.77 mg, 2.0 eq.) was added and the reaction solution was stirred for 24 h at rt. After 1 day stirring α-D-Mannose-propylazide (191.0 mg, 1.1 eq. calculated on the alkyne-groups) was added to the reaction mixture. The reaction solution was additionally stirred for 3 days at rt. After that, the mixture was quenched with water and purified by dialysis (2000 Da RC membrane) over 2 days, changing the water 3 times per day. The aqueous product solution was freeze-dried to obtain the product as a white powder (151 mg, yield: 78 %).

^1^H NMR (500 MHz, DMSO-d6) δ (ppm) = 1.09 (br. s.), 1.17 (br. s.), 1.24 (br. s.), 2.02 (br. s.), 2.63 (br. s), 2.83 (br. s.), 3.39 (d, J=4.10 Hz), 3.47 (s), 3.61 (br. s.), 4.10 (br. s.), 4.30 (br. s), 4.39 (br. s), 4.51 (br. s), 4.59 (s), 4.67 (br. s), 7.82 (br. s.).

**Analysis of protons in ^1^H-NMR of sugar-azide**

The assignment of the protons in the ^1^H NMR of the sugar molecules was carried out using different references. On the one hand, own spectra of the starting compounds were recorded. Databases for similar structures and published data were also used. Finally, all assignments were compared with a simulation of the spectrum.

1. **Calculation of different parameters to characterize pseudo-dendrimers and pseudo-glycodendrimers**

**3.1 Degree of branching**

$${DB}_{Frechet}=\frac{D+T}{D+T+L}$$

$${DB}_{Frey}=\frac{2D}{2D+L}$$

**Table S5.** Calculation of the degree of branching from the integral intensity of ^1^H NMR.

|  | G0-OH | G1-OH | G2-OH | G3-OH |
| --- | --- | --- | --- | --- |
| Integral terminal | 3.01 (26 %) | 3.00 (54 %) | 3.00 (60 %) | 3.00 (44 %) |
| Integral linear | 6.25 (54 %) | 0.26 (6 %) | 0.21 (4 %) | 1.51 (22 %) |
| Integral dendritic | 2.29 (20 %) | 2.76 (41 %) | 2.46 (36 %) | 2.36 (34 %) |
| DB Fréchet | 0.459 | 0.957 | 0.963 | 0.791 |
| DB Frey | 0.423 | 0.955 | 0.959 | 0.791 |

The degree of branching was calculated from the ^1^H-NMR of PD. The ratio of the integral intensity of terminal, linear and dendritic groups was used for this purpose. The percentage of the individual groups was also calculated from this. For the basic structure G0-OH, 54 % linear groups, 26 % terminal and 20 % dendritic groups were calculated. The structure has more than twice as many linear groups as terminal or dendritic groups. Together with SAXS measurements, we assume that the core macromolecule, G0-OH, is a linear polymer chain with integrated dendritic units, possessing terminal units as pending groups. This is the reason for the higher proportion of linear groups relative to terminal or dendritic groups and the slight excess of terminal to dendritic groups. Further details in the paper (Chapter 3.3.3).

**3.2** **Calculation of OH groups for hydroxy-functionalized pseudo-dendrimers (G0-OH - G3-OH)**

Calculation of OH groups is based on ^1^H NMR and SEC. First, the terminal, dendritic and linear signals were assigned in the NMR spectra. The ratio of the integral intensity between the terminal, dendritic and linear units was determined. The molecular weight of the structures and the proportions of the groups can be used to determine the weight ratio. This is then divided by the respective molecular weight of the individual units to calculate the number. For each dendritic unit there is one free OH group and for the terminal there are two OH groups and one for the focal group. This procedure is used to calculate the OH groups for G0-OH, G1-OH, G2-OH and G3-OH.

**Table S6.** Calculation for number of free OH groups for the basic structure G0-OH.

| Free OH-groups overall | 42 + one OH from focal unit | | | |
| --- | --- | --- | --- | --- |
| Number of free OH groups per unit | 0 | 22 | 20 |  |
| Free OH group per monomer unit D, L, T | 0 | 1 | 2 |  |
| Number of monomer units from D, L, T | 8 | 22 | 10 |  |
| Group | D | L | T |  |
| Molar mass for one monomer unit | 115.12 g/mol | 116.12 g/mol | 117.12 g/mol |  |
| Ratio of molar mass for each group | 933 g/mol | 2545 g/mol | 1222 g/mol |  |
| Molar mass dendrimer from GPC | 4700 g/mol | | | |
| Calculated ratio | 0.198 | 0.542 | 0.260 |  |
| Absolut integral value  from NMR | 2.29 | 6.25 | 3.00 | 11.54 |
| Units | Dendritic | Linear | Terminal | overall |

$calculated ratio=\frac{absolute integral value of the unit}{overall value}$ for terminal unit: $0.260= \frac{3.00}{11.54}$

$ratio of molar mass=molar mass \times calculated ratio$

for terminal unit: $1222 \frac{g}{mol}=4700 \frac{g}{mol} \times0.260$

$number of units=\frac{ratio ofmolar mass}{molar mass of one unit}$ for terminal unit: $10=\frac{1222 \frac{g}{mol}}{117.12 \frac{g}{mol}}$

$free OH groups=linear units+2\times terminal units+focal unit$

OH groups of G0-OH: $43=22+2\times10+1$

**Table S7.** M_n_, M_w_, Đ, number of OH groups, and degree of branching (DB) of G0-OH, G2-OH, and G3-OH.

| **units** | **G0-OH** | **G1-OH** | **G2-OH** | **G3-OH** |
| --- | --- | --- | --- | --- |
| M_n, SEC_ | 4700 | 18300 | 19000 | 32100 |
| M_w, SEC_ | 8900 | 25100 | 28200 | 49000 |
| Đ_SEC_ | 1.88 | 1.36 | 1.49 | 1.52 |
| OH groups^a^ | 43 | 177 | 204 | 301 |
| DB_Fréchet_ | 0.459 | 0.957 | 0.963 | 0.791 |
| ^a^Determination by ^1^H NMR and DB. | | | | |

**3.3 Calculation of sugar groups for pseudo-glycodendrimers**

To calculate the number of sugar groups, the molecular weight of the pseudo-dendrimer and the number of OH groups are required. First the molecular weight of the alkyne functionalized pseudo-dendrimer was calculated. The number of alkyne groups is the same as the number of OH groups in the pseudo-dendrimer because the functionalization of OH to alkyne is a known parameter. The alkyne functionalized pseudo-dendrimer cannot be measured by SEC, because it is not soluble in the solvents which are used in SEC measurements.

**Table S8.** Data from second generation pseudo-dendrimer G2-OH, for example, to calculate the molecular weight of sugar functionalized pseudo-glycodendrimers.

|  | M in g/mol | Number of OH groups |
| --- | --- | --- |
| G2-OH | 19,000 | 204 |
| Alkyne group | 98 |  |

$$M_{pseudodendrimer with alkyne groups}=M_{pseudodendrimer with OH groups}+ number OH groups\times M_{alkyne unit}$$

$$35300\frac{g}{mol}=19000\frac{g}{mol}+204 \times98\frac{g}{mol}$$

The ratio between the proton (CH) of the triazole cycle and the protons of the CH_2_ group in the alkyne linker was determined in the ^1^H NMR spectra. With this ratio the DoF in percent can be obtained and multiplied with the number of OH/alkyne groups of the pseudo-dendrimer. With the molecular weight of pseudo-dendrimer with alkyne groups and the number and molar mass of the sugar units the theoretical molecular weight of the pseudo-glycodendrimer was obtained.

| G2-S-Mannose |  |
| --- | --- |
| DoF from NMR | 97 % |
| Alkyne groups | 204 |
| M_Mannoseazide_ | 263.45 g/mol |

$number of sugar units=number alkyne groups \times DoF$ for G2-S-Mannose: $198=204 \times97\%$

$$M_{pseudo-glycodendrimer}= M_{pseudodendrimer with alkyne groups}+number of sugar units \times M_{sugar azide}$$

$$87500 \frac{g}{mol}= 35300 \frac{g}{mol}+198 \times263.45 \frac{g}{mol}$$

1. Optimization of click reaction for synthesizing pseudo-glycodendrimers

**Table S9.** Efficiency of improved click reaction for the synthesis of G2-S-Man and G3-S-Man compared to previously published^a^ reaction conditions.

|  | G2-S-Man^a^ | | G3-S-Man^a^ | | G2-S-Man^b,c^ | | G2-S-Man^b,c^ | G2-S-Man^d^ | G3-S-Man^d^ |
| --- | --- | --- | --- | --- | --- | --- | --- | --- | --- |
| PGD (kg/mol) | | 41 | | 69 | | n.d. first step^e^ | 42 second step^e^ | 76 | 115 |
| GX-OH (X = 2 or 3)  OH groups | | 138 | | 196 | | 213 | 213 | 204 | 301 |
| PGD Man groups | | 90 | | 168 | | 85 | 194 | 198 | 292 |
| DoF | | 65 %^f^ | | 84 % | | 40 % | 91 % | 97 % | 97 % |
| Reaction conditions | | 2d, 45°C, CuSO_4_^.^5H_2_O, Na ascorbate, THF:water (1:1) | | | | 8d, 45°C, CuSO_4_^.^5H_2_O, Na ascorbate, THF:water (1:1) | | 3d, rt, CuI, DIPEA, DMSO | |

^a^published data from Firdaus et al. (2023).

^b^used previously published conditions (Firdaus et al., 2023) for click reaction.

^c^direct use of 7d for **G3-S-Man** with previously published conditions, achieving 88% of Man decoration in **G3-S-Man**.

^d^using improved click reaction conditions.

^e^first step: applied click reaction for 3d; second step: repeating click reaction of partially converted **G2-Alkyne** with S-Man for 5d again.

^f^recalculated value (Firdaus et al., 2023).

1. **Synthesis and characterization of pseudo-dendrimers and pseudo-glycodendrimers: Optimized synthetic steps**

**5.1 Optimized synthetic pathway of Pseudo-dendrimers**

The synthesis of 2^nd^ and 3^rd^ generation of bis-MPA-based pseudo-dendrimers (PD) (**Figure 1a**: **G2-OH** and **G3-OH**) for the final **PGD** is characterized by following steps: (i) Acid-catalyzed polycondensation of Bis-MPA resulting in the formation of the core macromolecule G0-OH; (ii) iterative steps of esterification of acetonide-protected bis-MPA anhydride (**Figure S6**) and their deprotection of chemically conjugated bis-MPA to generate corresponding 2^nd^ and 3^rd^ generation of **PD** on the core macromolecule **G0-OH**. These synthetic steps are well-known from previous studies (Firdaus et al., 2018, 2023). Details on the complete synthetic pathways are presented in the SI (**Figure S1**).

In order to introduce efficiently the requested 2^nd^ and 3^rd^ dendrons in **G2-Sugar** and **G3-Sugar** (**Figure 1b**), and thus, to introduce a high number of terminal sugar units in **PGD** for their biological actions, the number of OH groups in **G0-OH** has to be known and should be high. **G0-OH** has a molecular weight of 4700 g/mol with 43 OH surface groups determined by SEC and NMR study (**Table S7**, **Figure S8**). The relatively high molecular weight and number of functional terminal groups after one reaction step cannot be reached with the synthesis of 1^st^ or 2^nd^ generation bis-MPA-based dendrimers (Firdaus et al., 2023).

The esterification of **G0-OH** with bis-MPA acetonide anhydride results in the synthesis of **G1-acetonide** as intermediate compound for the final synthesis of **G1-OH** (**Figure S1**). The key step of this synthesis is the complete separation of the solvent pyridine from **G1-Acetonide** (**Figure S1)**. For that the reaction mixture is treated with 2 % hydrochloric acid (HCl) to salt out the pyridine as pyridinium chloride. The pyridinium chloride passes into the aqueous phase, while the product **G1-Acetonide** is not soluble in water and remains in the organic dichloromethane (DCM). With this simple step, **G1-Acetonide** is obtained in high purity. For the deprotection of **GX-Acetonide** (X = 1, 2 or 3) into **GX-OH** (X = 1, 2 or 3), the reaction time for this deprotection step is extended to overnight in order to avoid any residual acetonide groups. With these small changes to the known reaction conditions from Firdaus et al., (2023), **G2-OH** and **G3-OH** can finally be used as building blocks for the final synthesis of sugar-decorated **PD (Figure 1b)**.

**5.2** **Azido functionalization of different sugars for final click reaction to realize pseudo-glycodendrimers**

In order to obtain a broader overview of the influence of the sugar decoration of **PGD** on biomedical applications, different types of azido-functionalized sugars were synthesized and characterized. The sugars used are α‑D‑mannose, β‑D‑maltose and β‑D‑lactose. Acetate-protected sugars are selected as the starting material. This enables to carry out a directed functionalization of azido propyl group at the anomeric carbon of each sugar. Further details of synthesized S-Man, S-Mal, and S-Lac (**Figure 1b**) are presented in the Supporting Information (SI) (**Figures S2, S3**, **and S24-S29**; experimental part in SI).

**5.3** **New Click-Reaction conditions for a higher functionalization degree on sugar units in pseudo-glycodendrimers**

To realize requested 2^nd^ and 3^rd^ generation **PGD**, **G2-Sugar** and **G3-Sugar** (**Figure 1b**), first the reaction conditions for the sequential introduction of alkyne groups (**G2-Alkyne** and **G3-Alkyne**) and sugar groups (**G2-Sugar** and **G3-Sugar**) on the surface of **G2-OH** and **G3-OH** (**Figure 1**), reported by Firdaus et al. (2023), are used. Details of smoothly synthesized **G2-Alkyne** and **G3-Alkyne** are presented in the SI (**Figures S21 and S23**; experimental part in SI). The final click reaction of **G2-Alkyne** and **G3-Alkyne** with S-Man is carried out with a copper(II) sulfate as catalyst and sodium ascorbate as reducing agent in a 1:1 solvent mixture of tetrahydrofuran and water for 2-7 days at 40°C (Firdaus et al., 2023). Due to a limited solubility of **G2-Alkyne** in the reaction mixture, a degree of Man functionalization up to only 41 % on the surface of PD with alkyne groups is achieved (**Figure 1b**) for the establishment of **G2-S-Man** (SI; **Table S9**).

For the use of PGD as therapeutic systems, a nearly perfect dense sugar shell is a prerequisite. This not only ensures targeted biological interactions, but also helps to minimize undesired interactions caused by the hydrophobic nature of the dendritic polyester scaffold, thereby enhancing the specificity and efficacy of PGDs in biological applications. To optimize the copper salt catalyzed click reaction for the chemical attachment of spacered sugars (**Figure S3**: S-Man, S-Lac, and S-Man) on the surface of **G2-Alkyne** and **G3-Alkyne** (**Figure 1b**), the following issues are changed for this final conversion step: (i) Dimethyl sulfoxide (DMSO) is used to properly dissolve all the starting materials; and (ii) the copper(II) species with the reducing agent is exchanged by the use of copper(I) iodide. This enables to run the reaction at room temperature for 3 days under protection atmosphere and to obtain the products **G2-Sugar** and **G3-Sugar** with a degree of functionalization ≥ 94 % (**Table 1**) after intense 2 days dialysis in water (**Figure 1b**).

**Figure S4.** ^1^H-NMR spectra of **G2-S-Mal** in DMSO-d_6_ after 24, 48, and 72 h. Signals of triazole and alkyne groups used for the calculation of degree of functionalization with S-Mal units for **G2-S-Mal**. Simplified structure of **G2-S-Mal** compared to structure of **G2-S-Mal** in **Figure S35**. Details of calculation in SI.

In general, the degree of functionalization (DoF) for **PGD** was determined by ^1^H-NMR spectroscopy in DMSO-d_6_. **Figure S4** exemplifies the successful conversion of **G2-Alkyne** into **G2-S-Mal**, showing the optimization of the reaction time at room temperature. After 24 h, 48 h and 72 h a sample is taken, deactivated with water, purified by dialysis, and investigated by ^1^H NMR spectroscopy. In the ^1^H-NMR spectra at 7.82 ppm the proton from the triazole ring is assigned. This indicates the attachment of S-Mal to the surface of **PD** **G2-Alkyne**. For the determination of the DoF the alkyne signal (CH_2_-group) at 2.36 ppm is used (**Figure S4**) to calculate the percentage of converted triple bonds. At 24 h and 48 h the DoF is 95 % for **G2-S-Mal**. The best result is achieved after 72 hours with 98 % of sugar attachment on **PD**, **G2-Alkyne**. **Table S9** summarizes the enhanced attachment of azido-functionalized S-Man on the surface of **G2-Alkyne** and **G3-Alkyne** compared to previously used method in the former study (Firdaus et al., 2023). This results in a nearly complete conversion of OH groups into sugar groups with a high yield of 97 % in both converted **PD**, **G2-Alkyne** and **G3-Alkyne**, into **PGD**, which implies a high efficiency for the improved click reaction. Further details on the characterization of **G2-sugar** and **G3-sugar** and precursors (**G2-OH**, **G3-OH**, **G2-Alkyne**, and **G3-Alkyne**) are presented under point **3.3** and SI.

1. **Additional figures and tables**

**6.1** **^1^H NMR spectra of hydroxy-functionalized pseudo-dendrimers (G0-O1 - G3-OH), including core macromolecule (G0-OH) and building blocks**

**
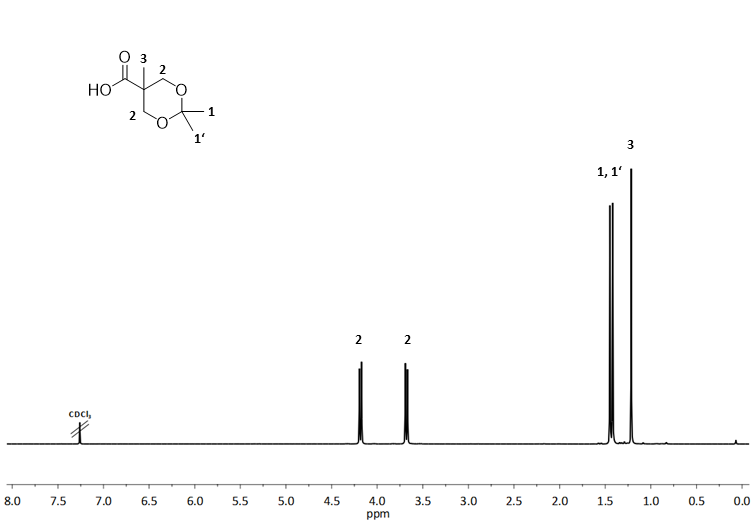
**

**Figure S5.** ^1^H NMR (500 MHz, CDCl_3_) of bis-MPA-acetonide.


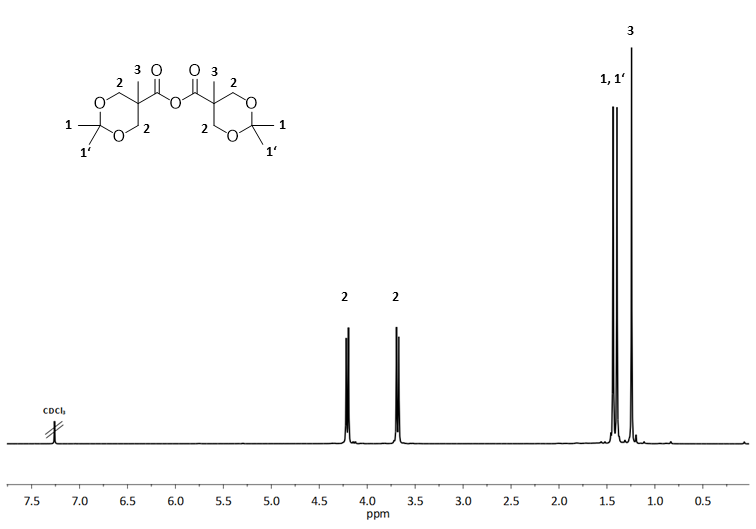


**Figure S6.** ^1^H NMR (500 MHz, CDCl_3_) of bis-MPA-acetonide-anhydride.


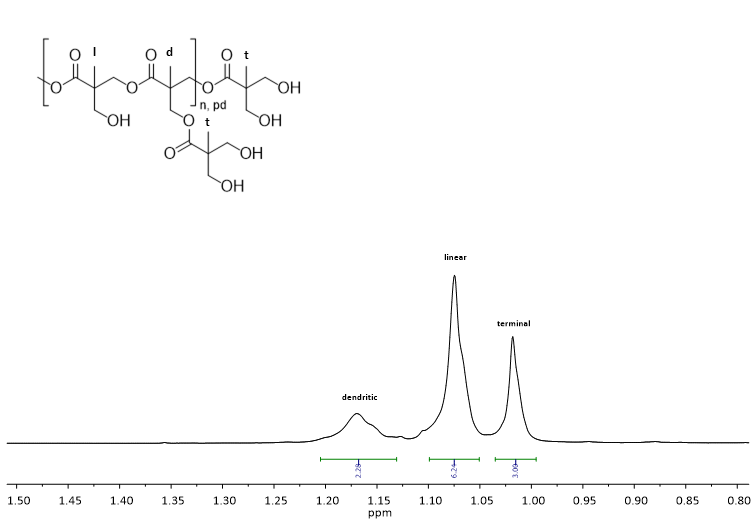


**Figure S7.** ^1^H NMR (500 MHz, DMSO-d_6_) of terminal, linear and dendritic signals of G0-OH.


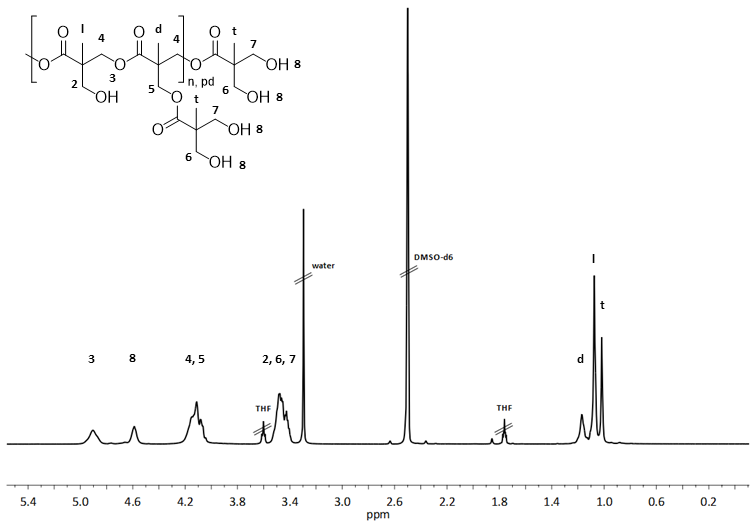


**Figure S8.** ^1^H NMR (500 MHz, CDCl_3_) of G0-OH.

**Figure S9.** ^1^H NMR (500 MHz, CDCl_3_) of G1-Acetonide.

**Figure S10.** ^1^H NMR (500 MHz, DMSO-d_6_) of G1-OH.

**Figure S11.** ^1^H NMR (500 MHz, DMSO-d_6_) of terminal, linear and dendritic signals of G1-OH.

**Figure S12.** ^1^H NMR (500 MHz, CDCl_3_) of G2-Acetonide with simplified structure.

**Figure S13.** ^1^H NMR (500 MHz, DMSO-d_6_) of G2-OH with simplified structure.

**Figure S14.** ^13^C NMR (125.75 MHz, DMSO-d_6_) of G2-OH.

^13^C NMR data are the same as previously reported by Firdaus et al. (2018).

**Figure S15.** ^1^H NMR (500 MHz, DMSO-d_6_) of terminal, linear and dendritic signals of G2-OH with simplified structure.

**Figure S16.** ^1^H NMR (500 MHz, CDCl_3_) of G3-Acetonide with simplified structure.


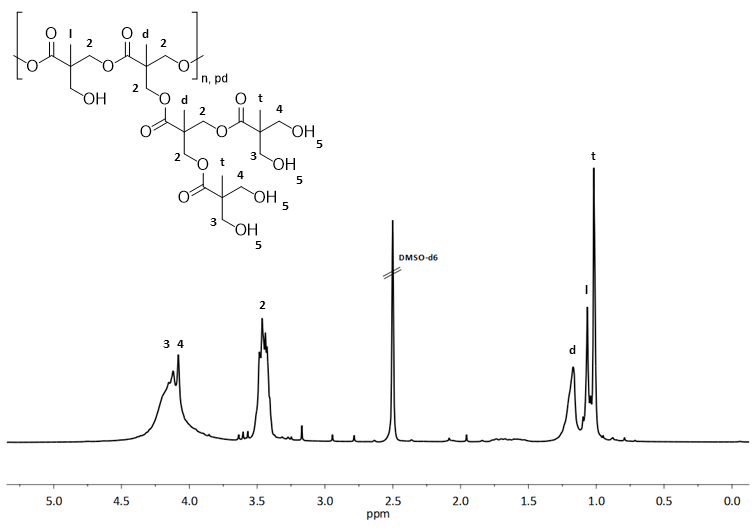


**Figure S17.** ^1^H NMR (500 MHz, DMSO-d_6_) of G3-OH with simplified structure.


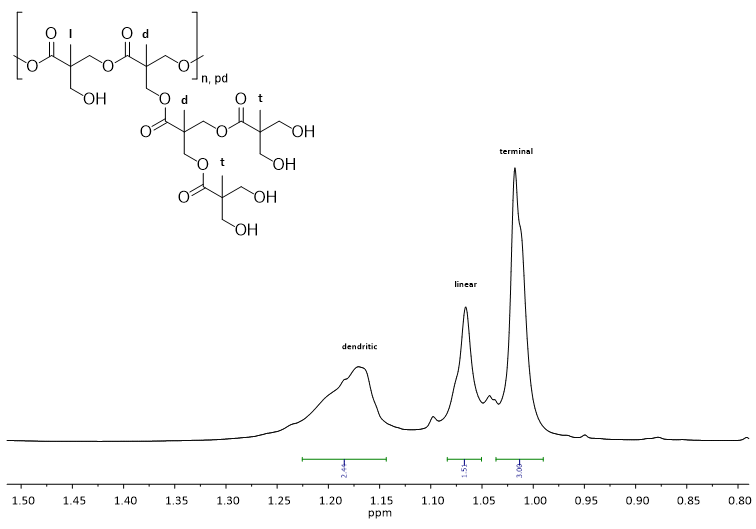


**Figure S18.** ^1^H NMR (500 MHz, DMSO-d_6_) of terminal, linear and dendritic signals of G3-OH with simplified structure.

**6.2 ^1^H NMR spectra of yne-functionalized pseudo-dendrimers (G0-Alkyne, G2-Alkyne, and G3-Alkyne), including building block**


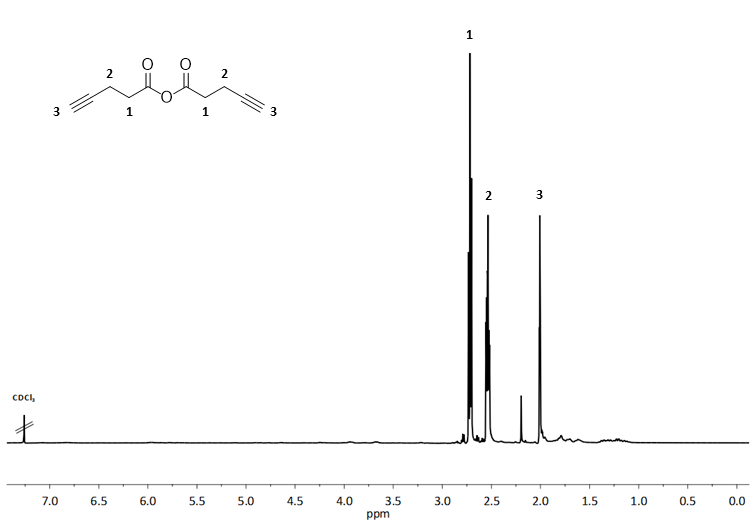


**Figure S19.** ^1^H NMR (500 MHz, CDCl_3_) of alkynyl-anhydride.


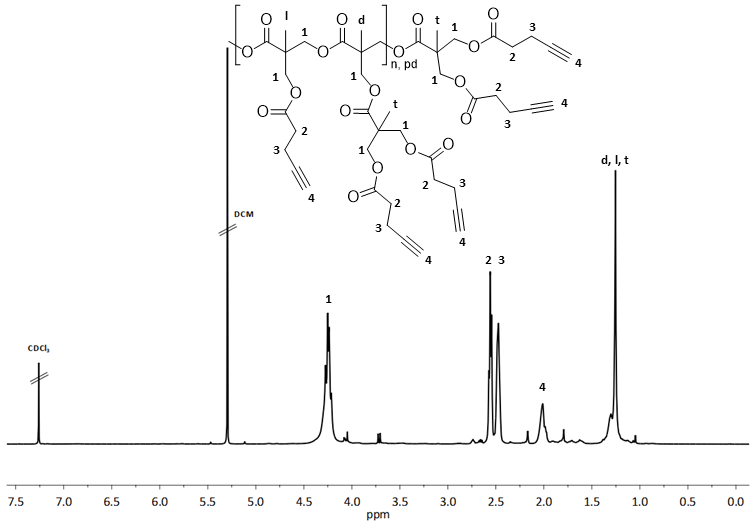


**Figure S20.** ^1^H NMR (500 MHz, CDCl_3_) of G0-Alkyne.


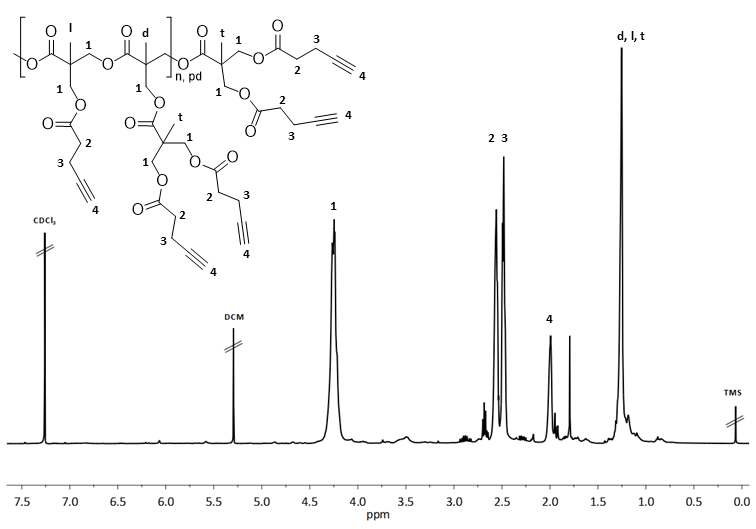


**Figure S21.** ^1^H NMR (500 MHz, CDCl_3_) of G2-Alkyne with simplified structure.

**Figure S22.** ^13^C NMR (125.75 MHz, CDCl_3_) of G2-Alkyne with simplified structure.

Signals with * belongs to the solvent THF. There are residual impurities from the conversion step which are not recognizable in the following ^13^C NMR spectra of **G2-S-Man** (**Figure S34**), **G2-S-Lac** (**Figure S37**) and **G3-S-Mal** (**Figure S40**).


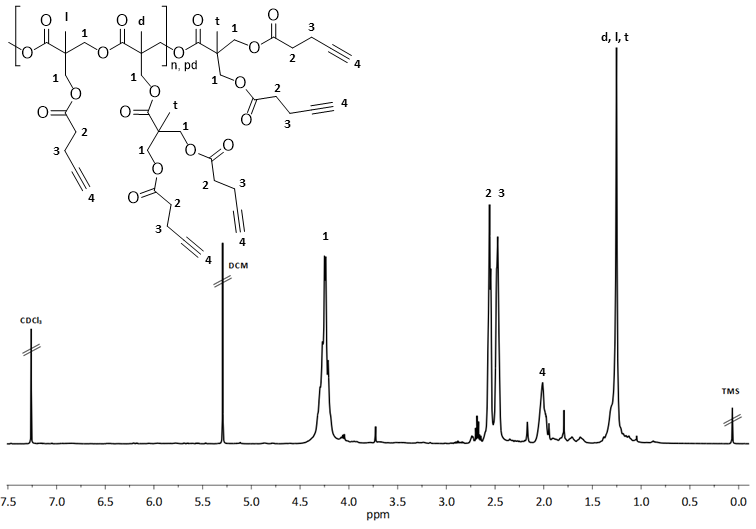


**Figure S23**. ^1^H NMR (500 MHz, CDCl_3_) of G3-Alkyne with simplified structure.

**6.3** **^1^H NMR spectra of sugar molecules**

**Figure S24.** ^1^H NMR (500 MHz, CDCl3) of α-D-Mannose-tetraacetate-propylazide.


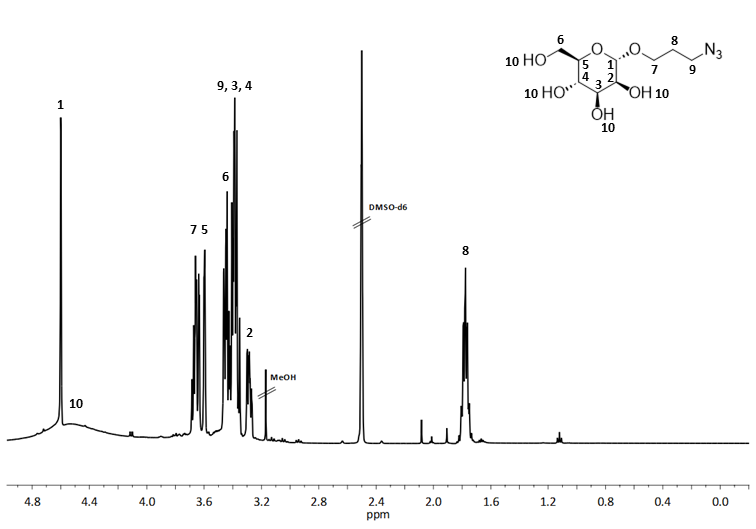


**Figure S25.** ^1^H NMR (500 MHz, DMSO-d_6_) of α-D-Mannose-propylazide.


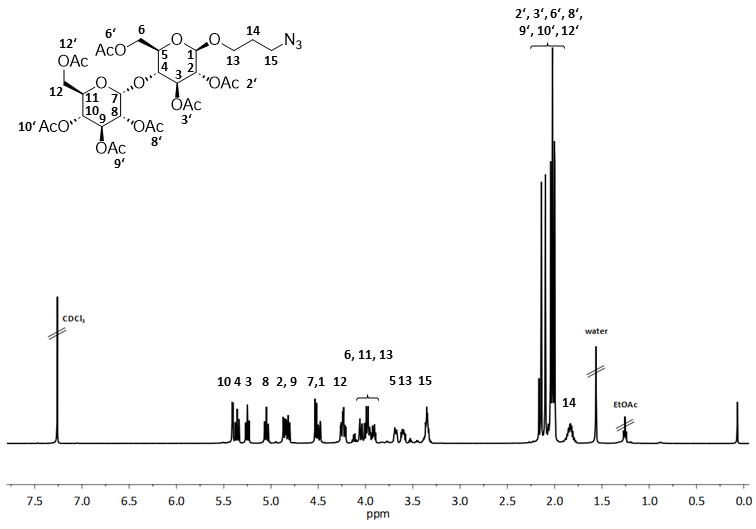


**Figure S26.** ^1^H NMR (500 MHz, CDCl_3_) of β-D-Maltose-heptaacetate-propylazide.


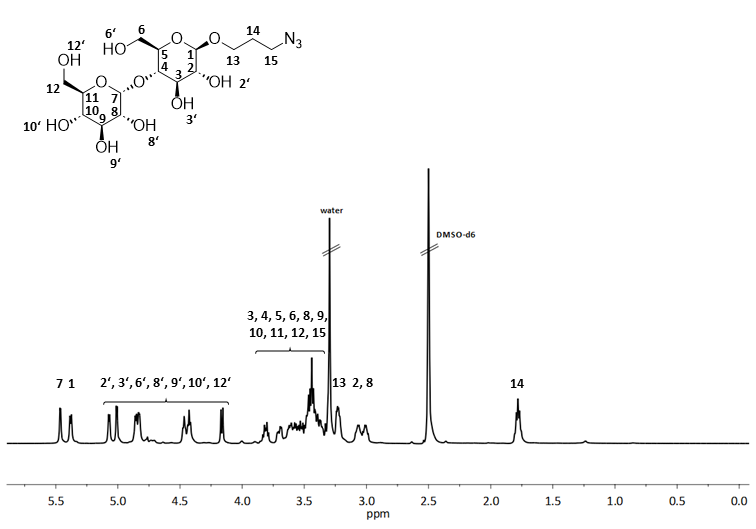


Figure S27. ^1^H NMR (500 MHz, DMSO-d_6_) of β-D-Maltose-propylazide.


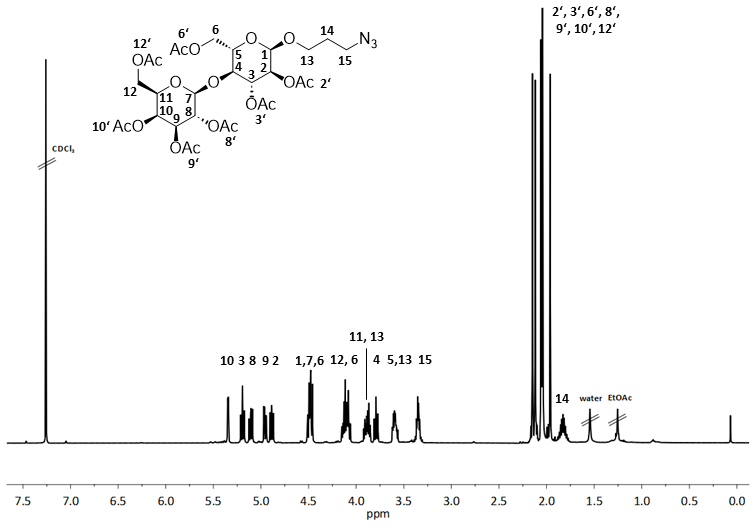


**Figure S28.** ^1^H NMR (500 MHz, CDCl_3_) of β-D-Lactose-heptaacetate-propylazide.


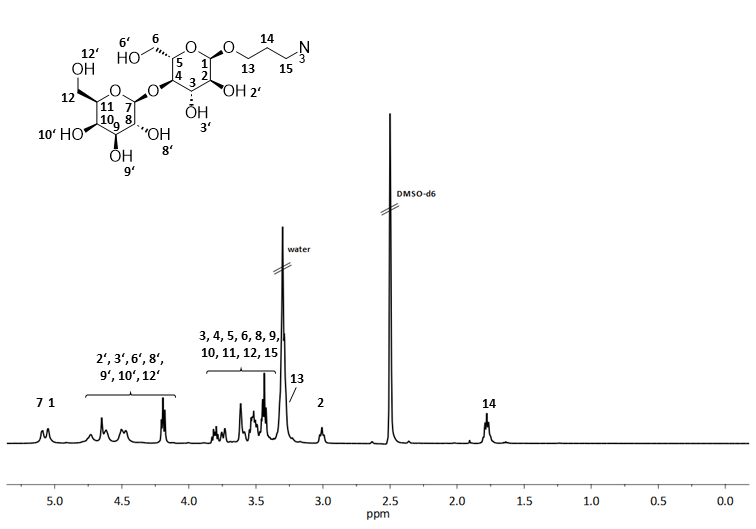


Figure S29. ^1^H NMR (500 MHz, DMSO-d_6_) of β-D-Lactose-propylazide.

**6.4** **^1^H NMR spectra of zero generation sugar-functionalized pseudo-dendrimers (G0-S-Man, G0-Dye-Alkyne, and G0-Dye-Man)**


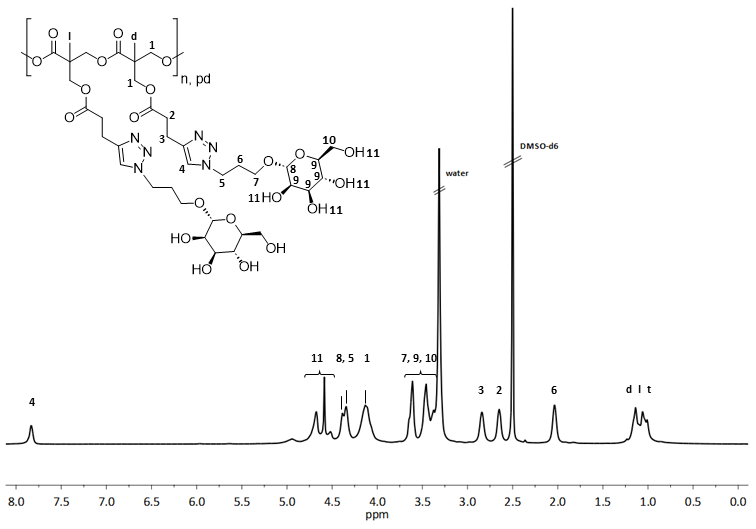


**Figure S30.** ^1^H NMR (500 MHz, DMSO-d_6_) of G0-S-Man. Simplified structure compared to G0-OH in **Figure S10**.


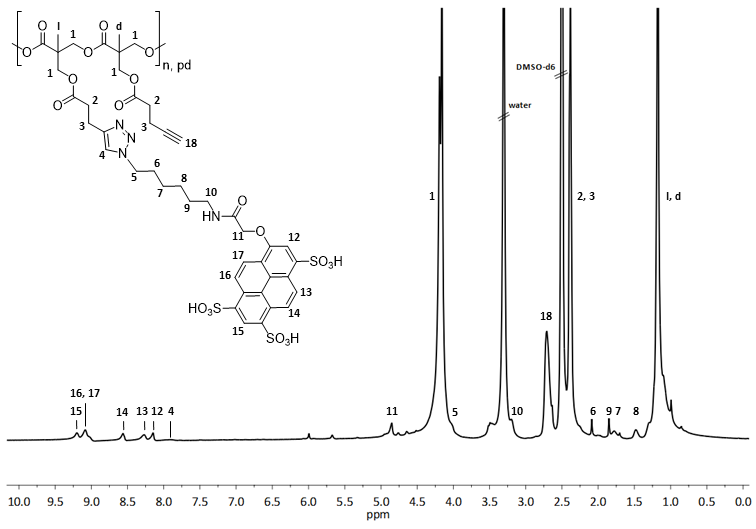


**Figure S31.** ^1^H NMR (500 MHz, DMSO-d_6_) of G0-Dye-Alkyne. Simplified structure compared to G0-OH in **Figure S10**.


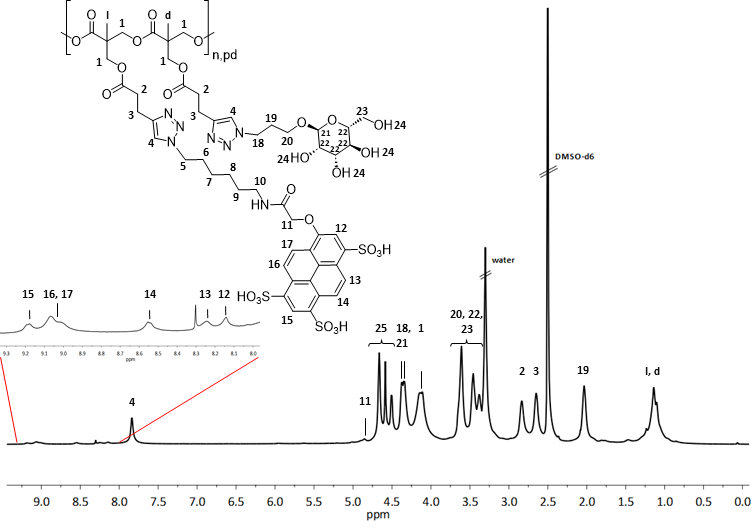


**Figure S32.** ^1^H NMR (500 MHz, DMSO-d_6_) of G0-Dye-S-Man. Simplified structure compared to G0-OH in **Figure S10**.

**6.5** **^1^H NMR spectra of 2^nd^ and 3^rd^ generation pseudo-glycodendrimers (G2-Sugar and G3-Sugar)**

**Figure S33.** ^1^H NMR (500 MHz, DMSO-d_6_) of G2-S-Man. Simplified structure with 2^nd^ generation dendron attached on linear unit compared to G2-OH in **Figure S13**.

**Figure S34.** ^13^C NMR (125.75 MHz, D_2_O) of **G2-S-Man**. Simplified structure with 2^nd^ generation dendron attached on linear unit compared to **G2-OH** in **Figure S13**.

^13^C NMR spectrum of **G2-S-Man** shows that the degree of branching at the ppm range from 48 ppm to 52 ppm as well as in the carbonyl range at 175 ppm cannot be calculated compared to **G2-OH** (Figure S15). Signal of part structure for 10 (Triazole N-CH_2_-CH_2_-R) in **G2-S-Man** overlaps with dendritic signal of 2. ^13^C NMR signals of 12 (Man-O-CH_2_-CH_2_-R) and 4 (CH_2_-O-COR) at about 67-68 ppm are assignable in the range of the mannose signals. Residual ^13^C NMR signals of **G2-S-Man** are assignable with different intensities.


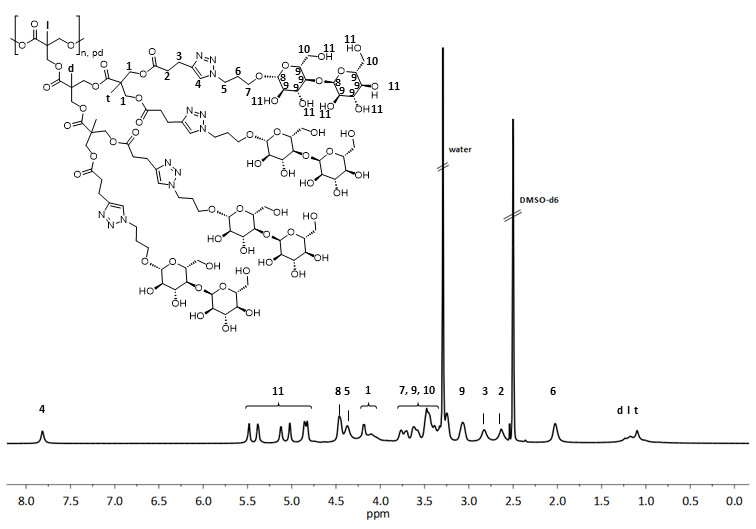


**Figure S35.** ^1^H NMR (500 MHz, DMSO-d_6_) of G2-S-Mal. Simplified structure with 2^nd^ generation dendron attached on linear unit compared to G2-OH in Figure S13.


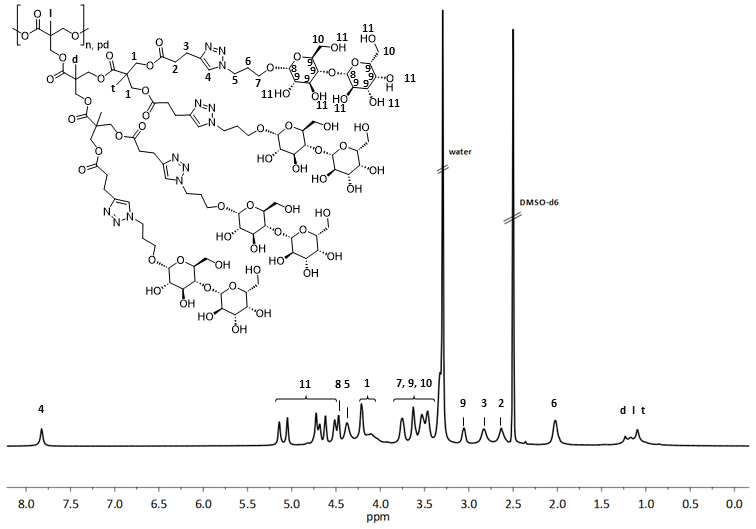


**Figure S36.** ^1^H NMR (500 MHz, DMSO-d_6_) of G2-S-Lac. Simplified structure with 2^nd^ generation dendron attached on linear unit compared to G2-OH in Figure S13.

**Figure S37.** ^13^C NMR (125.75 MHz, D_2_O) of **G2-S-Lac**. Simplified structure with 2^nd^ generation dendron attached on linear unit compared to **G2-OH** in **Figure S13**.

Degree of branching cannot be determined due to overlapping ^13^C NMR signals in the carbonyl (5) and quarternary C atom (2). All ^13^C NMR signals of **G2-S-Lac** are assignable.


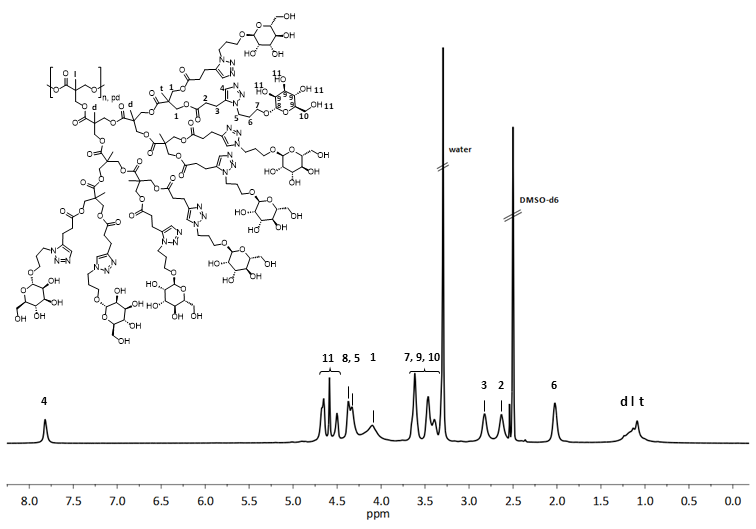


**Figure S38.** ^1^H NMR (500 MHz, DMSO-d_6_) of G3-S-Man. Simplified structure with 3^rd^ generation dendron attached on linear unit compared to G3-OH in **Figure S17**.


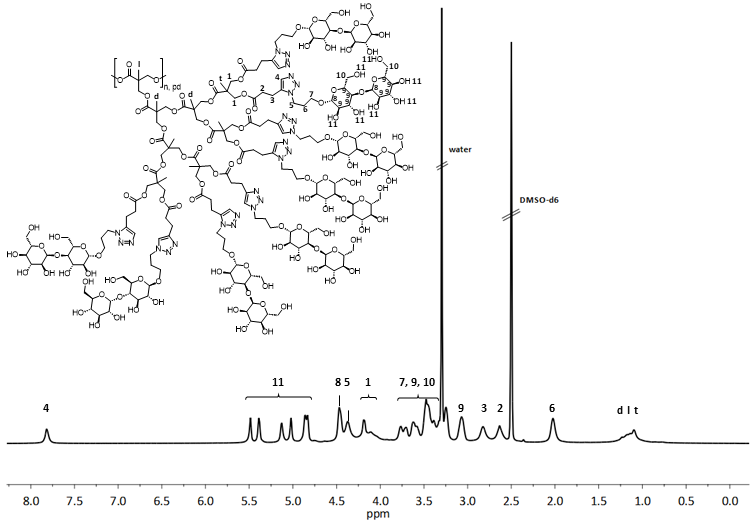


**Figure S39.** ^1^H NMR (500 MHz, DMSO-d_6_) of **G3-S-Mal**. Simplified structure with 3^rd^ generation dendron attached on linear unit compared to **G3-OH** in **Figure S17**.

**Figure S40.** ^13^C NMR (125.75 MHz, D_2_O) of **G3-S-Mal**. Simplified structure with 3^rd^ generation dendron attached on linear unit compared to **G3-OH** in **Figure S17.**

Degree of branching cannot be determined due to overlapping ^13^C NMR signals in the carbonyl (5) and quarternary C atom (2). All ^13^C NMR signals of **G3-S-Mal** are assignable.


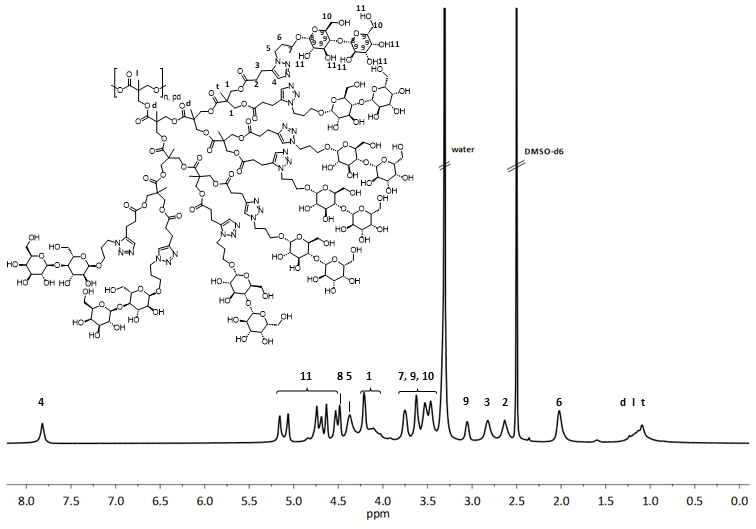


**Figure S41.** ^1^H NMR (500 MHz, DMSO-d_6_) of G3-S-Lac. Simplified structure with 3^rd^ generation dendron attached on linear unit compared to G3-OH in **Figure S16**.

**6.6 ^1^H NMR spectra of 2^nd^ and 3^rd^ generation dye-functionalized pseudo-glycodendrimers (G2-Dye-Sugar and G3-Dye-Sugar)**


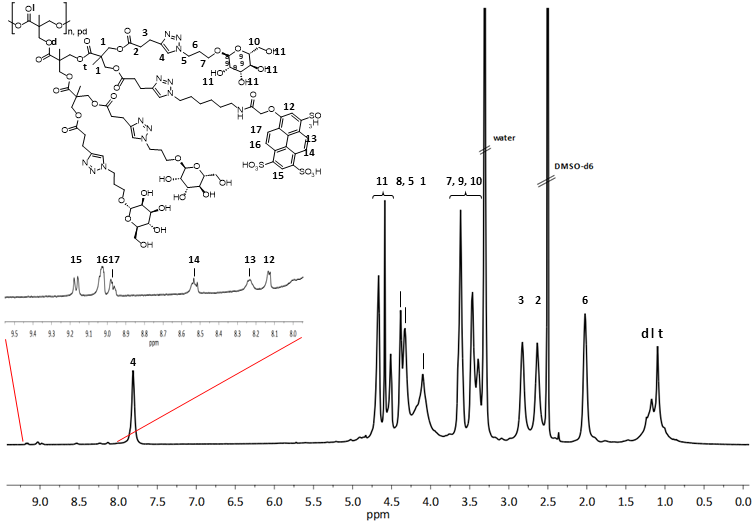


**Figure S42.** ^1^H NMR (500 MHz, DMSO-d_6_) of G2-Dye-S-Man. Simplified structure with 2^nd^ generation dendron attached on linear unit compared to G2-OH in **Figure S13**.


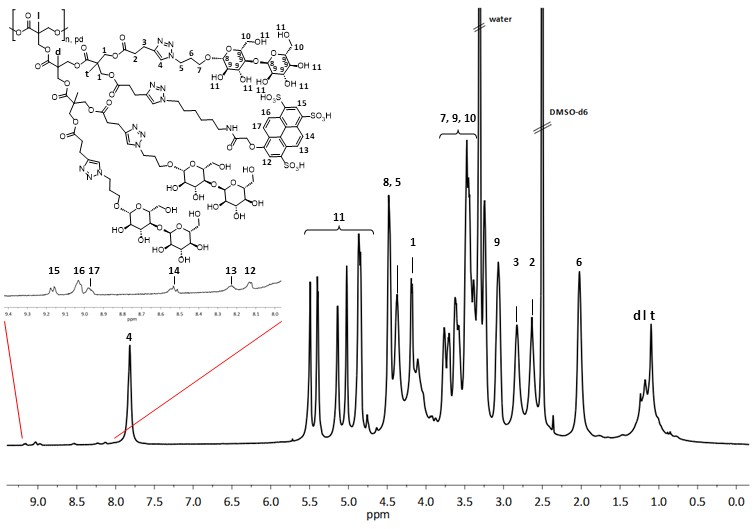


**Figure S43.** ^1^H NMR (500 MHz, DMSO-d_6_) of G2-Dye-S-Mal. Simplified structure with 2^nd^ generation dendron attached on linear unit compared to G2-OH in **Figure S13**.


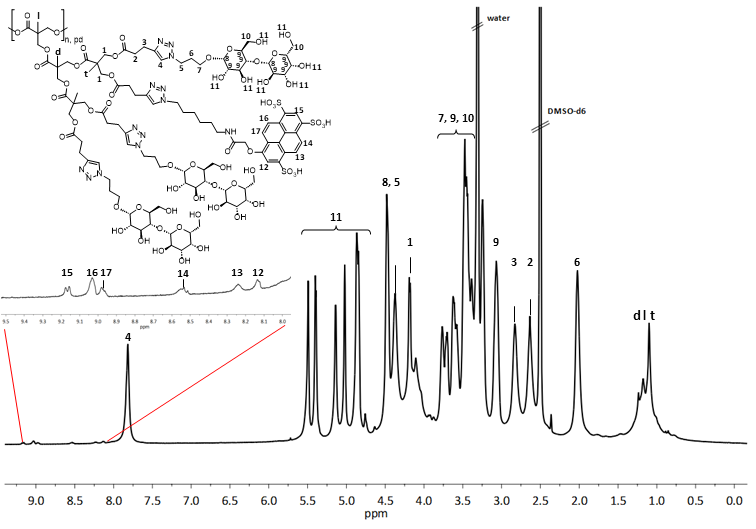


**Figure S44.** ^1^H NMR (500 MHz, DMSO-d_6_) of G2-Dye-S-Lac. Simplified structure with 2^nd^ generation dendron attached on linear unit compared to G2-OH in **Figure S13**.


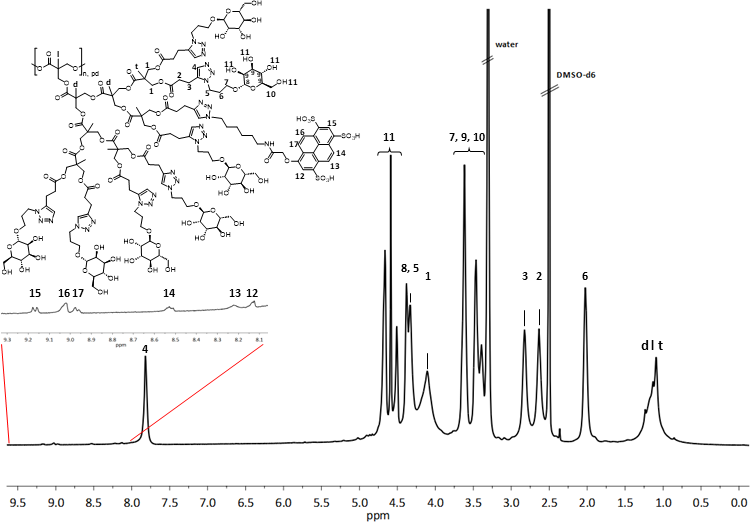


**Figure S45.** ^1^H NMR (500 MHz, DMSO-d_6_) of G3-Dye-S-Man. Simplified structure with 3^rd^ generation dendron attached on linear unit compared to G3-OH in **Figure S17**.


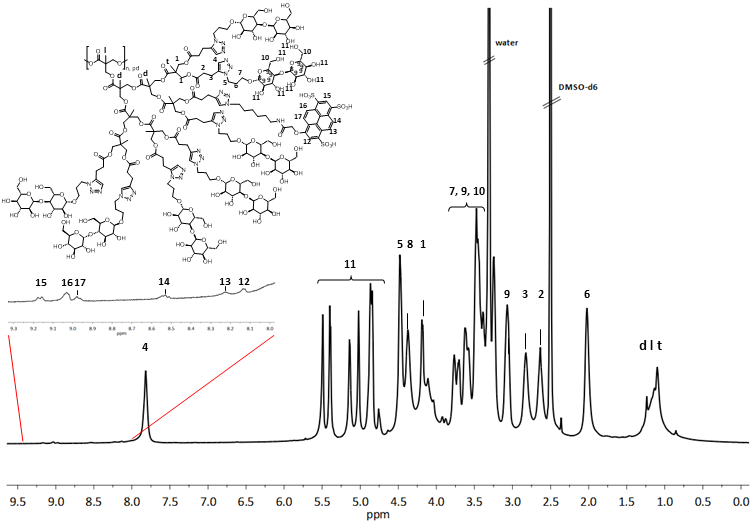


**Figure S46.** ^1^H NMR (500 MHz, DMSO-d_6_) of G3-Dye-S-Mal. Simplified structure with 3^rd^ generation dendron attached on linear unit compared to G3-OH in **Figure S17**.


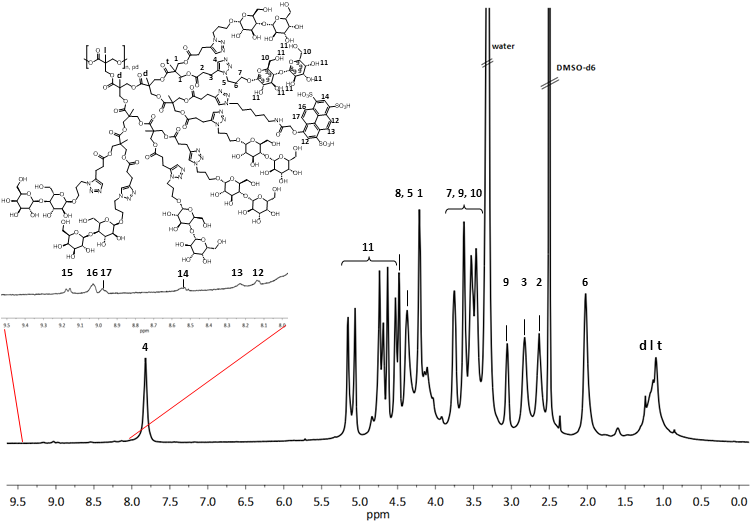


**Figure S47.** ^1^H NMR (500 MHz, DMSO-d_6_) of G3-Dye-S-Lac. Simplified structure with 3^rd^ generation dendron attached on linear unit compared to G3-OH in **Figure S17**.

**6.7 ^1^H NMR spectra of 2^nd^ generation oligoamine/peptide- and dye-functionalized pseudo-glycodendrimers**

**Figure S48.** ^1^H NMR (500 MHz, DMSO-d_6_) of G2-Spermine(BBB)-Dye-S-Mal. Simplified structure with 2^nd^ generation dendron attached on linear unit compared to G2-OH in **Figure S13**.


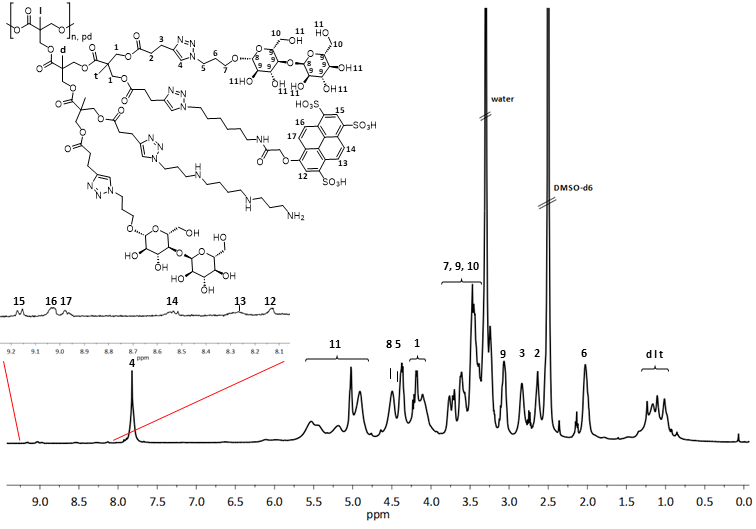


**Figure S49.** ^1^H NMR (500 MHz, DMSO-d_6_) of G2-Spermine-Dye-S-Mal. Simplified structure with 2^nd^ generation dendron attached on linear unit compared to G2-OH in **Figure S13**.


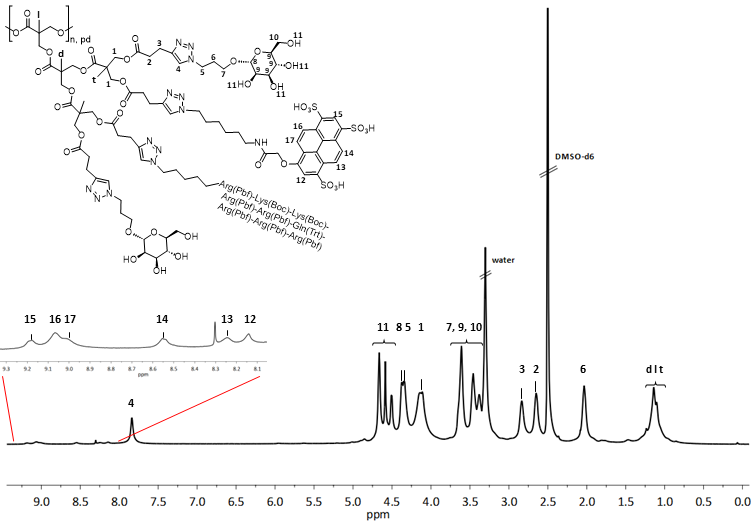


**Figure S50.** ^1^H NMR (500 MHz, DMSO-d_6_) of G2-TAT-Dye-S-Man. Simplified structure with 2^nd^ generation dendron attached on linear unit compared to G2-OH in **Figure S13**.

**6.8** **SEC chromatograms and molecular parameter of (dye-labeled) of pseudo-glycodendrimers**


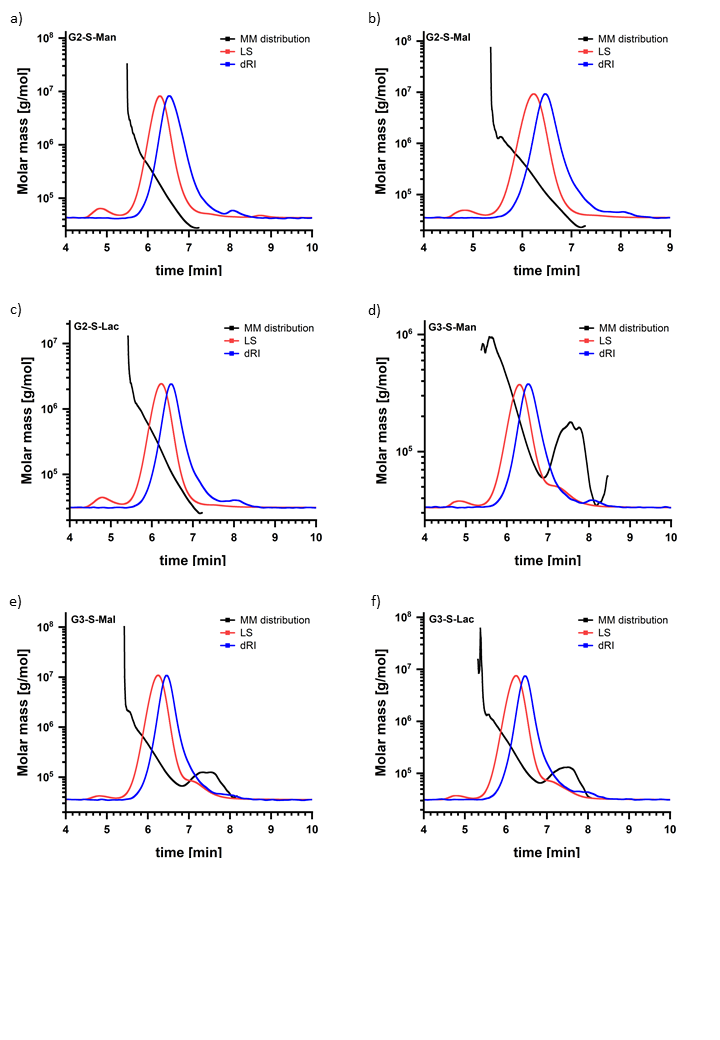


**Figure S51.** SEC chromatograms of pseudo-glycodendrimers (a) G2-S-Man, b) G2-S-Mal, c) G2-S-Lac, d) G3-S-Man, e) G3-S-Mal, f) G3-S-Lac).

Table S10. Degree of functionalization (DoF), molecular weight (M_w_, M_n_) and dispersity (Ð) for second and third generation pseudo-glycodendrimers with dye.

| **Analysis** | **G2-Dye-S-Man** | **G2-Dye-S-Mal** | **G2-Dye-S-Lac** | **G3-Dye-S-Man** | **G3-Dye-S-Mal** | **G3-Dye-S-Lac** |
| --- | --- | --- | --- | --- | --- | --- |
| DoF [%]^a^ | 98 | 95 | 95 | 97 | 95 | 95 |
| M_w_ [kg/mol]^a^ | 88.8 | 118.3 | 118.3 | 133.1 | 177.8 | 177.8 |
| Dye molecules per dendrimer^b^ | 2.5 | 2.4 | 2.5 | 3.3 | 3.4 | 3.2 |
| M_n_ [kg/mol] ^b^ | 85.0 | 110.1 | 97.0 | 130.5 | 203.2 | 152.7 |
| M_w_ [kg/mol]^b^ | 133.2 | 222.9 | 164.5 | 178.0 | 272.6 | 208.7 |
| Ð (M_w_/M_n_)^b^ | 1.57 | 1.68 | 1.69 | 1.36 | 1.34 | 1.37 |
| Dye molecules per dendrimer^d^ | 3.0 | 3.4 | 2.6 | 3.2 | 3.9 | 2.7 |
| ^a^Determination by ^1^H NMR spectroscopy. ^b^Determination by ^1^H NMR spectroscopy, fluorescence spectroscopy, and SEC. ^c^Determination by SEC. ^d^Determination by SEC and fluorescence spectroscopy. | | | | | | |

**6.9** **Characterization of pseudo-dendrimers and pseudo-glycodendrimers by UV-Vis and fluorescence spectroscopy**


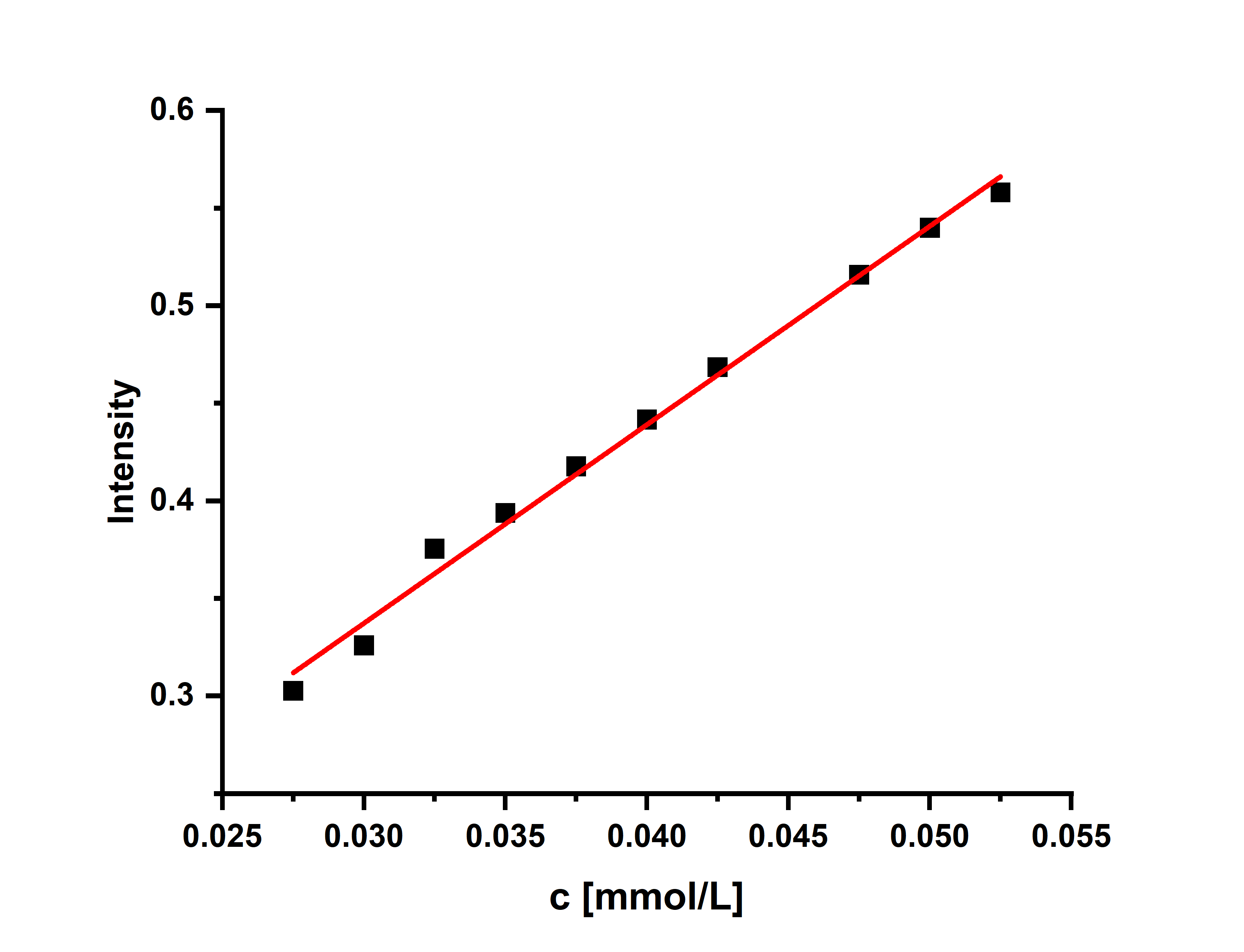


**Figure S52.** Calibration curve of AZDye 405 mixed with second generation of pseudo-glycodendrimer. R^2^ = 0.9916.


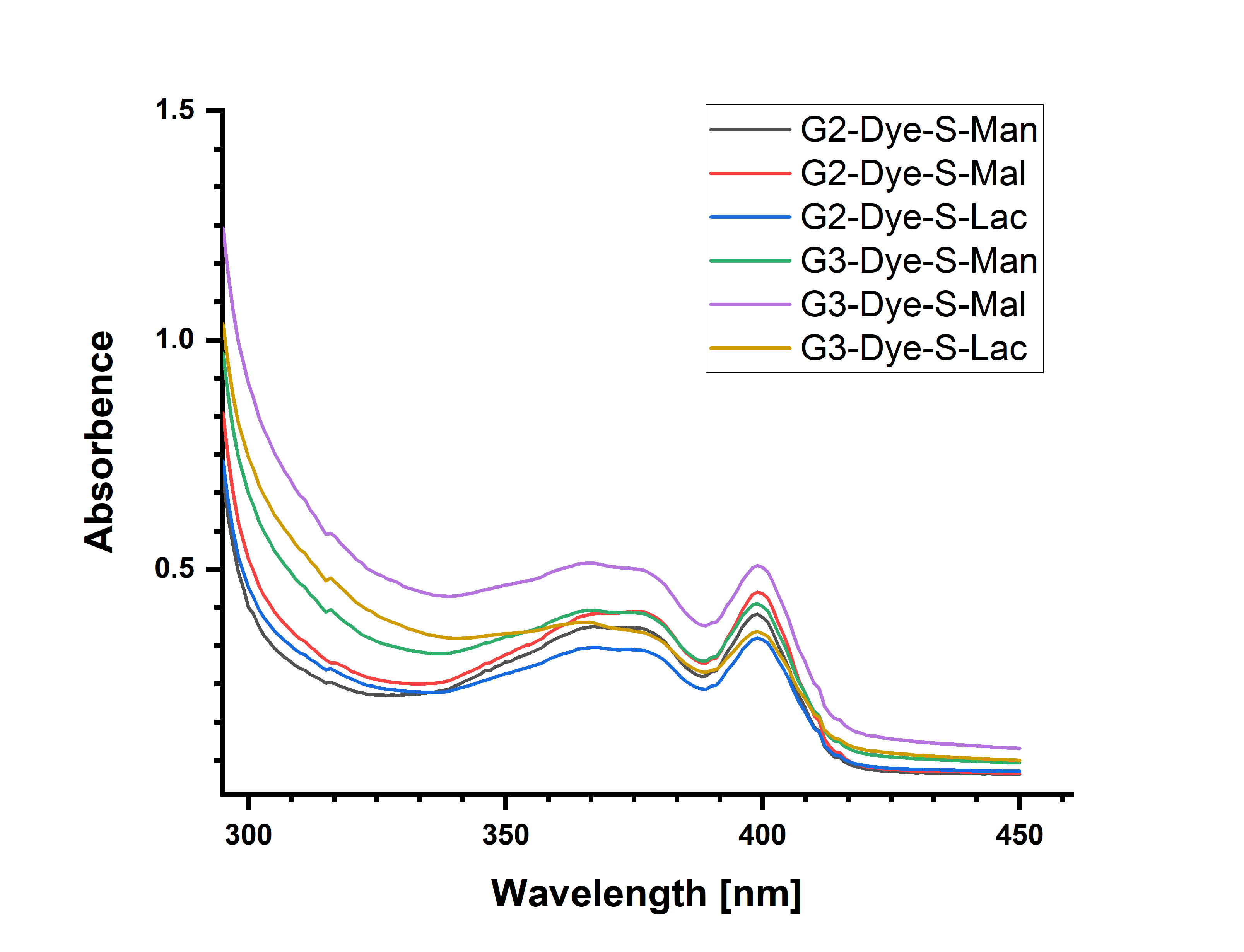


**Figure S53.** UV-VIS spectra of second and third generation pseudo-glycodendrimer labeled with AZ-Dye 405.


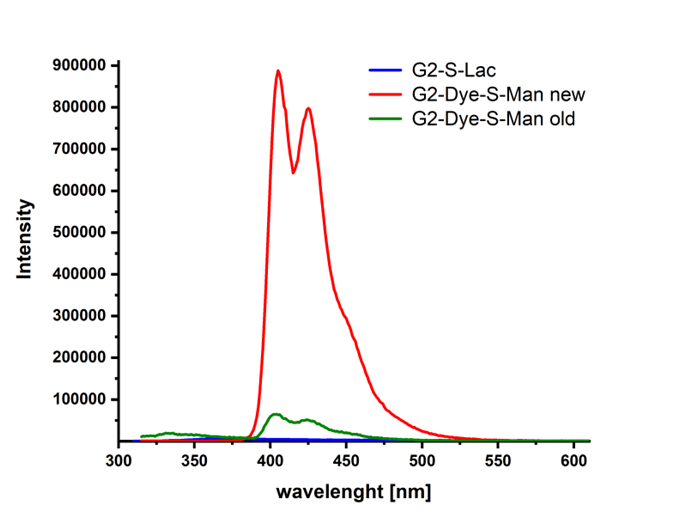


**Figure S54.** Comparison of the fluorescence spectra of G2‑S‑Lac (blue) as reference, G2‑Dye‑S‑Man new synthesis (red) and G2‑Dye‑S‑Man old synthesis (green).


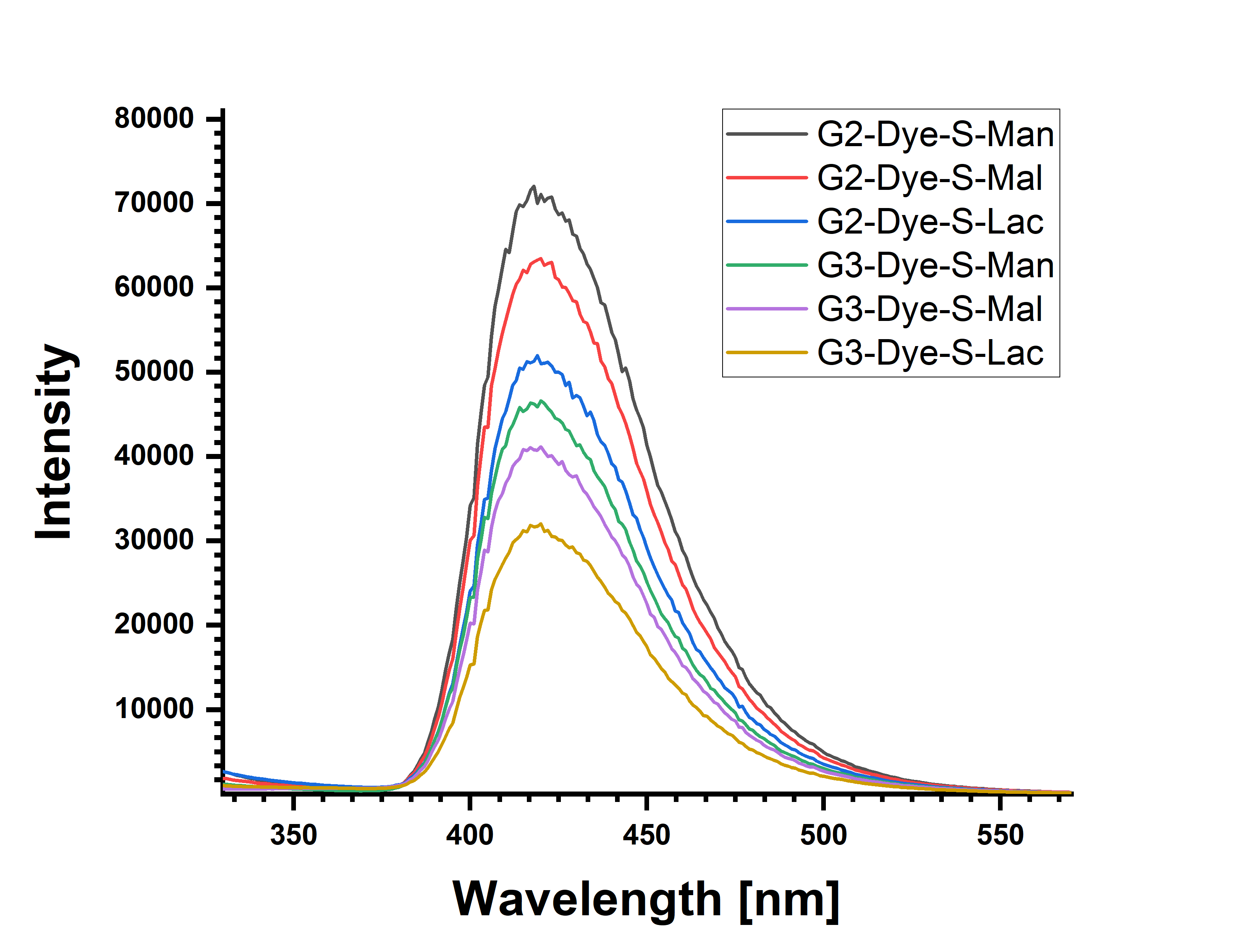


**Figure S55.** Fluorescence spectra of second and third generation pseudo-glycodendrimer labeled with AZ-Dye 405.


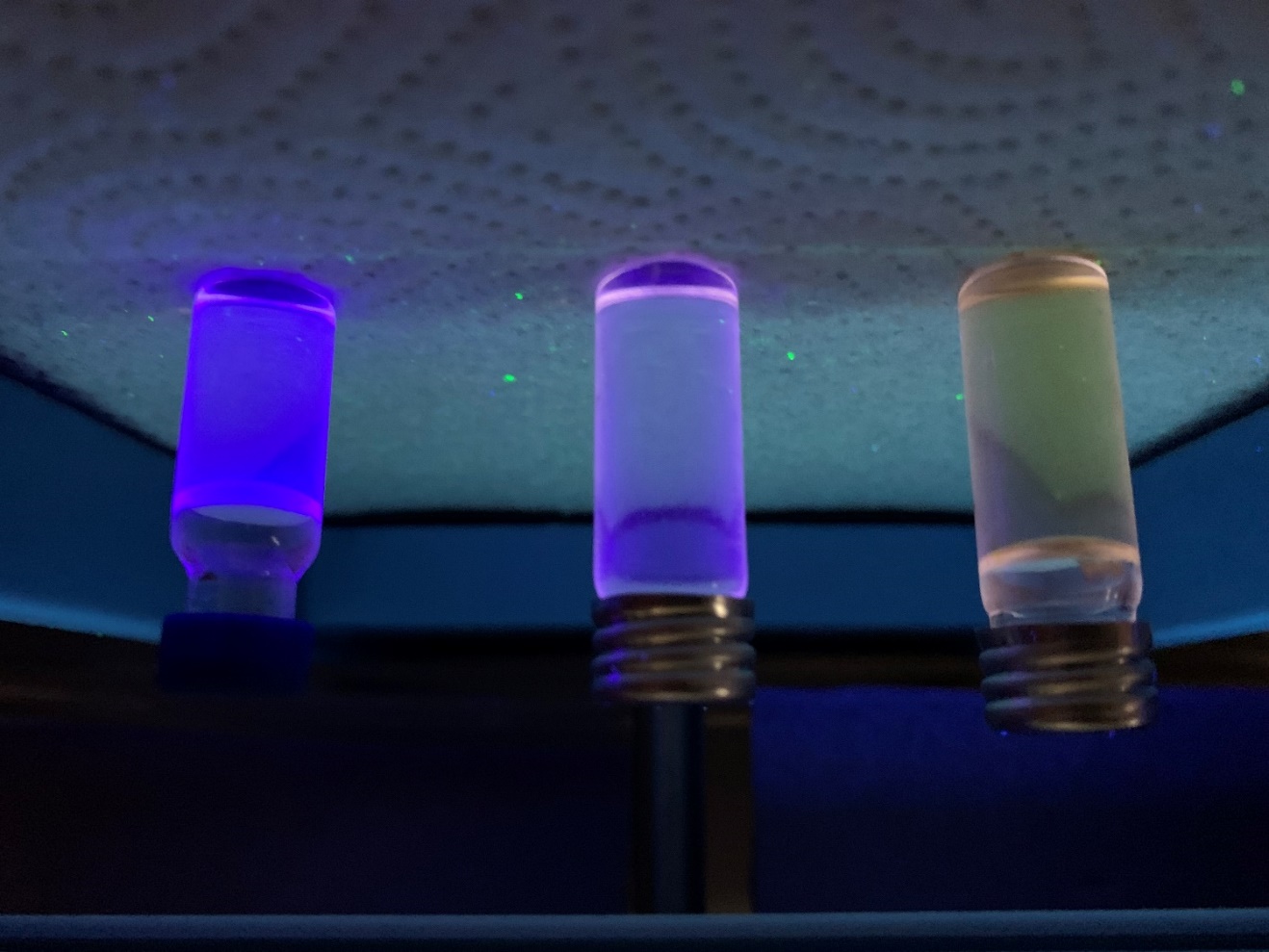


**Figure S56.** AzDye405 solution (right), G2-AzDye new synthesis (middle), G2-AzDye old synthesis (left). Illustration that the functionalization was not feasible under the old conditions.

**6.10 Characterization of pseudo-dendrimers and pseudo-glycodendrimers by DLS and FT-IR**


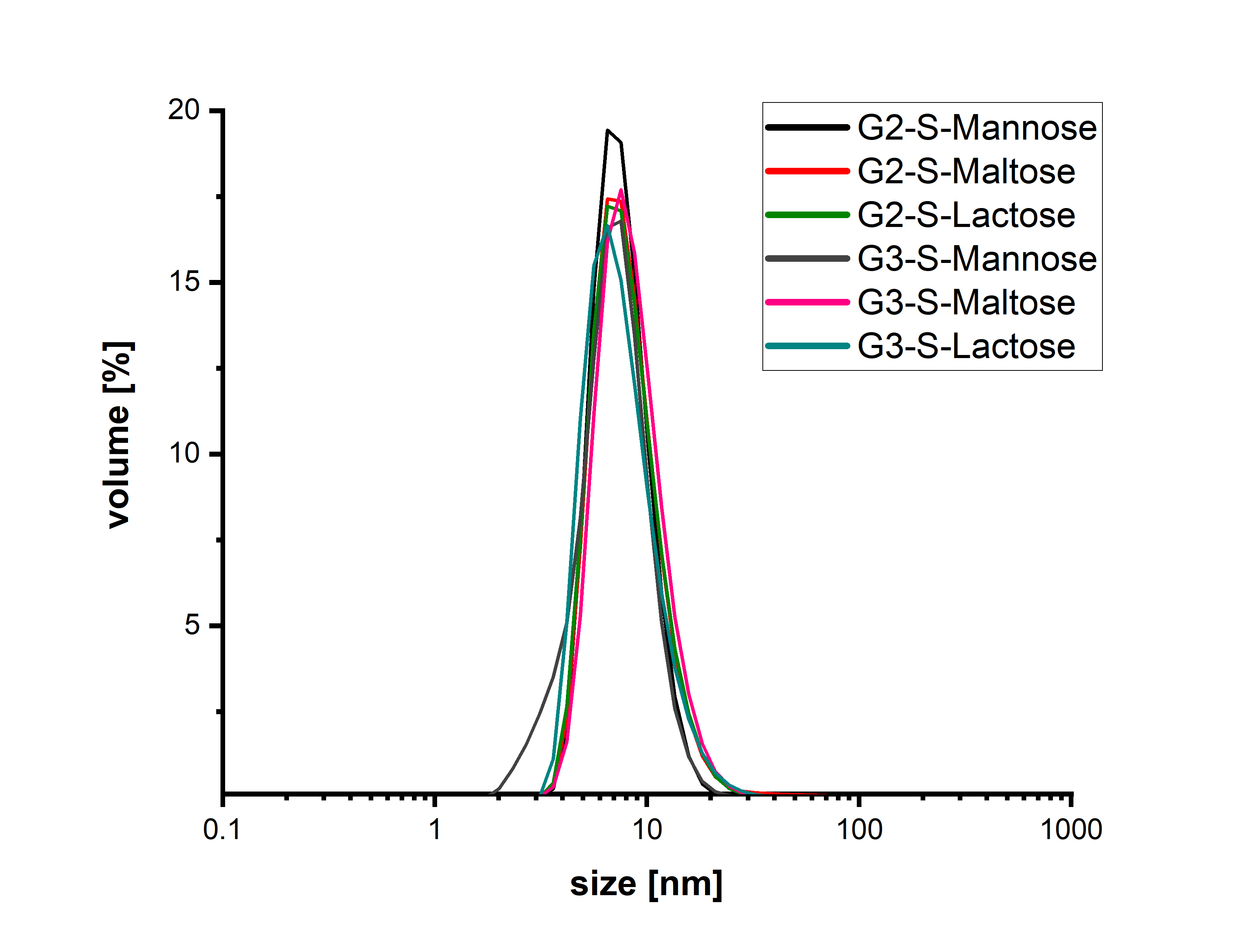


**Figure S57.** DLS measurements in 1 mM PBS buffer at 37 °C with second and third generation of pseudo-glycodendrimers.


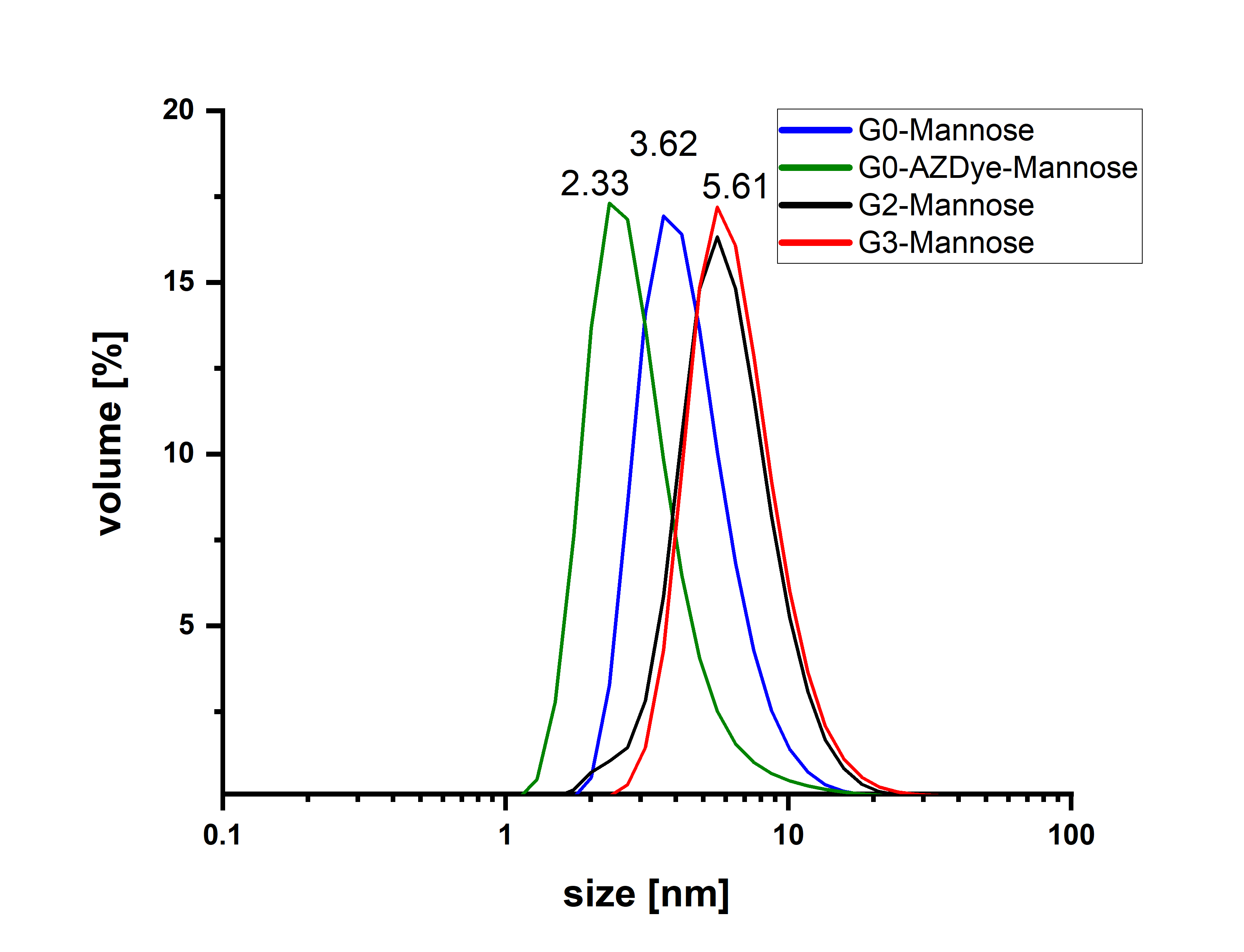


**Figure S58.** DLS measurements in 1 mM PBS buffer at 37 °C with different generations of pseudo-glycodendrimers with the same sugar.

Table S11. Hydrodynamic radius (R_h_)of different generations and sugar shells for pseudo-glycodendrimers.

| Analysis | G2-S-Man | G2-S-Mal | G2-S-Lac | G3-S-Man | G3-S-Mal | G3-S-Lac | G0-S-Man | G0-Dye-S-Man |
| --- | --- | --- | --- | --- | --- | --- | --- | --- |
| R_h_ [nm] | 3.25 | 3.25 | 3.25 | 3.75 | 3.75 | 3.25 | 1.8 | 1.15 |


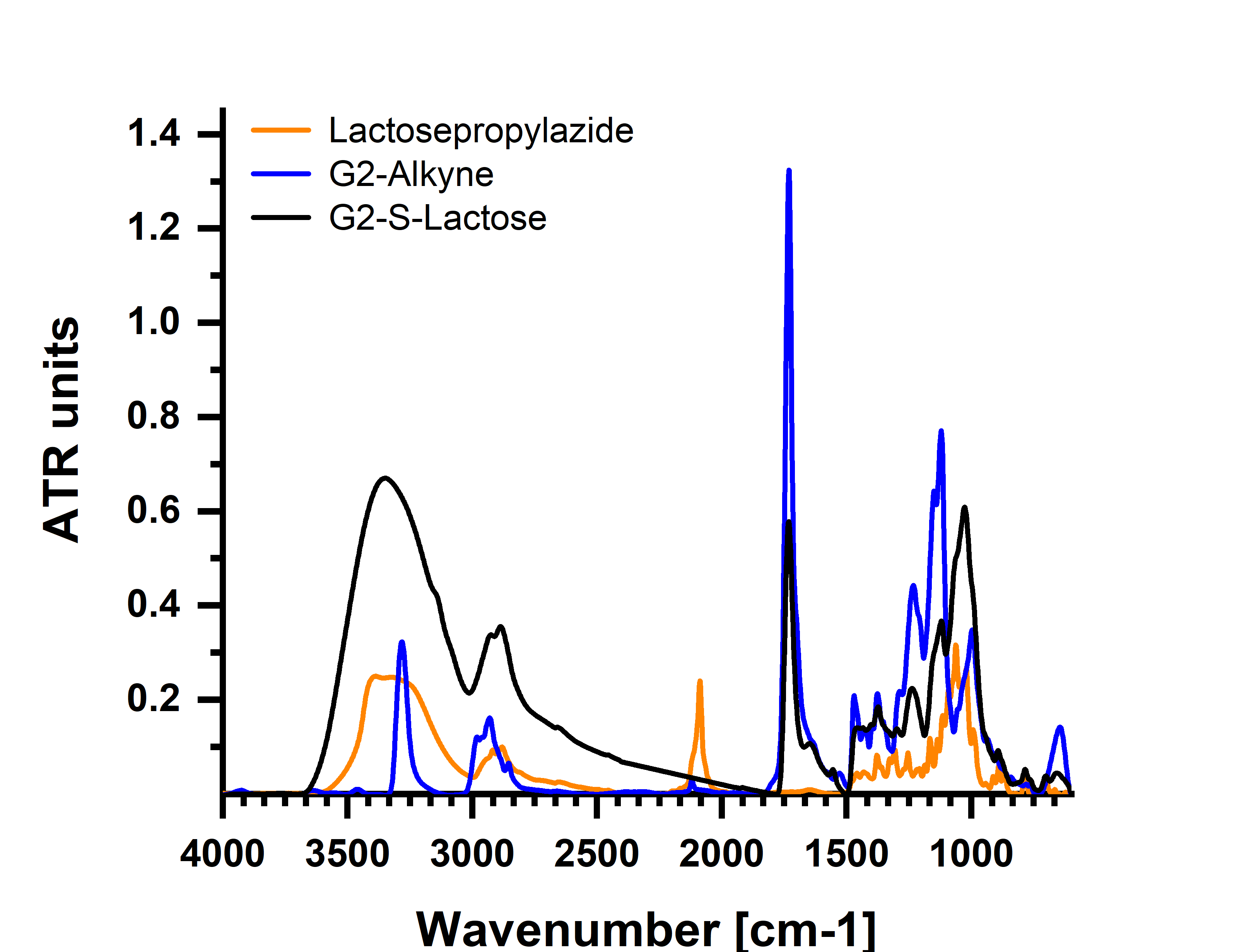


**Figure S59.** FTIR spectrum of lactose propylazide, G2-Alkyne and G2-S-Lactose.

**6.11** **Study of ThT assay on the interaction of pseudo-glycodendrimers toward amyloids**

**Figure S60.** Fluorescence measurement of ThT assay with 25 µM Aβ (1-40) in 1 mM PBS and G3-S-sugar in different concentrations from 1 to 10 µM (repeating experiment, n=8 averaged data without ± SD). Normalization of ThT fluorescence intensity of a) - d) in respect to the control, Aβ(1-40) (25 µM) + ThT (10 µM) (**Figure 5**), where the fluorescence (100%, black line) of the control is set as 1 (= reference).

### **Figure S61.** **Complexation Capacity of G2-S-Man with ThT: Fluorescence Analysis in the Presence and Absence of Aβ(1–40). (a)** Fluorescence emission spectra were recorded for the following samples: **Aβ(1–40)** (25 µM) in 1 mM PBS buffer in the absence of ThT (red line); **ThT alone** (10 µM, blue line; note: visually overlapped by the magenta line);**ThT (10 µM) in the presence of G2-S-Man** at 0.1 µM and 10 µM concentrations (magenta and olive lines, respectively).**(b)** To facilitate comparison, fluorescence intensities from panel (a) were normalized relative to the control sample of **Aβ(1–40)** (25 µM) incubated with **ThT (10 µM)**, which is assigned a reference value of 100% fluorescence (black line, normalized to 1.0). The normalized data reveal overlapping signals for **ThT alone** (10 µM, blue line), **Aβ(1–40) alone** (25 µM, red line), and **G2-S-Man + ThT** at 0.1 µM (magenta line).

There are negligible interactions of ThT and **G2-S-Man**, leading to very low ThT fluorescence (3%), compared to the intercalation of fluorescent ThT in the formed Aβ (1-40) fibrils (**Figure S61b**; 100%), leading to intense fluorescence of ThT.

**6.12** **Molecular modelling of simplified structures for pseudo-glycodendrimers, G2-Sugar and G3-Sugar**

**Figure S62**. Molecular modelling of **G2-S-Mal** and **G3-S-Mal** with the molecular shape of linear dendronized bottle brushes.

**6.13 Visualization of fibrils and aggregates of Aβ(1-40) in presence and absence of G2-S-Lac by cryo-TEM**

**Figure S63**. Cryo-TEM images of Aβ(1-40) (5 µM) solution after 24 h in a frozen state; results of ThT assay for 25 µM of Aβ(1-40) shown in **Figure 6f**. Cryo-TEM images validated from different spots of TEM grid. (A - D) Aβ(1-40) nanofibers and fibrils are visible. (C) Enlargement from (B) is shown; dotted square in (B).

**Figure S64**. Cryo-TEM of Aβ(1-40) (25 µM) in presence of G2-S-Lac (2.5 µM) for validating the solution state of Aβ(1-40) in ThT assay after 24 h in a frozen state; results of ThT assay shown in **Figure 6c**. Left: origin cryo-TEM image. Right: improved visualization mode of cryo-TEM image left. Red arrows indicate ice crystals.

**Figure S65**. Cryo-TEM of Aβ(1-40) (25 µM) in presence of G2-S-Lac (2.5 µM) for validating the solution state of Aβ(1-40) in ThT assay after 24 h in a frozen state; results of ThT assay shown in **Figure 6c**. Left: cryo-TEM image with 500 nm scale bar. Right: cryo-TEM image with 200 nm scale bar; enlarged area from cryo-TEM image left.

It looks likely that nanofibers and fibrils from pure Aβ(1-40) (5 µM) are much longer compared to them in the presence of G2-S-Lac at 2.5 µM (**Figure S65**). 25 µM of pure Aβ(1-40) cannot be visualized due to huge network formation of fibrils etc.

**Figure S66**. Cryo-TEM images of Aβ(1-40) (25 µM) in presence of G2-S-Lac (5 µM) for validating the solution state of Aβ(1-40) in ThT assay after 24 h in a frozen state; results of ThT assay shown in **Figure 6d**. (A) Overview of spots on selected grid TEM. (B) Another selected overview of spots on TEM grid. (C) Enlarged spot from cryo-TEM (B). (D) Further enlarged spot from cryo-TEM (C).

**Figure S67**. Cryo-TEM images of Aβ(1-40) (25 µM) in presence of G2-S-Lac (5 µM) for validating the solution state of Aβ(1-40) in ThT assay after 24 h in a frozen state; results of ThT assay shown in **Figure 6d**. Cryo-TEM images validated from another TEM grid. (A) Black spot can be identified as smaller aggregate of biohybrid structure of Aβ(1-40) and G2-S-Lac. (B) Another selected overview of spots on TEM grid. (C) Enlarged spot from cryo-TEM image (B), showing enlarged undefined aggregates of biohybrid structure of Aβ(1-40) and G2-S-Lac. (D) Enlarged undefined aggregates of biohybrid structure of Aβ(1-40) and G2-S-Lac, validated from another spot on TEM grid.

**Figure S68**. Cryo-TEM images of Aβ(1-40) (25 µM) in presence of G2-S-Lac (10 µM) for validating the solution state of Aβ(1-40) in ThT assay after 24 h in a frozen state; results of ThT assay shown in **Figure 6f**. Cryo-TEM images validated from different spots on TEM grid. (A) - (D) Smaller and larger aggregates of biohybrid structure of Aβ(1-40) and G2-S-Lac visible. Uncontrolled aggregation processes between Aβ(1-40) and G2-S-Lac are visible. Similar dimensions of aggregates compared to **Figure R5**, showing aggregates with 5 µM of G2-S-Lac.

There are no fibril formations of Aβ(1-40) in the presence of G2-S-Lac at 5 and 10 µM (**Figures S66-S68**). There are few indications of uncontrolled aggregates between Aβ(1-40) and G2-S-Lac. This smoothly indicates that G2-S-Lac undergoes non-covalent interactions with Aβ(1-40), leading to the suppression of undesired nanofibers, fibrils and plaque formation at 5 and 10 µM, but only the formation of aggregates between Aβ(1-40) and G2-S-Lac (e.g. **Figures S67d**, **S68D**) and smaller particles of them (e.g. **Figure S67A, S68D**).

**Figure S69.** Determination of fibril´s diameter (10 ±1 nm) of pure Aβ(1-40) (5 µM) by cryo-TEM.

1. **References**

Aso, E., Martinsson, I., Appelhans, D., Effenberg, C., Benseny-Cases, N., Cladera, J., Gouras, G., Ferrer, I., & Klementieva, O. (2019). Poly(propylene imine) dendrimers with histidine-maltose shell as novel type of nanoparticles for synapse and memory protection. *Nanomedicine: Nanotechnology, Biology and Medicine*, *17*, 198–209.

Bærentsen, R. L., Nielsen, S. V., Skjerning, R. B., Lyngsø, J., Bisiak, F., Pedersen, J. S., ... & Brodersen, D. E. (2023). Structural basis for kinase inhibition in the tripartite E. coli HipBST toxin–antitoxin system. *Elife*, *12*, RP90400.

Firdaus, S., Boye, S., Janke, A., Friedel, P., Janaszewska, A., Appelhans, D., Müller, M., Klajnert-Maculewicz, B., Voit, B., & Lederer, A. (2023). Advancing Antiamyloidogenic Activity by Fine-Tuning Macromolecular Topology. *Biomacromolecules*, *24*(12), 5797–5806. https://doi.org/10.1021/acs.biomac.3c00817

Firdaus, S., Geisler, M., Friedel, P., Banerjee, S., Appelhans, D., Voit, B., & Lederer, A. (2018). Glyco‐pseudodendrimers on a Polyester Basis: Synthesis and Investigation of Protein–Pseudodendrimer Interaction. *Macromolecular Rapid Communications*, *39*(16), 1800364. https://doi.org/10.1002/marc.201800364

Glatter, O. (1977). A new method for the evaluation of small-angle scattering data. *Journal of applied crystallography*, *10*(5), 415-421.

Gvazava, N., Konings, S. C., Cepeda-Prado, E., Skoryk, V., Umeano, C. H., Dong, J., Silva, I. A. N., Rylander Ottosson, D., Leigh, N. D., Wagner, D. E., & Klementieva, O. (2023). Label-Free High-Resolution Photothermal Optical Infrared Spectroscopy for Spatiotemporal Chemical Analysis in Fresh, Hydrated Living Tissues and Embryos. Journal of the American Chemical Society, *145*, 24796−24808)

Klementieva, O., Willén, K., Martinsson, I., Israelsson, B., Engdahl, A., Cladera, J., Uvdal, P., & Gouras, G. K. (2017). Pre-plaque conformational changes in Alzheimer’s disease-linked Ab and APP. Nature Communications, *8*, 14726.

Lyngsø, J., & Pedersen, J. S. (2021). A high-flux automated laboratory small-angle X-ray scattering instrument optimized for solution scattering. *Journal of applied crystallography*, *54*(1), 295-305.

Pedersen, J. S., Hansen, S., & Bauer, R. (1994). The aggregation behavior of zinc-free insulin studied by small-angle neutron scattering. *European biophysics journal*, *22*, 379-389.

Sulatskaya, A. I., Lavysh, A. V., Maskevich, A. A., Kuznetsova, I. M., & Turoverov, K. K. (2017). Thioflavin T fluoresces as excimer in highly concentrated aqueous solutions and as monomer being incorporated in amyloid fibrils. Scientific Reports, *7*, 2146.
